# Supplementary material for: Eremophilane-Type Sesquiterpenes from a Marine-Derived Fungus Penicillium Copticola with Antitumor and Neuroprotective Activities
Source: Mar Drugs. 2022 Nov 13;20(11):712. doi: 10.3390/md20110712 (PMC9698232; doi:10.3390/md20110712)

Eremophilane-type sesquiterpenes from a marine-derived *Penicillium copticola* fungus with antitumor and neuroprotective activities

Supporting Information

ITS-sequence of fungus

CTCTGGGTCCACCTCCCACCCGTGTTTTCCGAACCCTGTTGCTTCGGCGGG
CCCGCCTCACGGCCGCCGGGGGGCTTCCGCCCCCGGGCCCGCGCCCGCCG
AAGACACCTGTGAACGCTGTCTGAAGTTGCAGTCTGAGAAACTAGCTAGA
TTAGTTAAAACTTTCAACAACGGATCTCTTGGTTCCGGCATCGATGAAGA
ACGCAGCGAAATGCGATAACTAATGTGAATTGCACAATTCAGGAATCATC
GAGTCTTTGAACGCACATGGCGCCCTCTGGTATTCCGGAGGGCATGCCTG
TCCGAGCGTCATTGCTGCCCTCAAGCACGGCTTGTGTGTTGGGCCCCACCC
CCCCGCCCTGCCCGAAGGCGGGGGCGCGGGGGGGGGGCAGAACCGAGCC
CAATCCTAATGACGAATGGAGGTGAGACCACACCTGTTTGTATTGACCC
GATCGCCAGCCGCCTGACCCACCTAAATTATTTTTTCAGGTTGA

UPLC screening:

t/min	（mL/min）	A (MeCN)	B (H_2O +0.04%TFA）
0	0.4	10	90
7	0.4	100	0
8	0.4	100	0
8.01	0.4	10	90
10	0.4	10	90

WZXY-SX-10-26-1

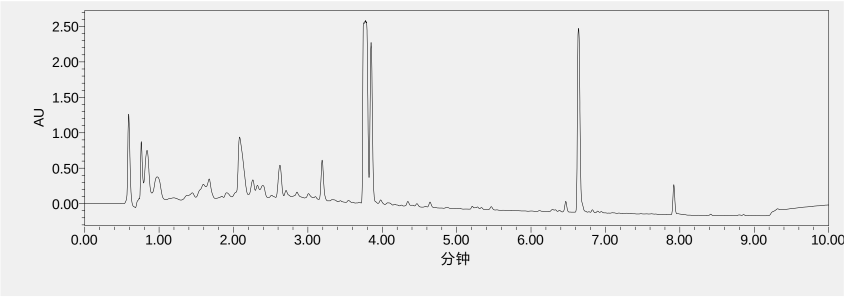

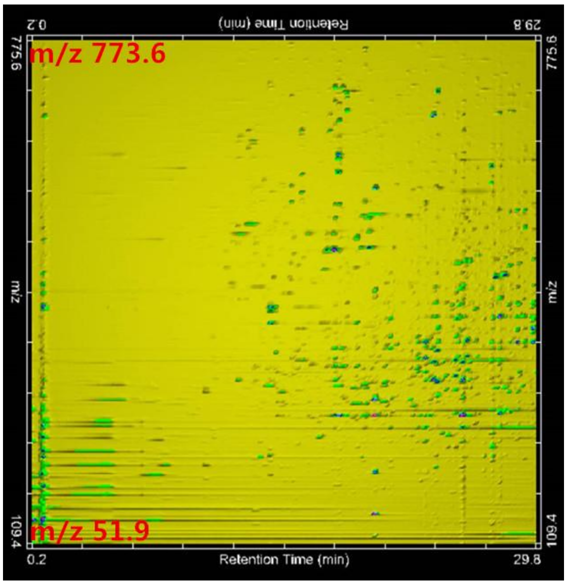

LC-MS spectrum of EtOAc extract of WZXY-SX-10-26-1 fungal strain

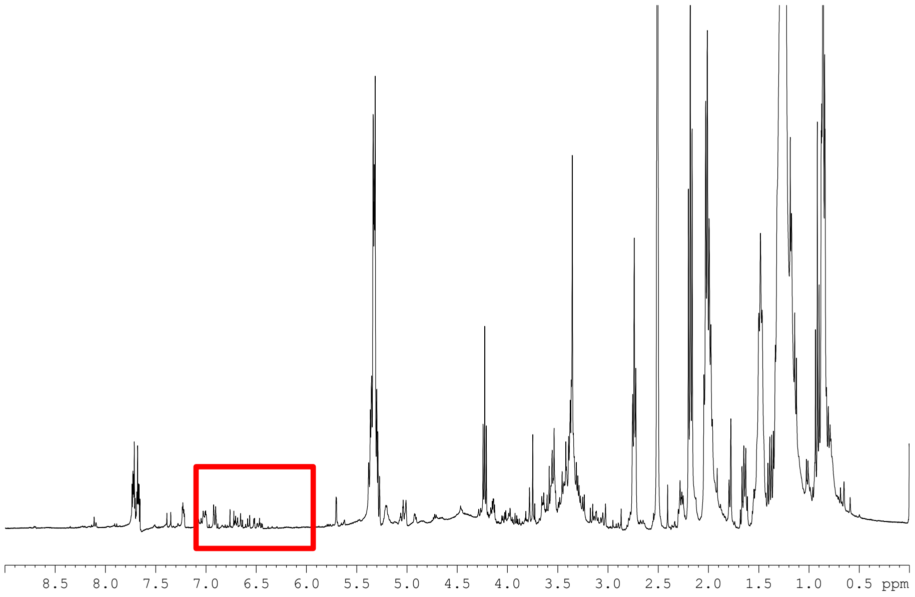

^{1}H NMR spectrum of EtOAc extract of WZXY-SX-10-26-1 fungal strain

Figure S1. IR spectrum of **1**

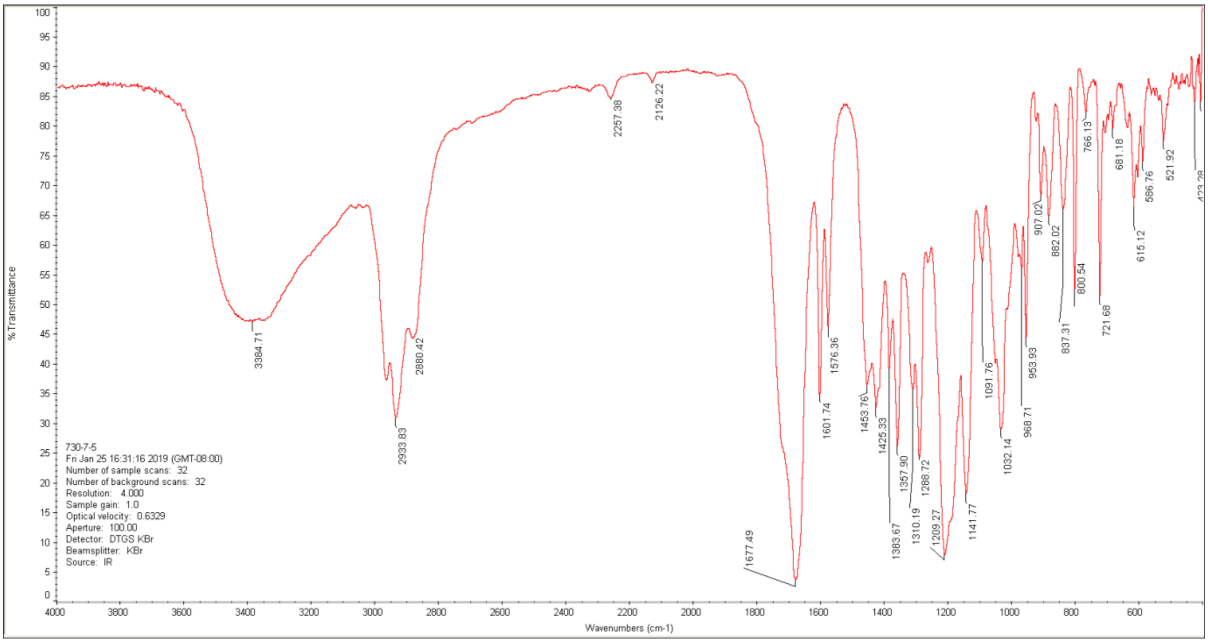

Figure S2. ^{1}H NMR (600 MHz) spectrum of **1** in DMSO-*d*6

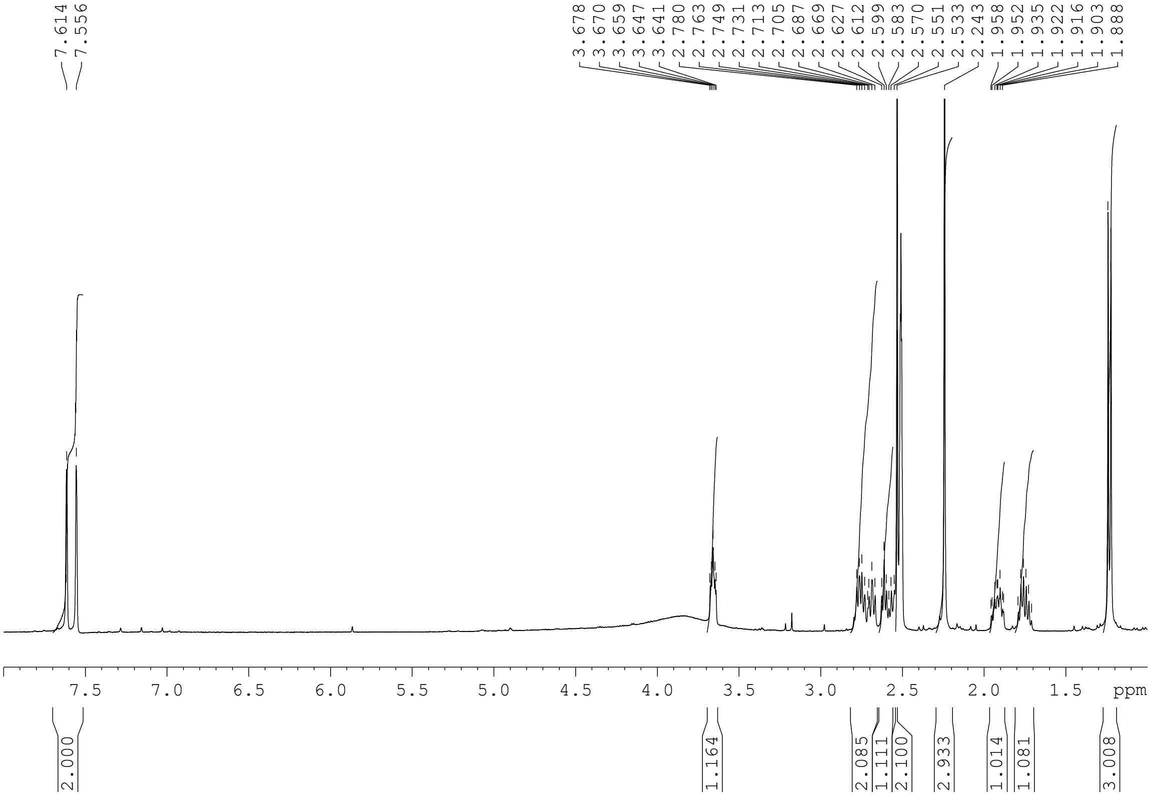

Figure S3. ^{13}C NMR (150 MHz) spectrum of **1** in DMSO-d_6

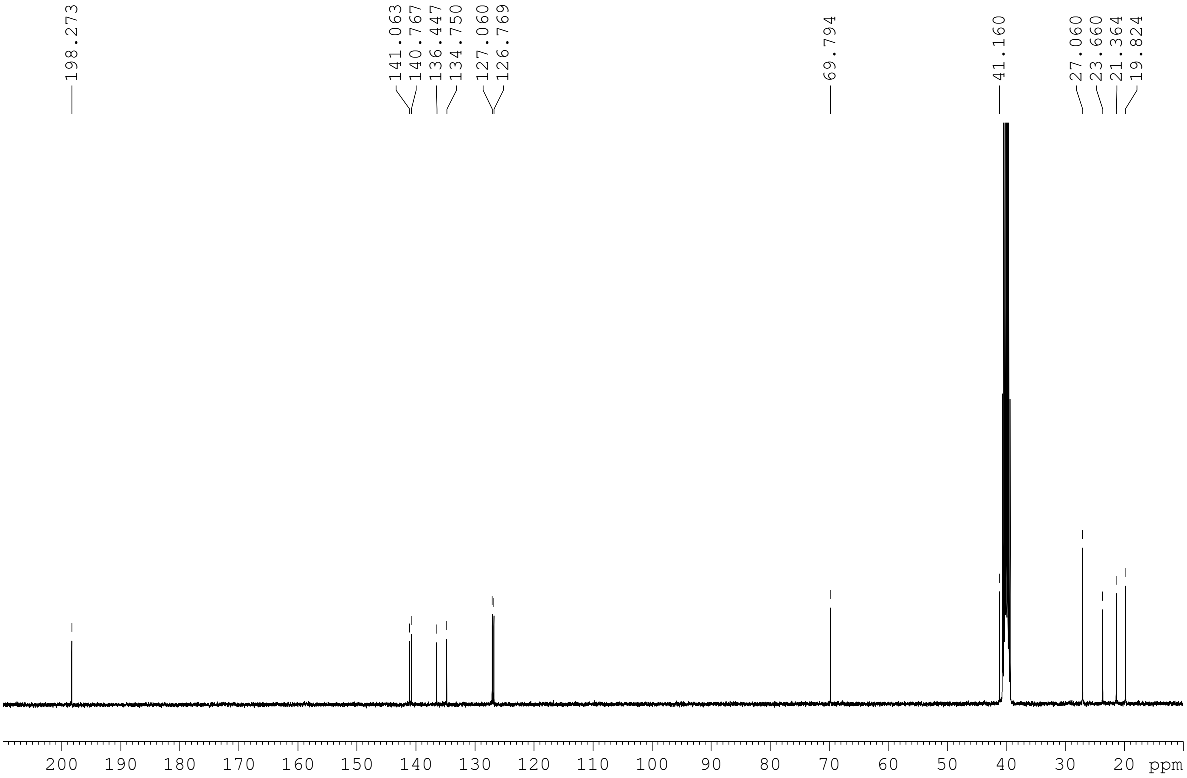

Figure S4. ^1H-^1H COSY spectrum of **1** in DMSO-d6

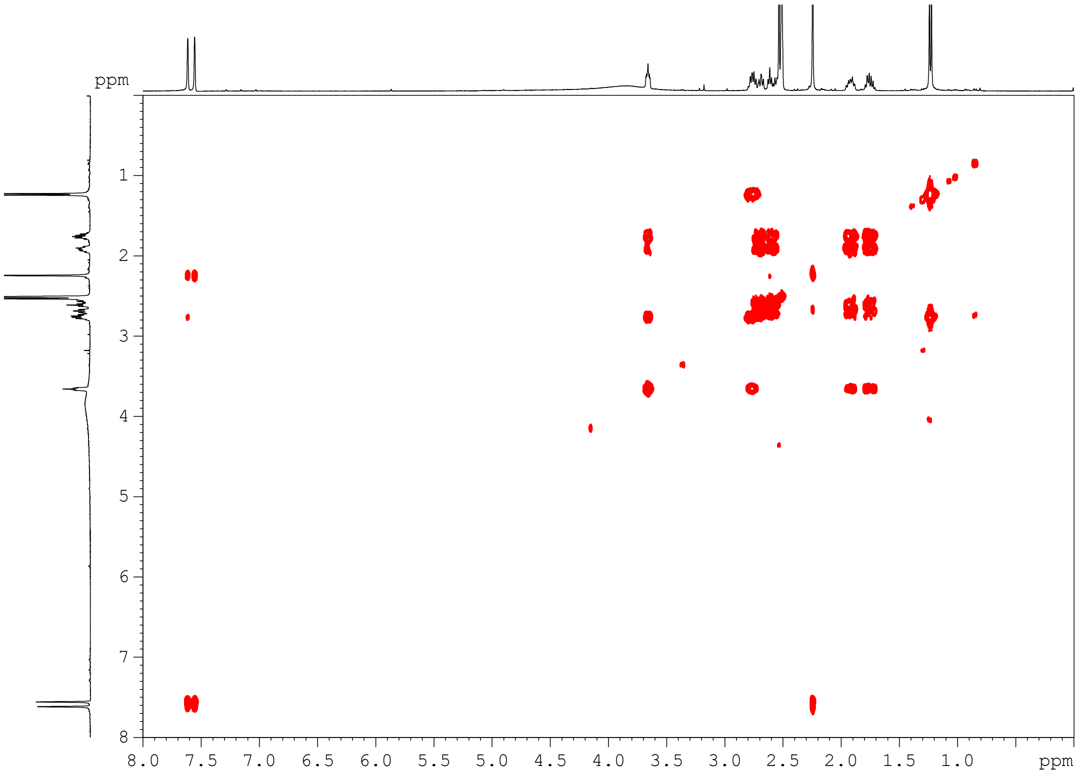

Figure S5. HSQC spectrum of **1** in DMSO-*d6*

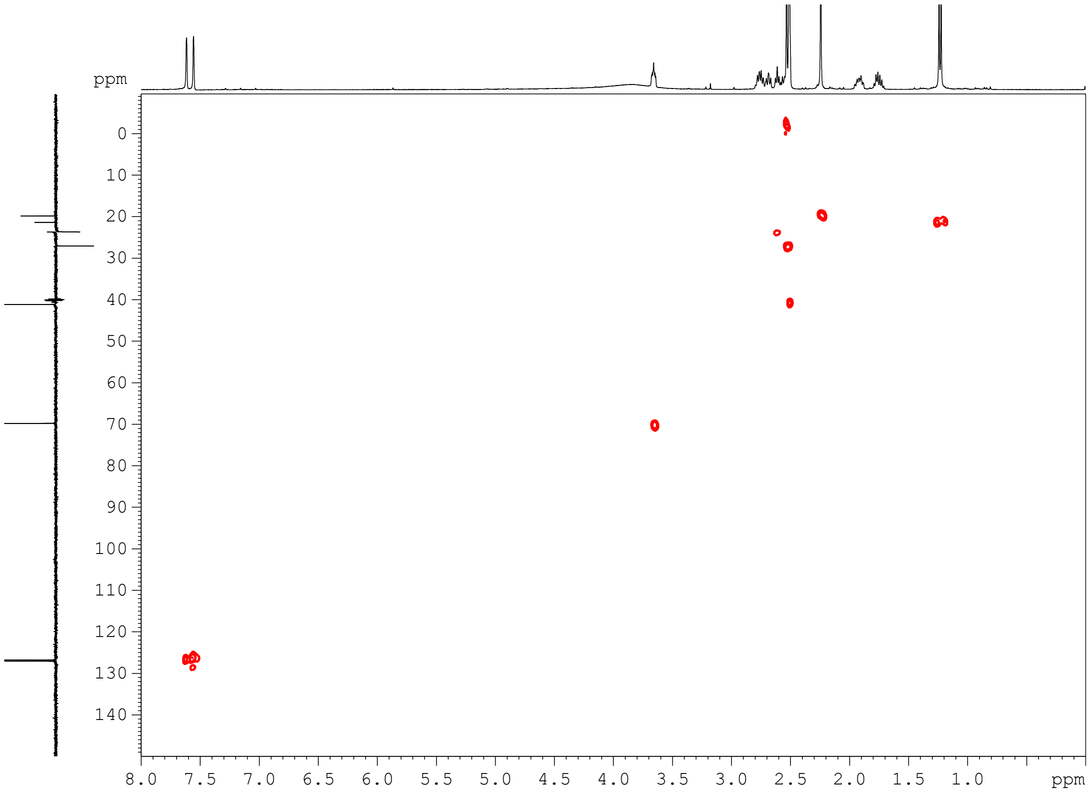

Figure S6. HMBC spectrum of **1** in DMSO-*d*6

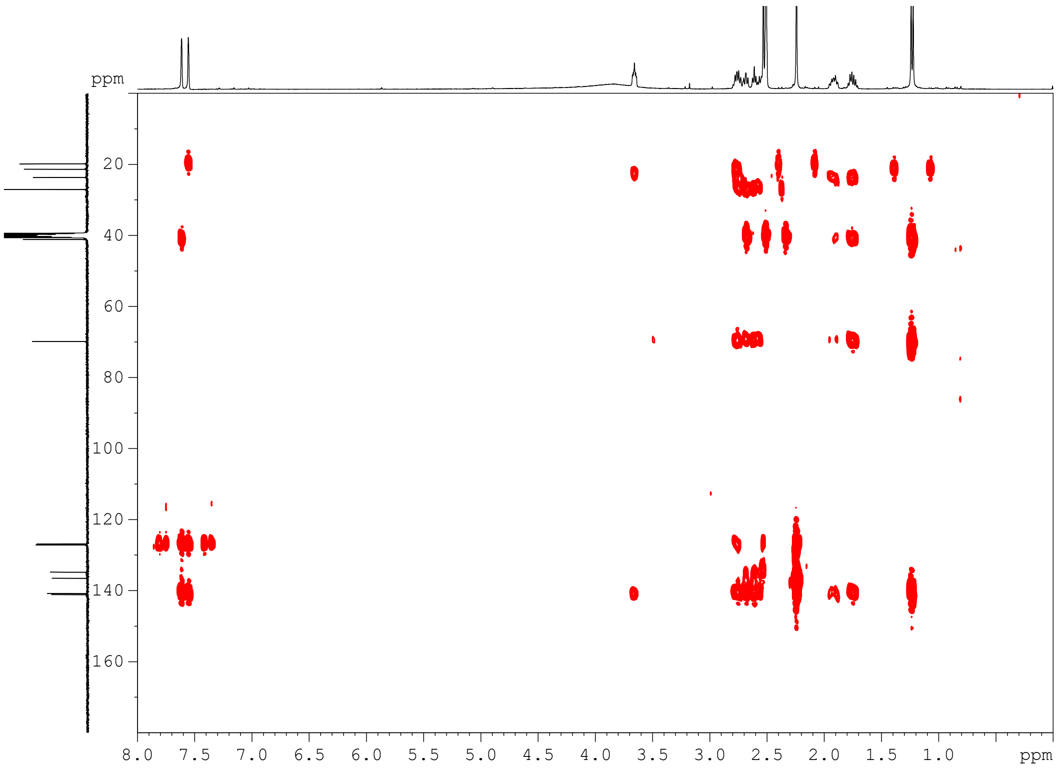

Figure S7. NOESY spectrum of **1** in DMSO-*d6*

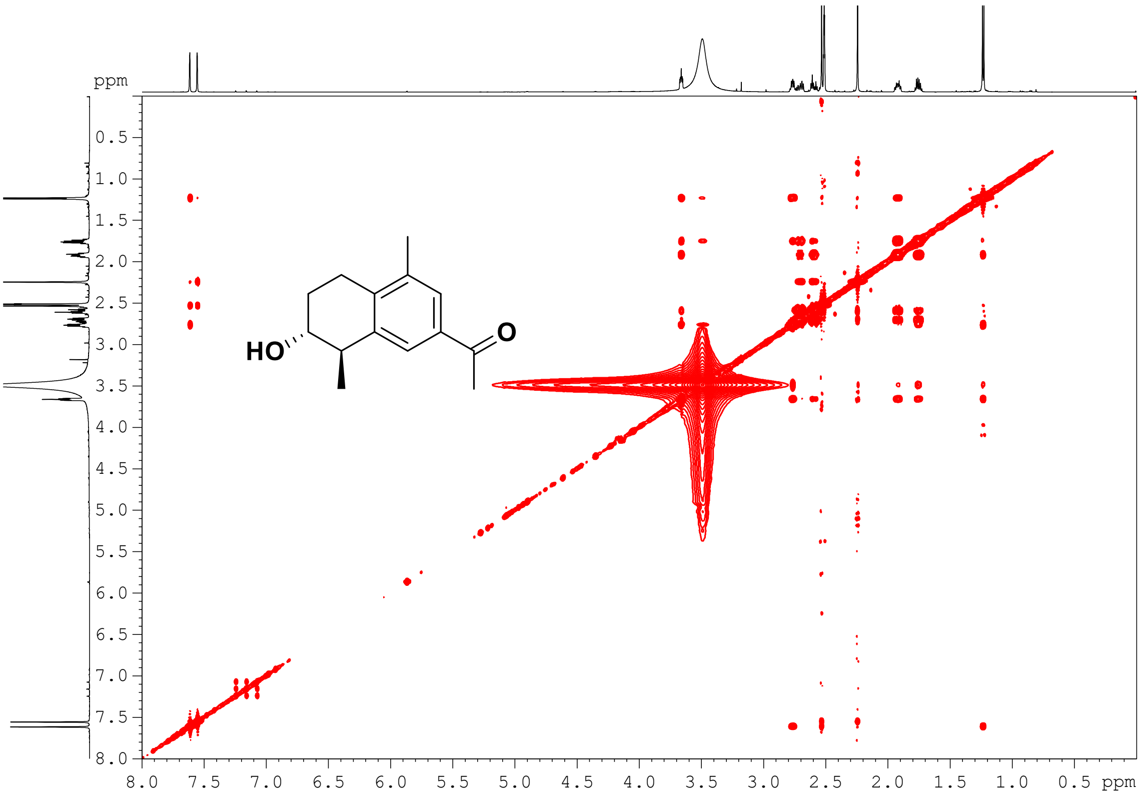

Figure S8. HRESIMS spectrum of **1**

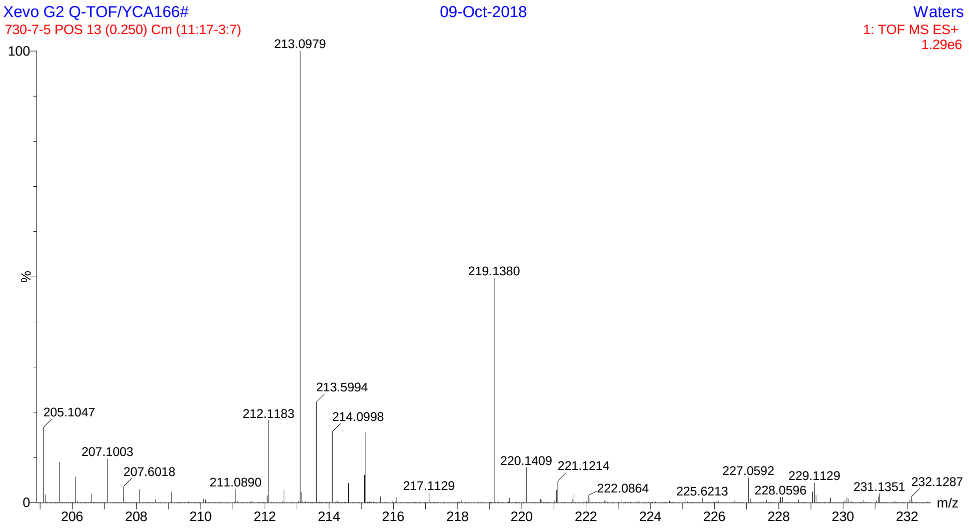

Mass	Calc. Mass	mDa	PPM	DBE	i-FIT	Norm	Conf(%)	Formula
219.1380	219.1385	-0.5	-2.3	5.5	767.1	n/a	n/a	$C_{14}H_{19}O_2$

Figure S9. IR spectrum of **2**

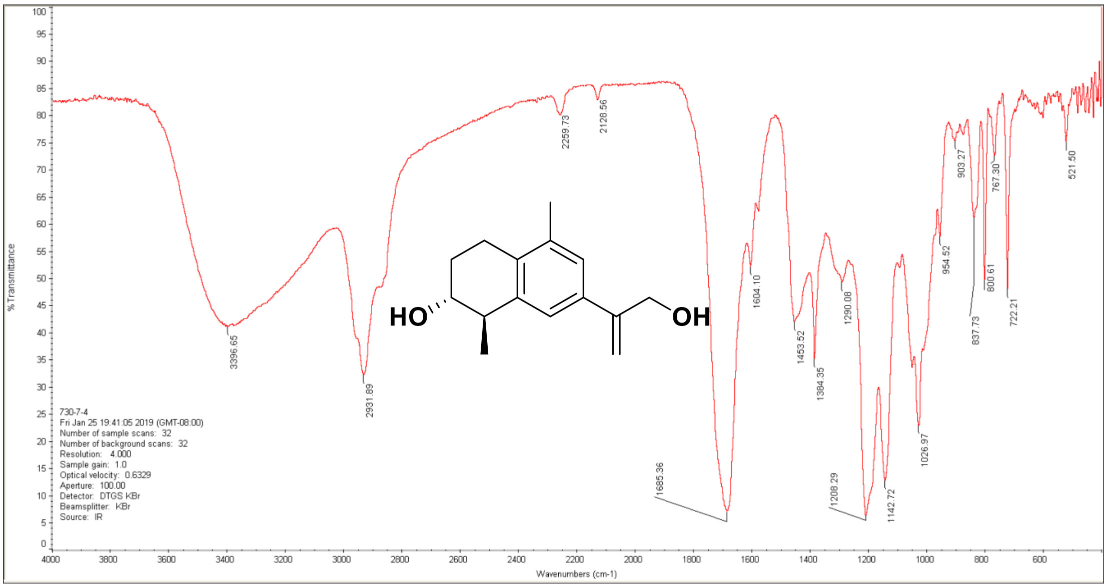

Figure S10. ^{1}H NMR (600 MHz) spectrum of **2** in DMSO-*d6*

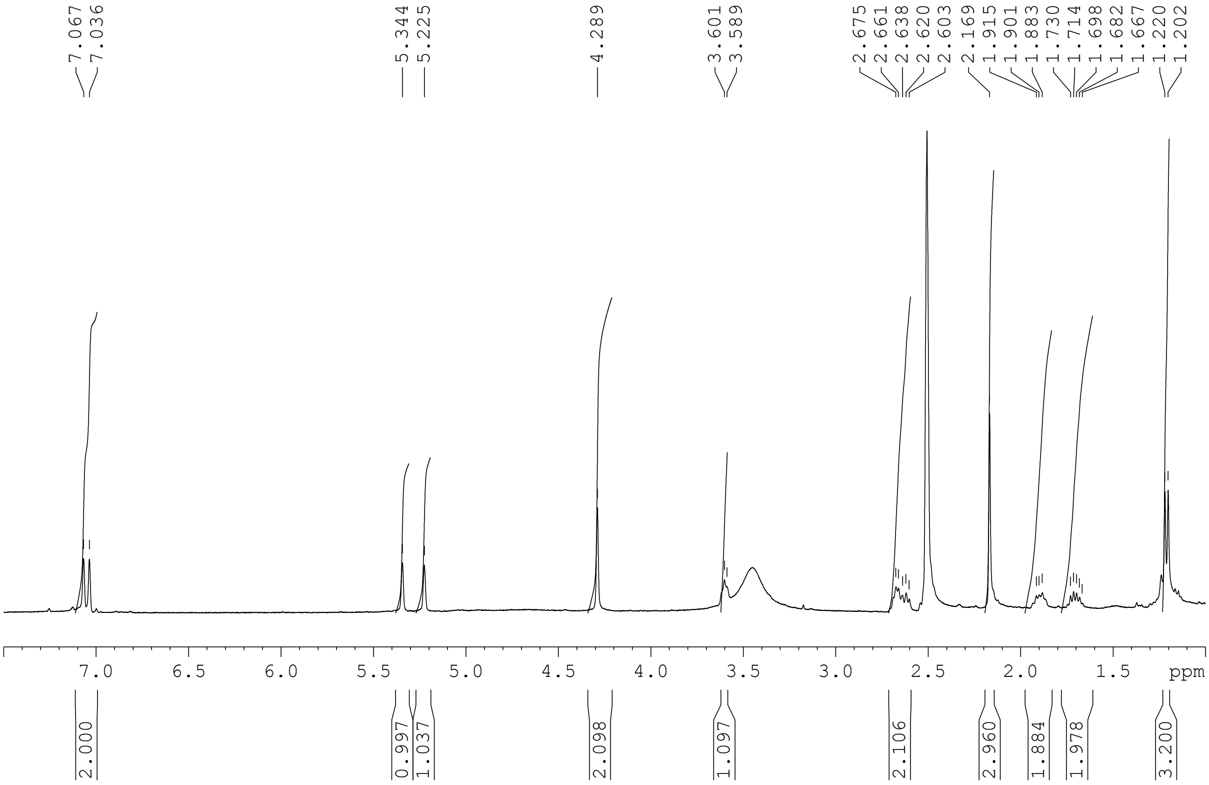

Figure S11. ^{13}C NMR and DEPT (150 MHz) spectra of **2** in DMSO-d_6

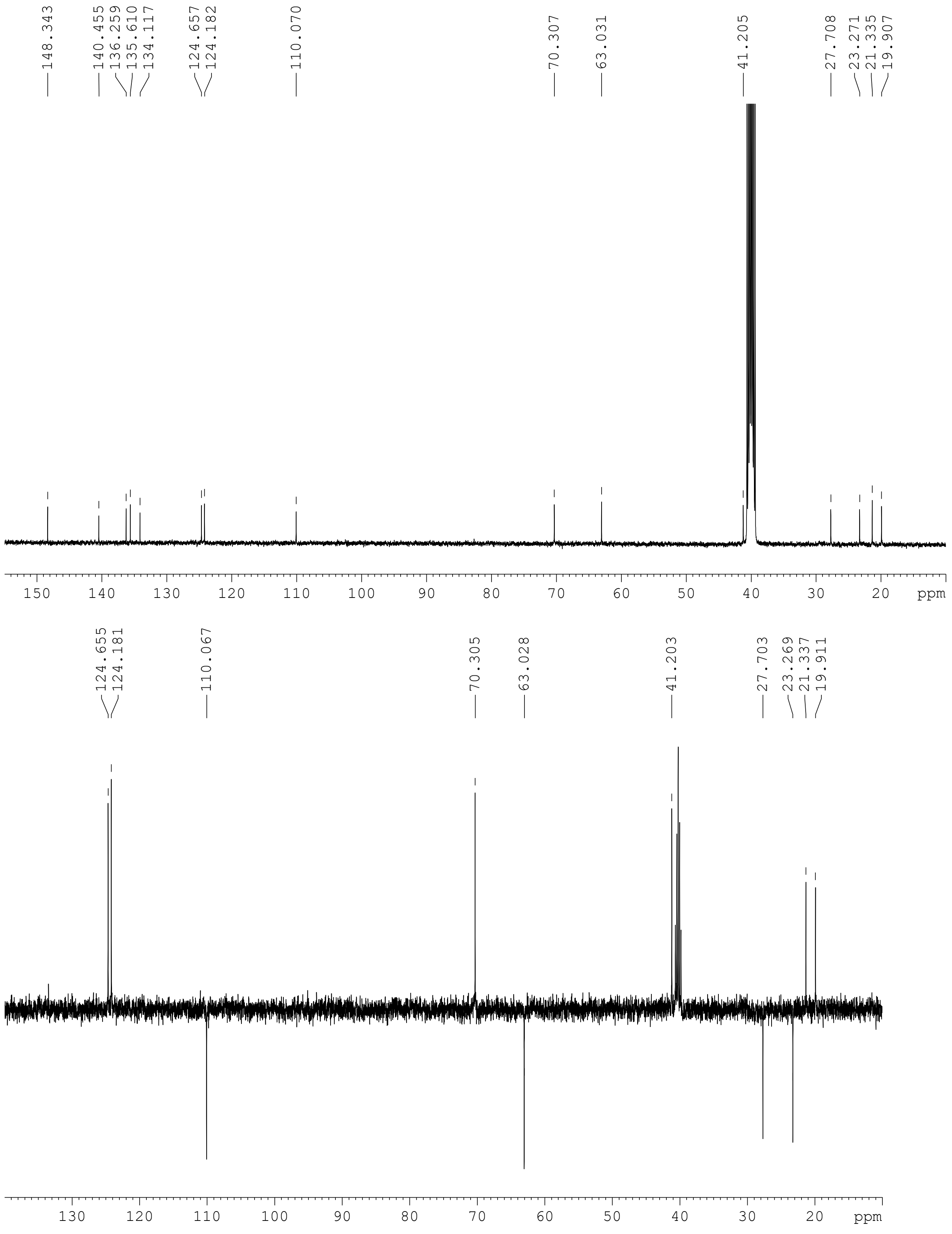

Figure S12. ^{1}H-^{1}H COSY spectrum of **2** in DMSO-*d*6

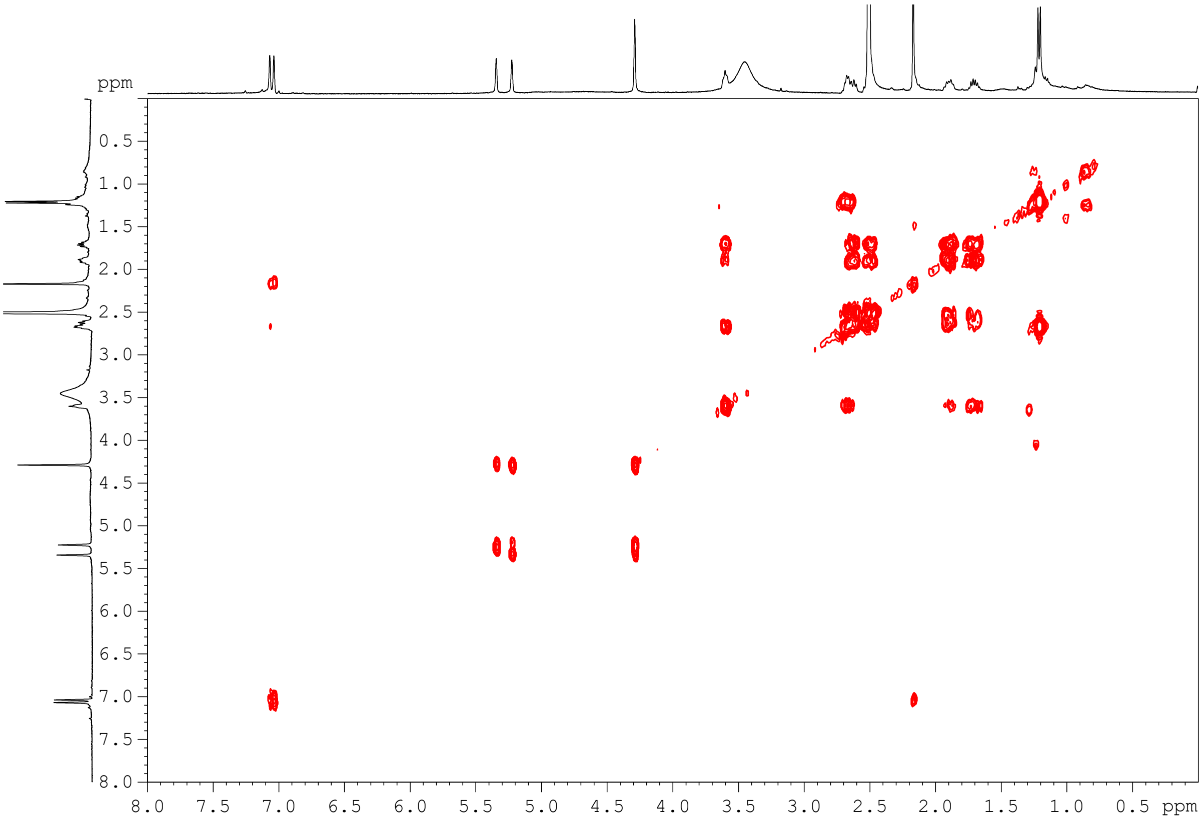

Figure S13. HSQC spectrum of **2** in DMSO-*d*6

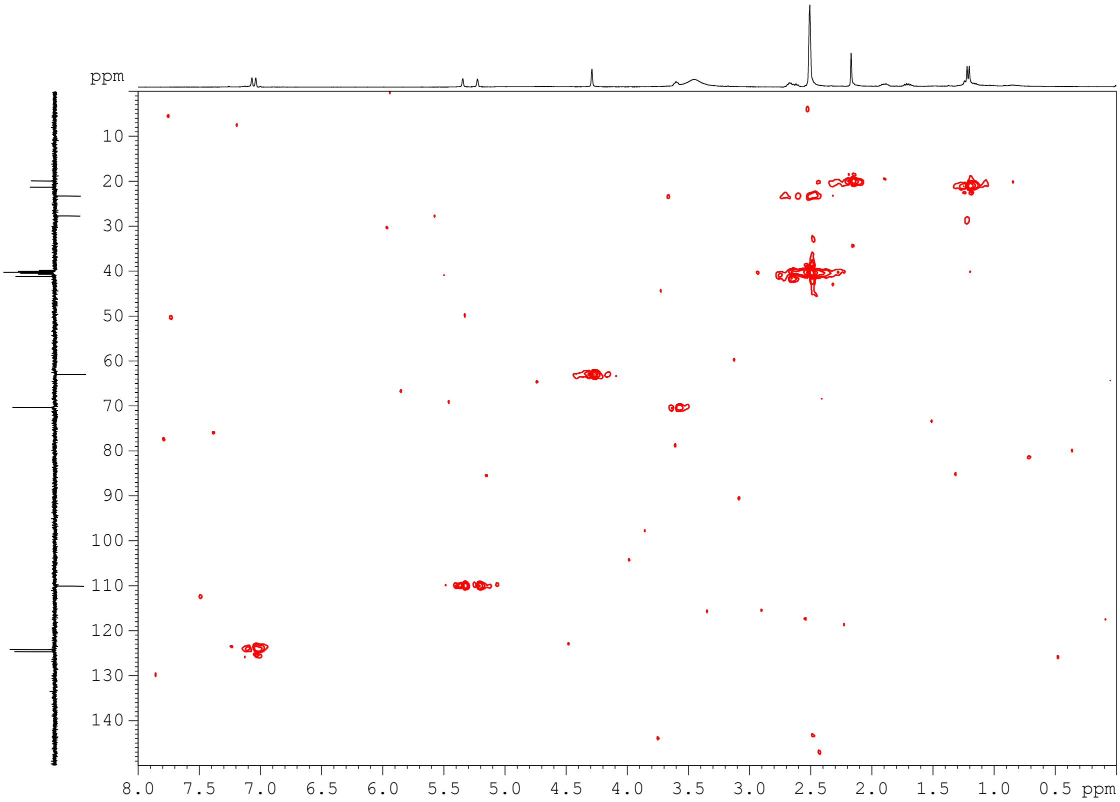

Figure S14. HMBC spectrum of **2** in DMSO-*d6*

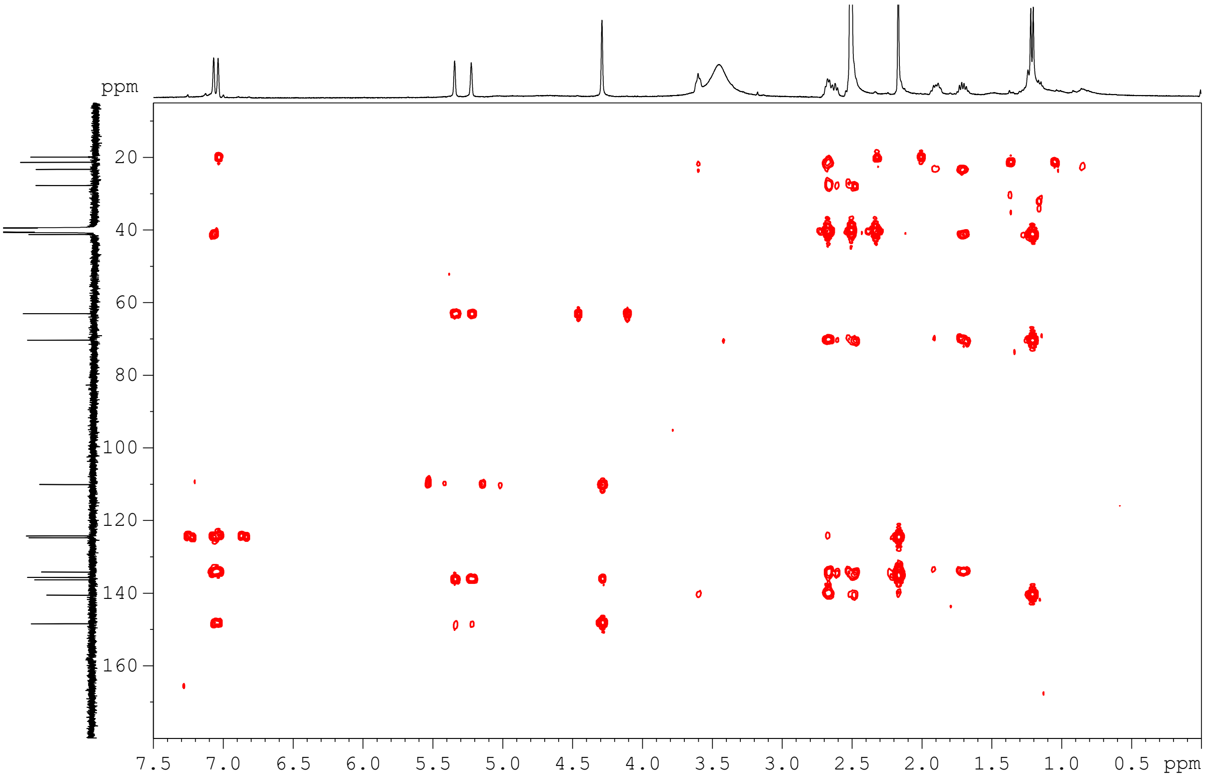

Figure S15. NOESY spectrum of **2** in DMSO-*d6*

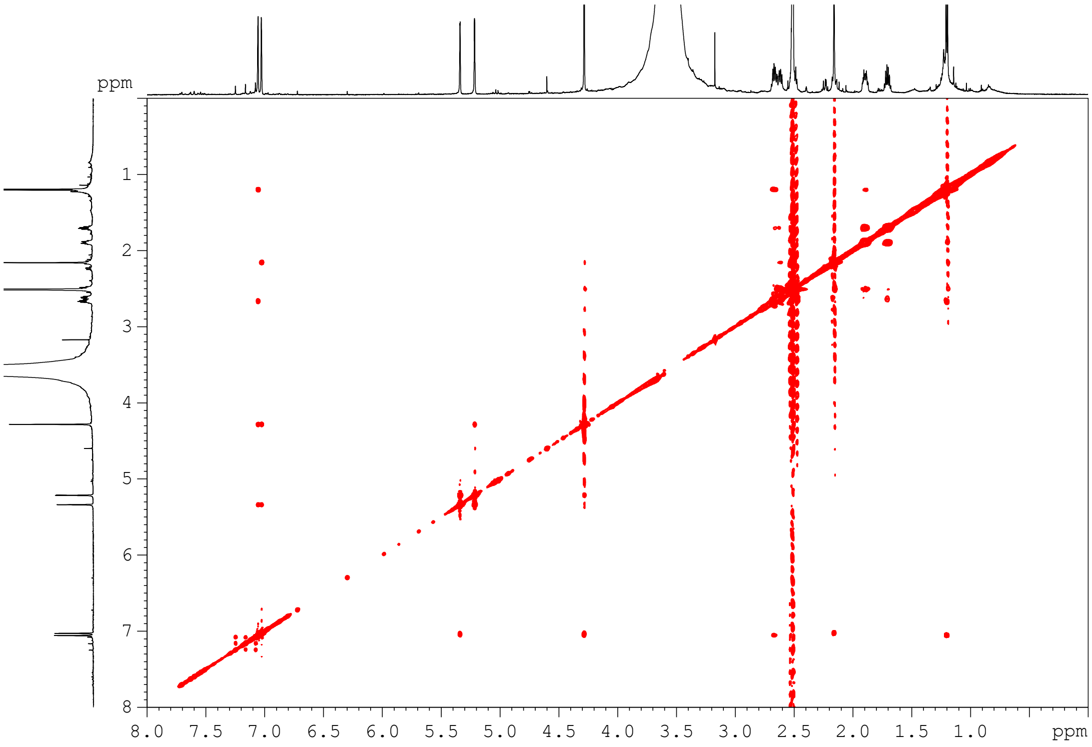

Figure S16. HRESIMS spectrum of **2**

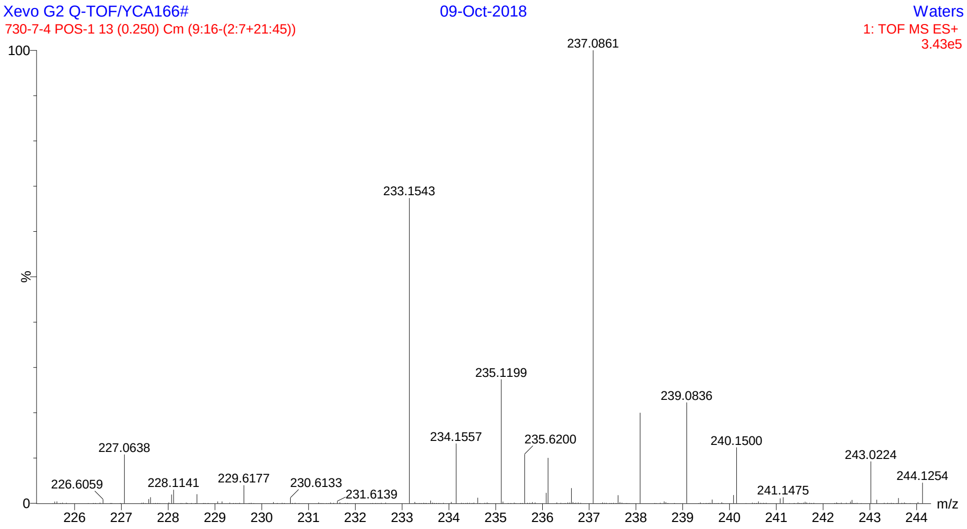

Mass	Calc. Mass	mDa	PPM	DBE	i-FIT	Norm	Conf(%)	Formula
233.1543	233.1542	0.1	0.4	5.5	430.1	n/a	n/a	$C_{15}H_{21}O_2$

Figure S17. IR spectrum of **3**

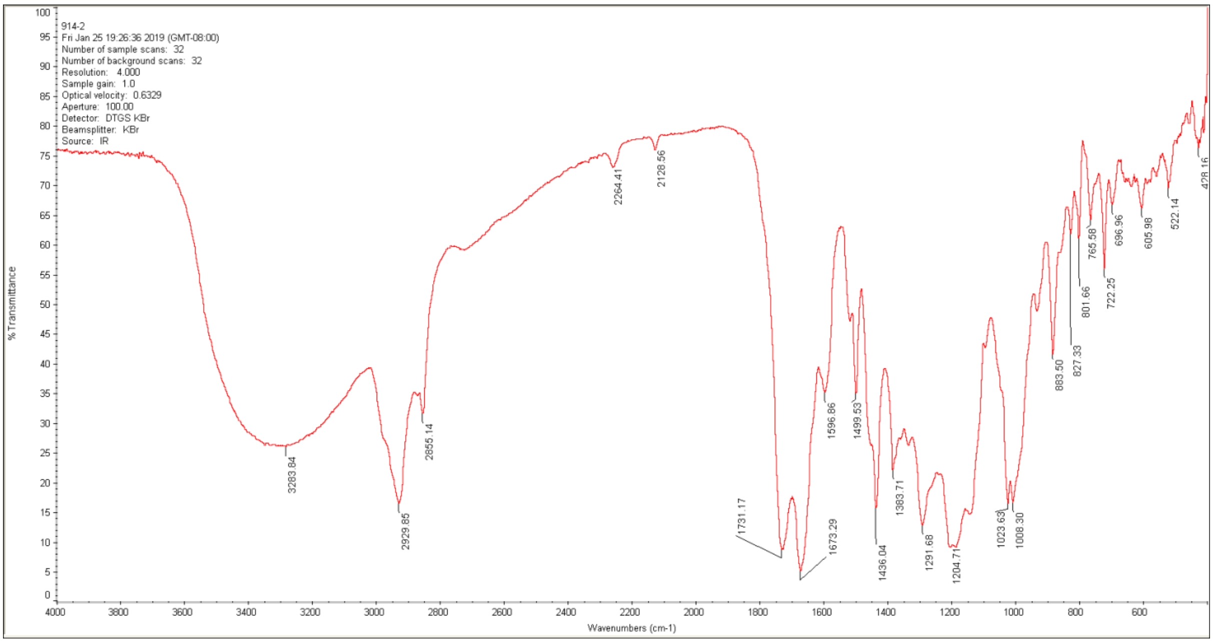

Figure S18. ^{1}H NMR (600 MHz) spectrum of **3** in DMSO-*d*6

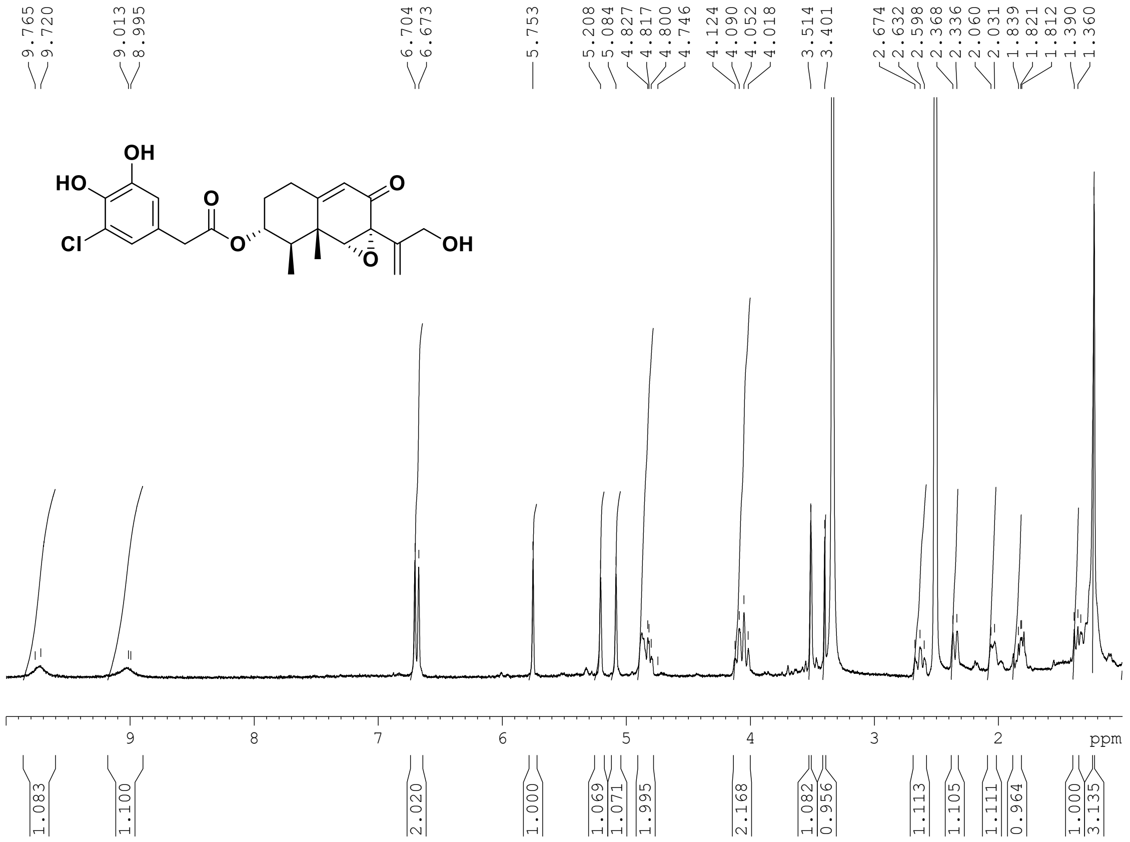

Figure S19. ^{13}C NMR (150 MHz) spectrum of **3** in DMSO-d_6

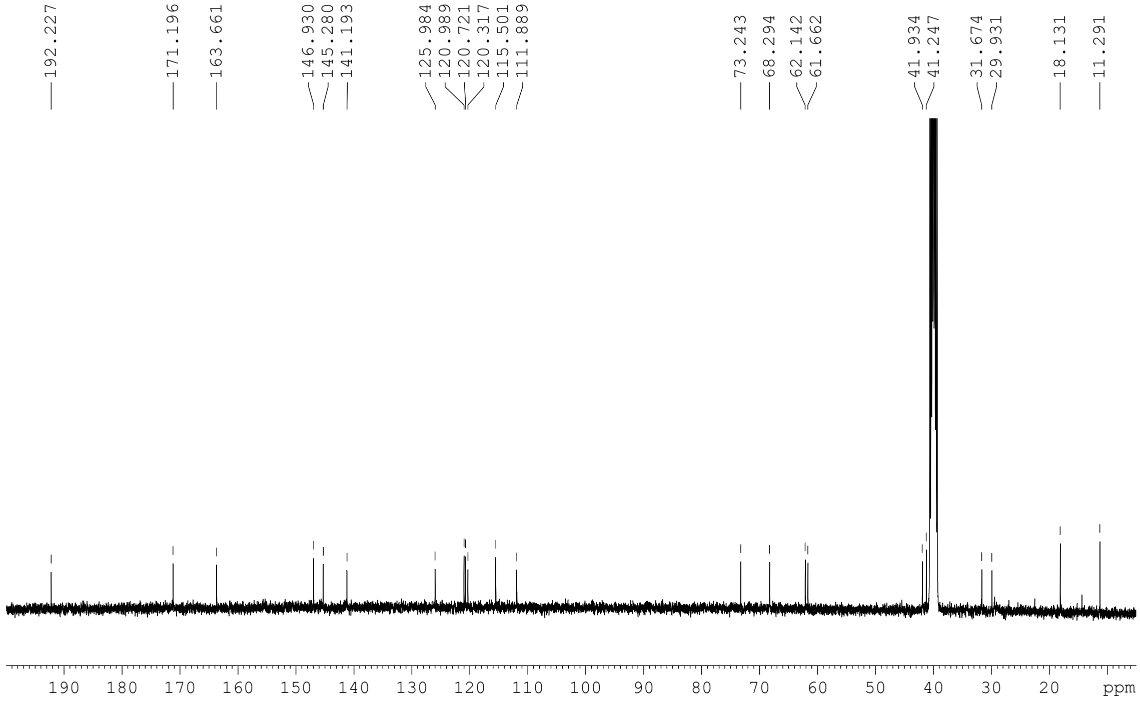

Figure S20. DEPT spectrum of **3** in DMSO-d_6

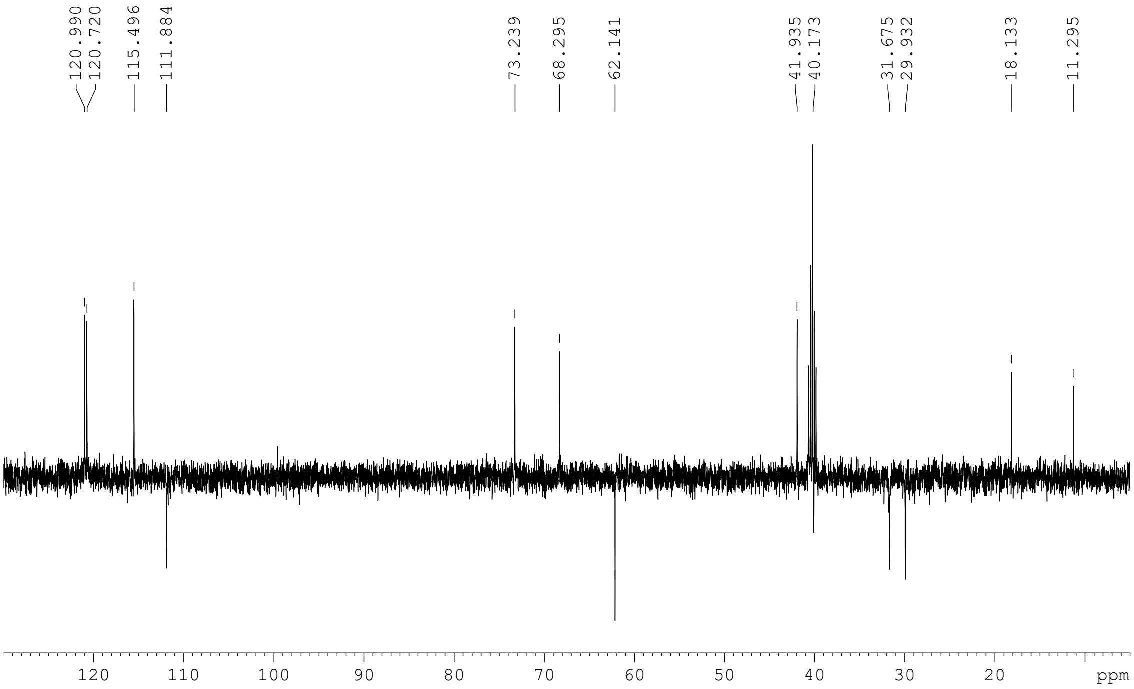

Figure S21. ^{1}H-^{1}H COSY spectrum of **3** in DMSO-$d6$

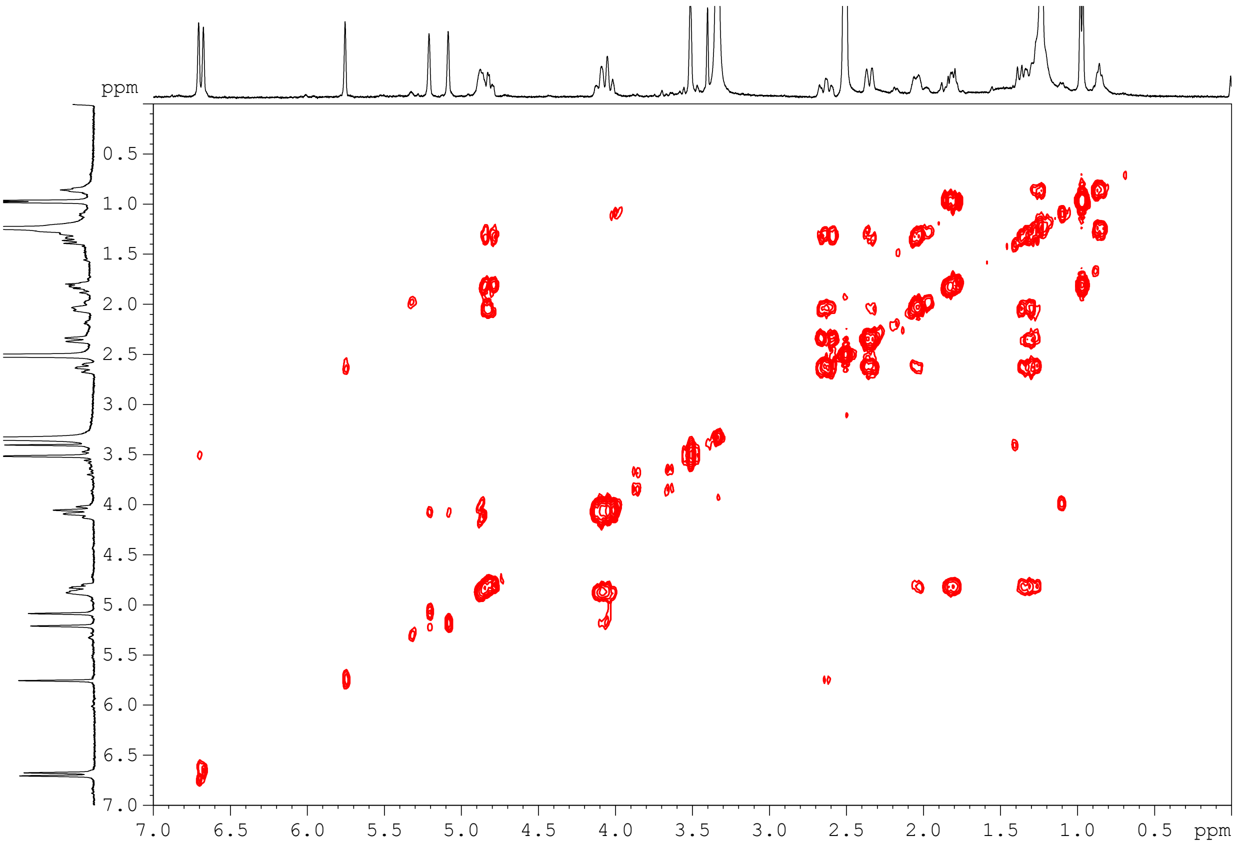

Figure S22. HSQC spectrum of **3** in DMSO-*d*6

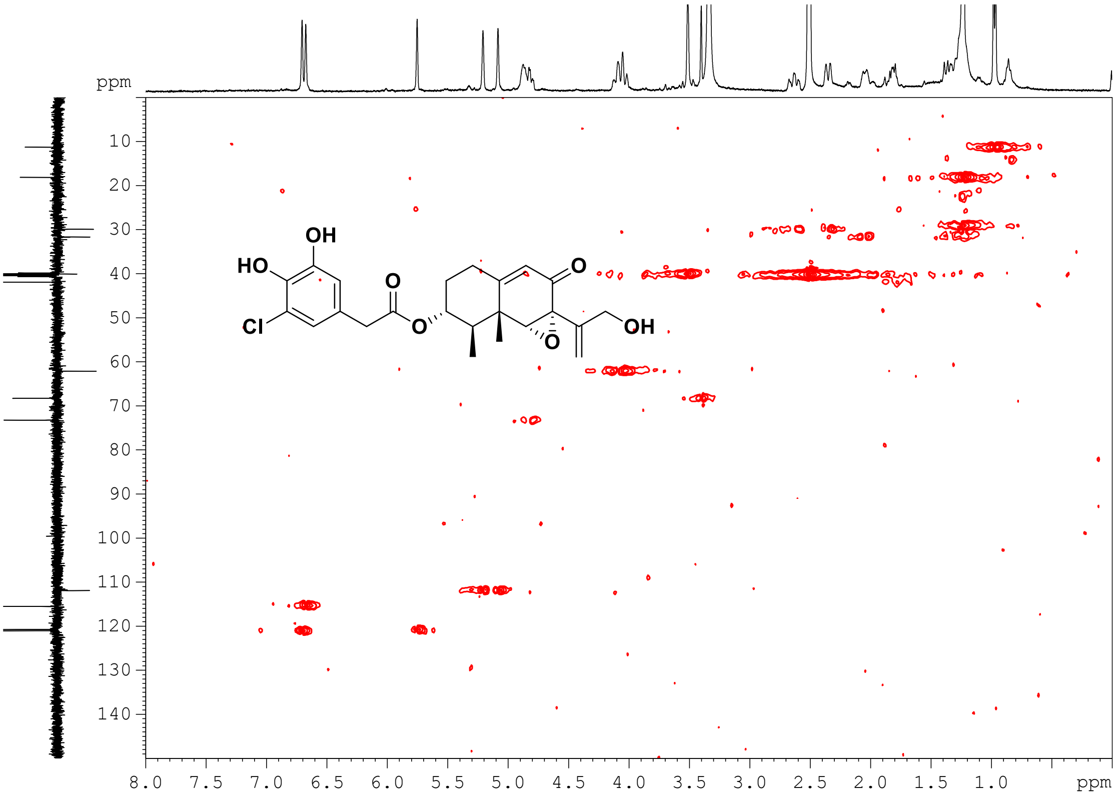

Figure S23. HMBC spectrum of **3** in DMSO-*d*6

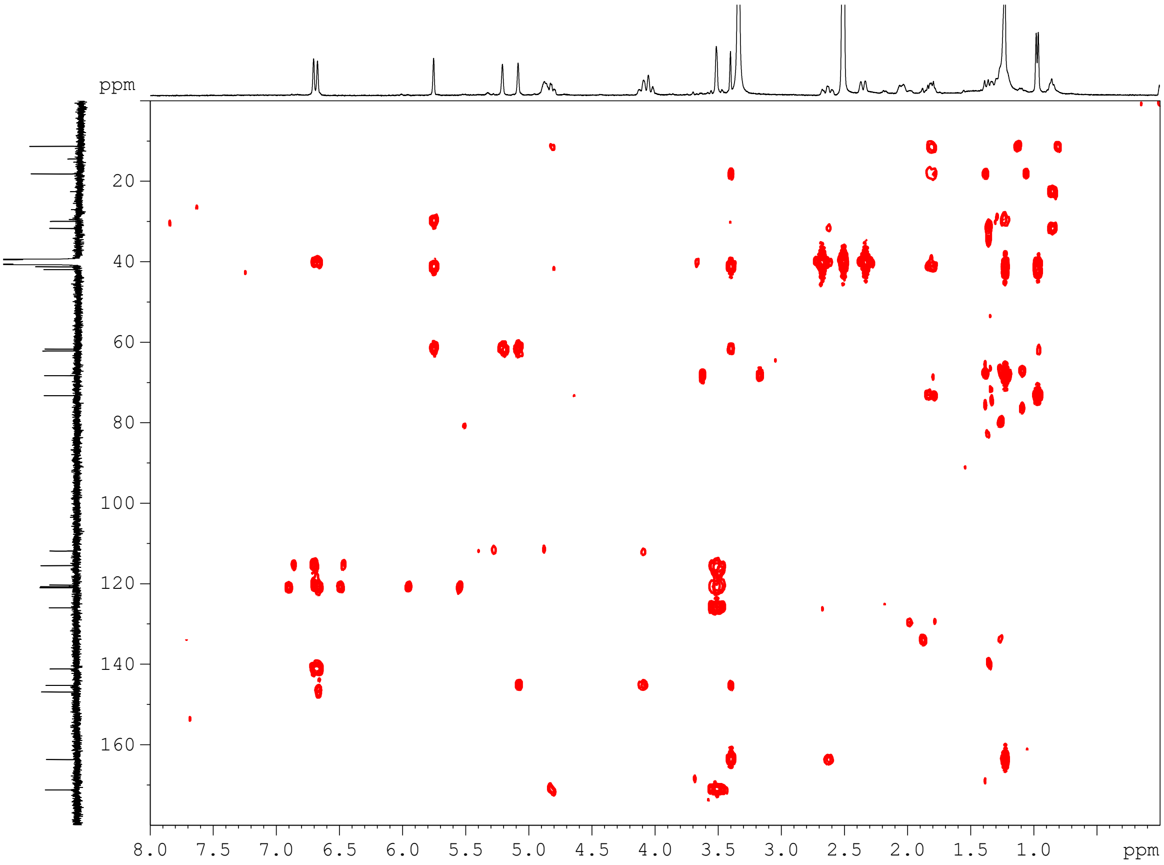

Figure S24. NOESY spectrum of **3** in DMSO-*d6*

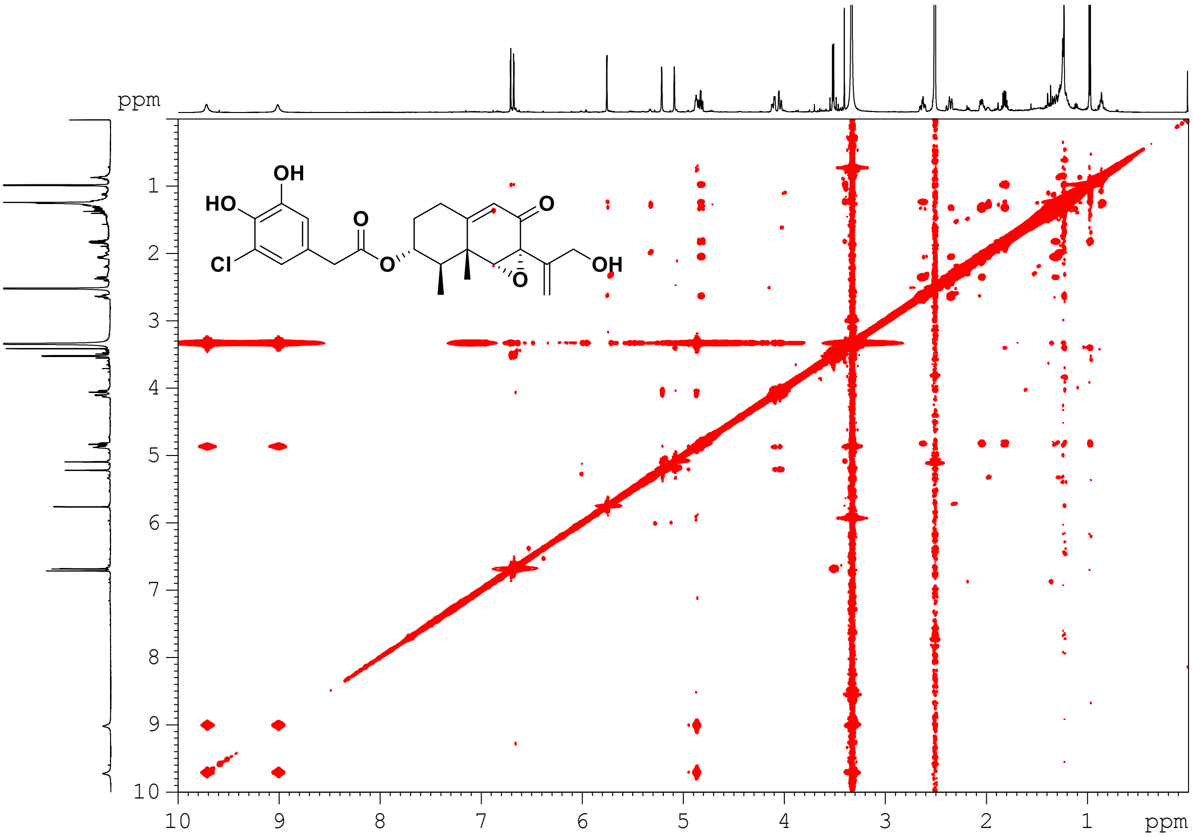

Figure S25. HRESIMS spectrum of **3**

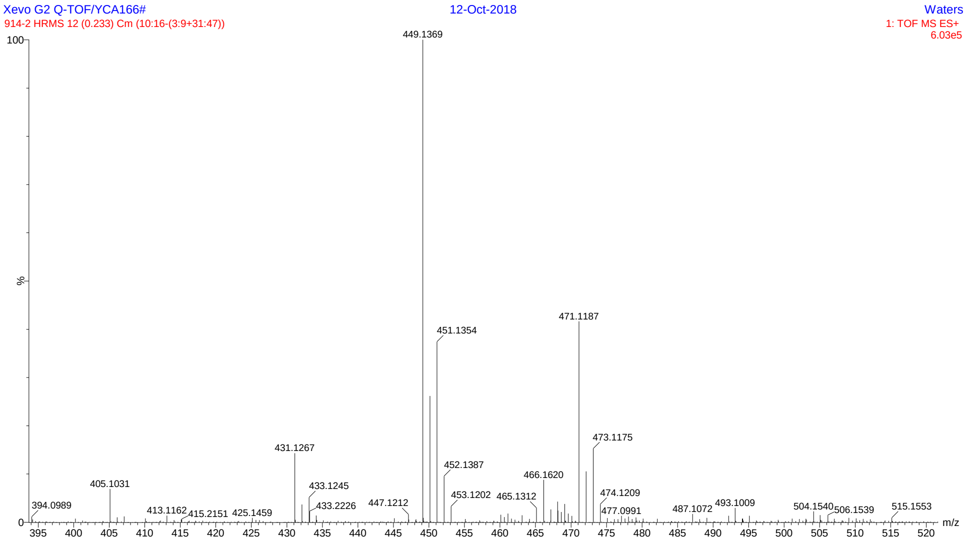

Mass	Calc. Mass	mDa	PPM	DBE	i-FIT	Norm	Conf(%)	Formula
449.1369	449.1367	0.2	0.4	2.5	278.2	0.023	97.68	$C_{23}H_{26}O_7Cl$

Figure S26. IR spectrum of **4**

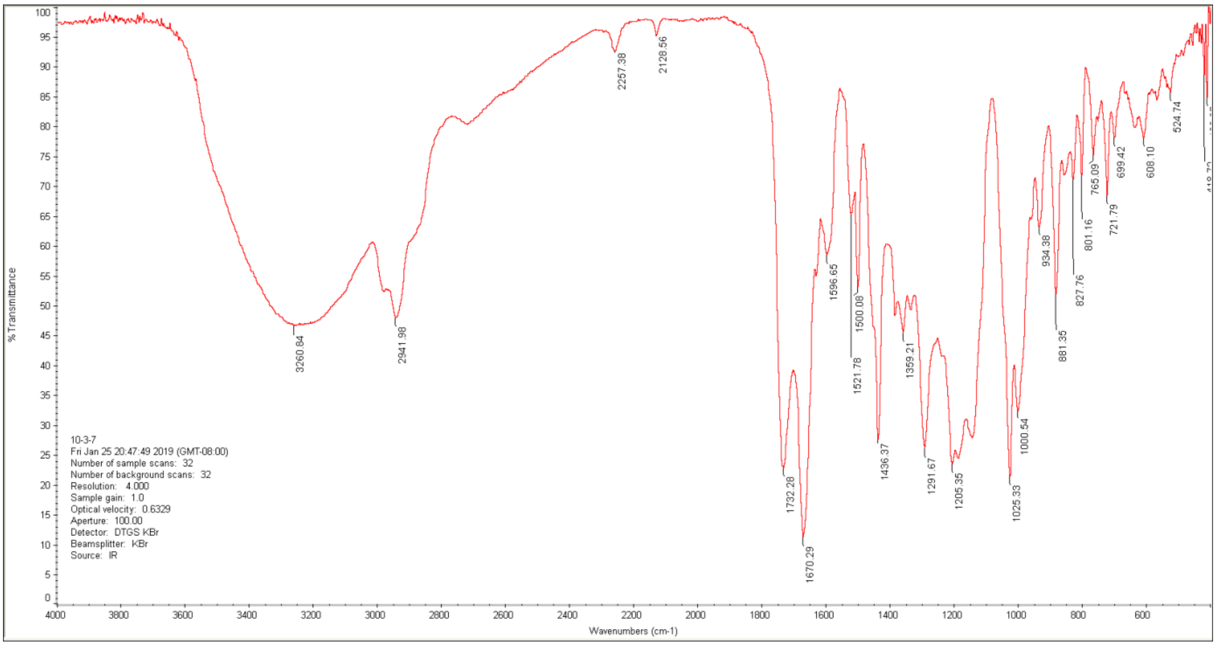

Figure S27. ^{1}H NMR (600 MHz) spectrum of **4** in DMSO-*d*6

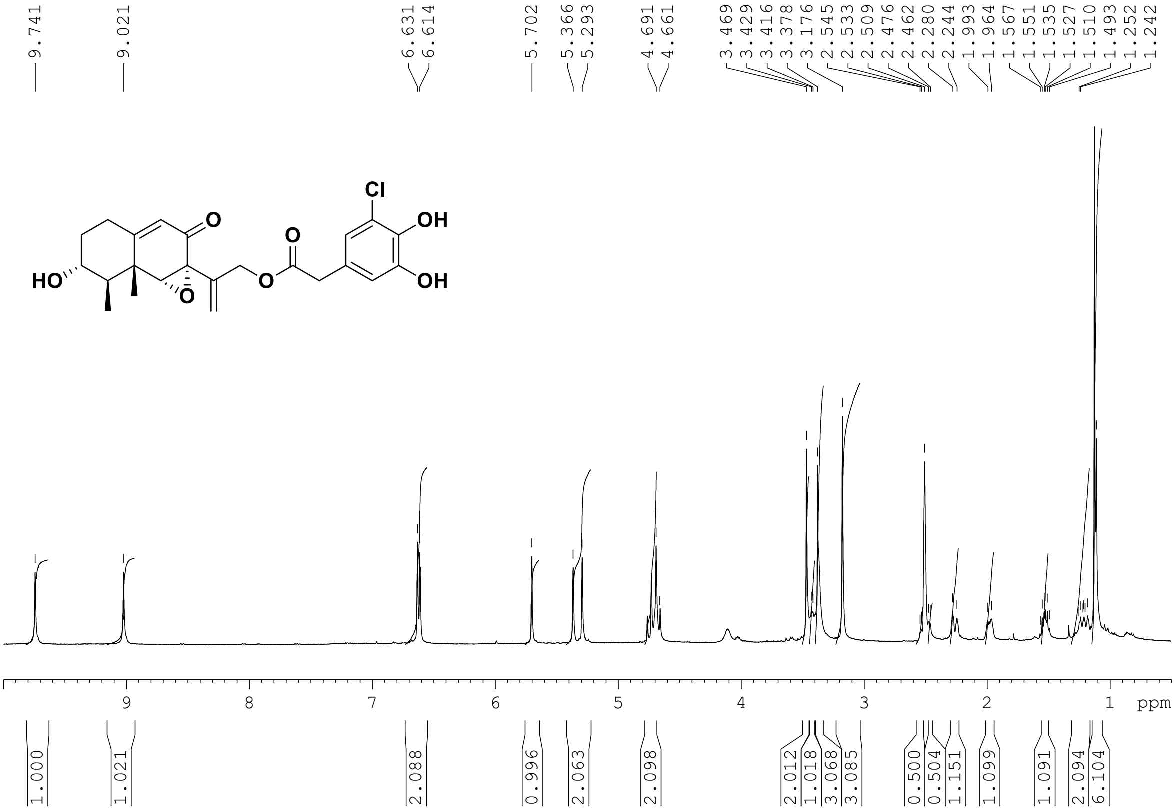

Figure S28. ^{13}C NMR (150 MHz) and DEPT spectra of **4** in DMSO-d_6

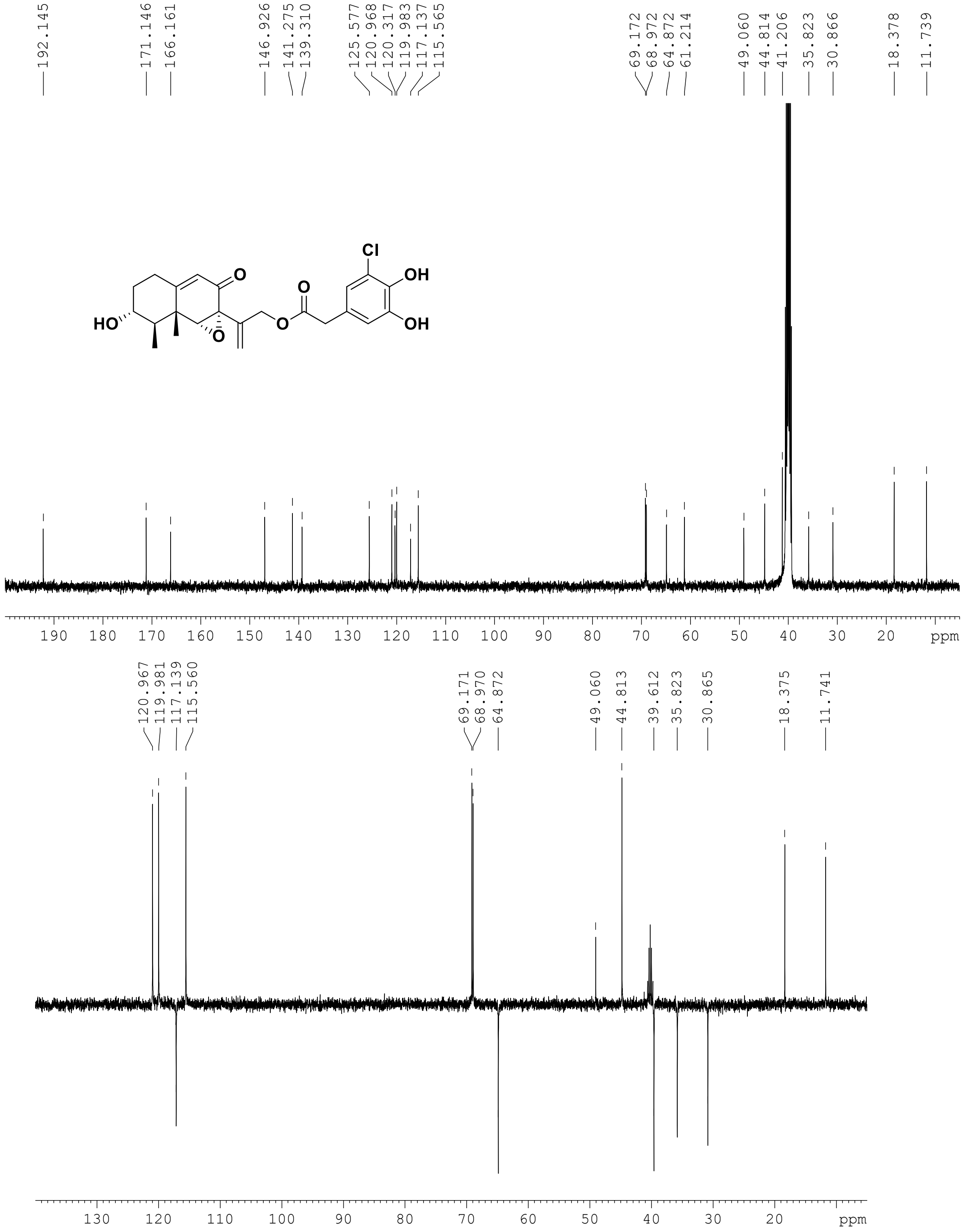

Figure S29. ^{1}H-^{1}H COSY spectrum of **4** in DMSO-*d6*

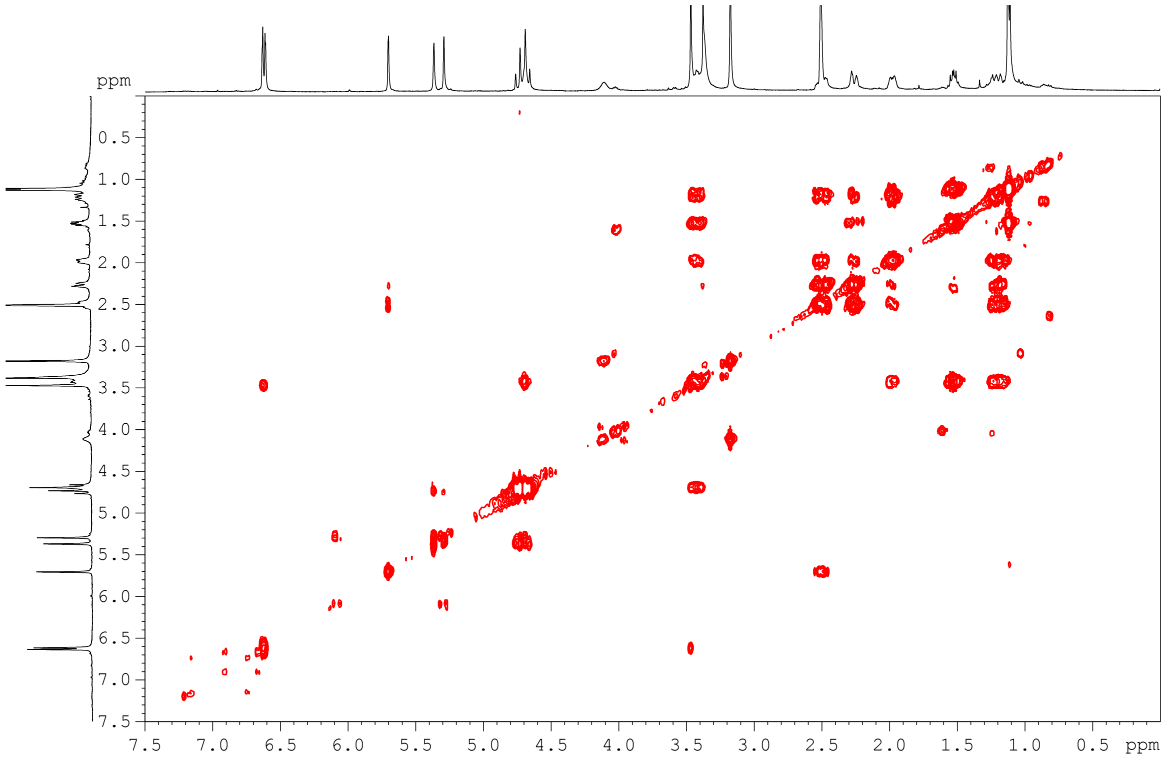

Figure S30. HSQC spectrum of **4** in DMSO-*d6*

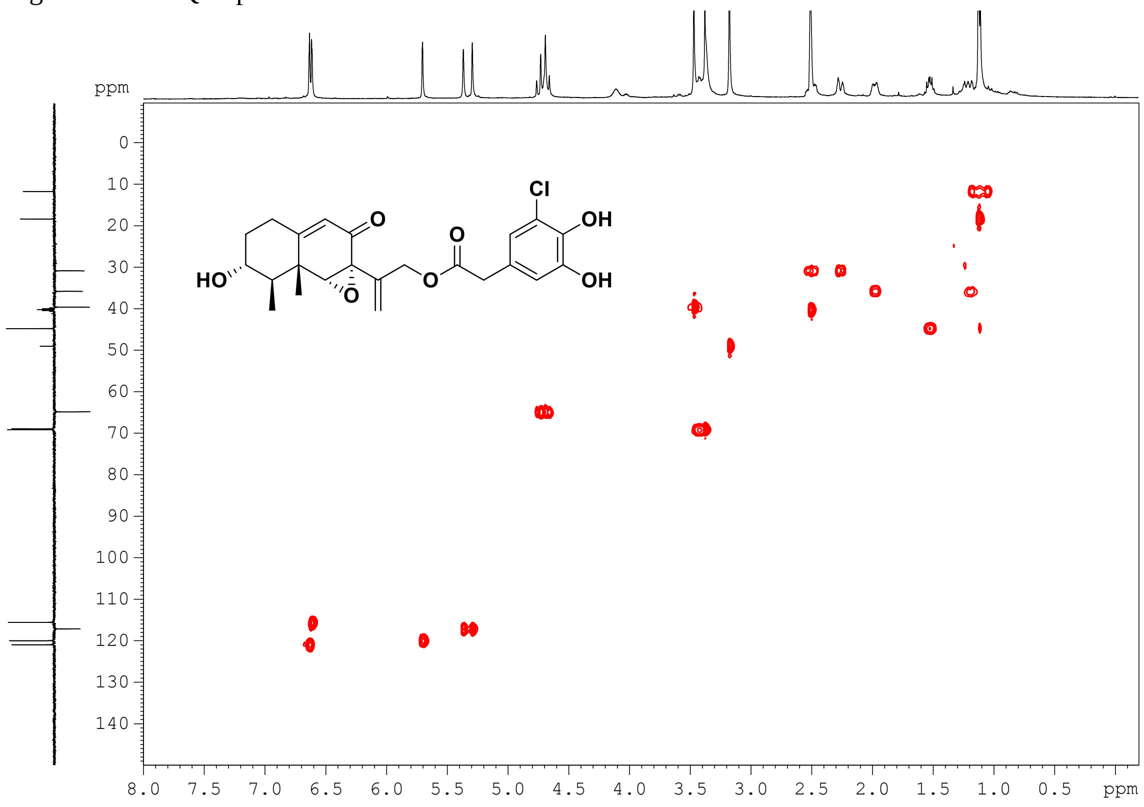

Figure S31. HMBC spectrum of **4** in DMSO-*d6*

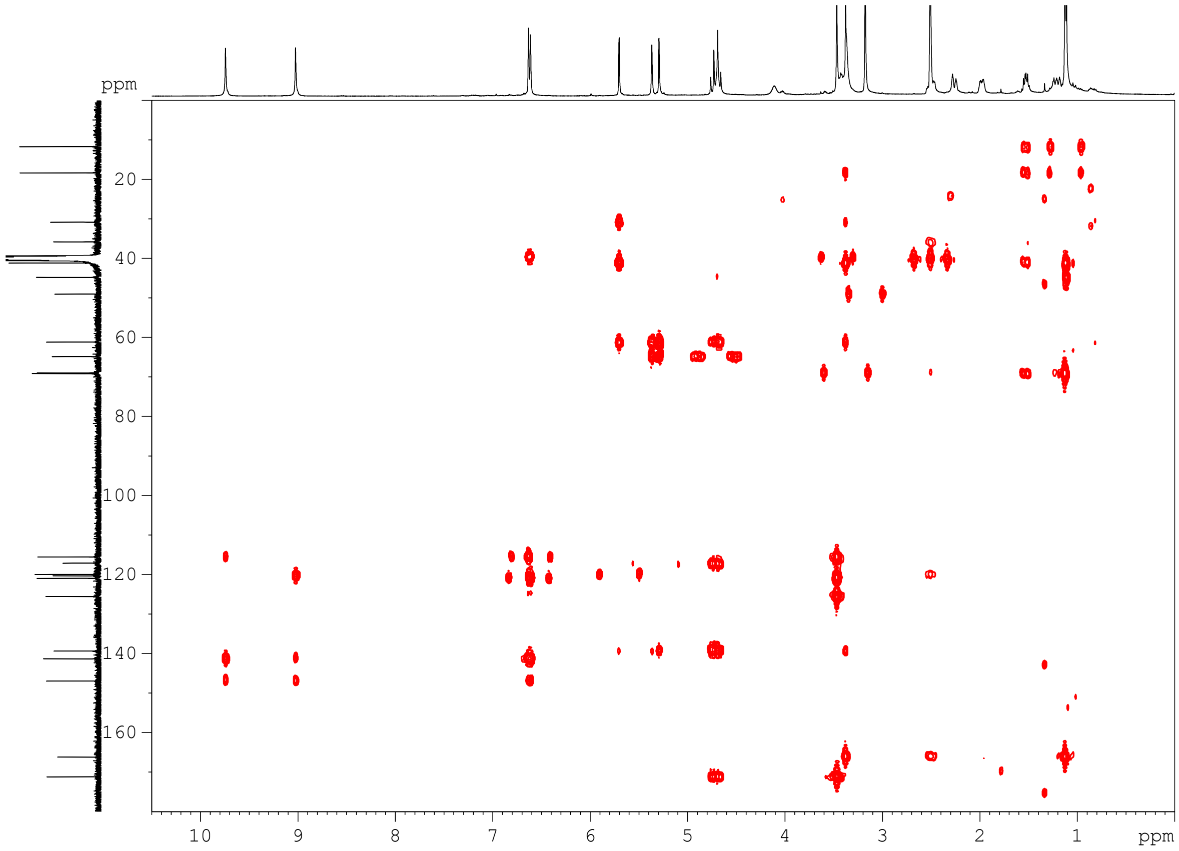

Figure S32. NOESY spectrum of **4** in DMSO-*d6*

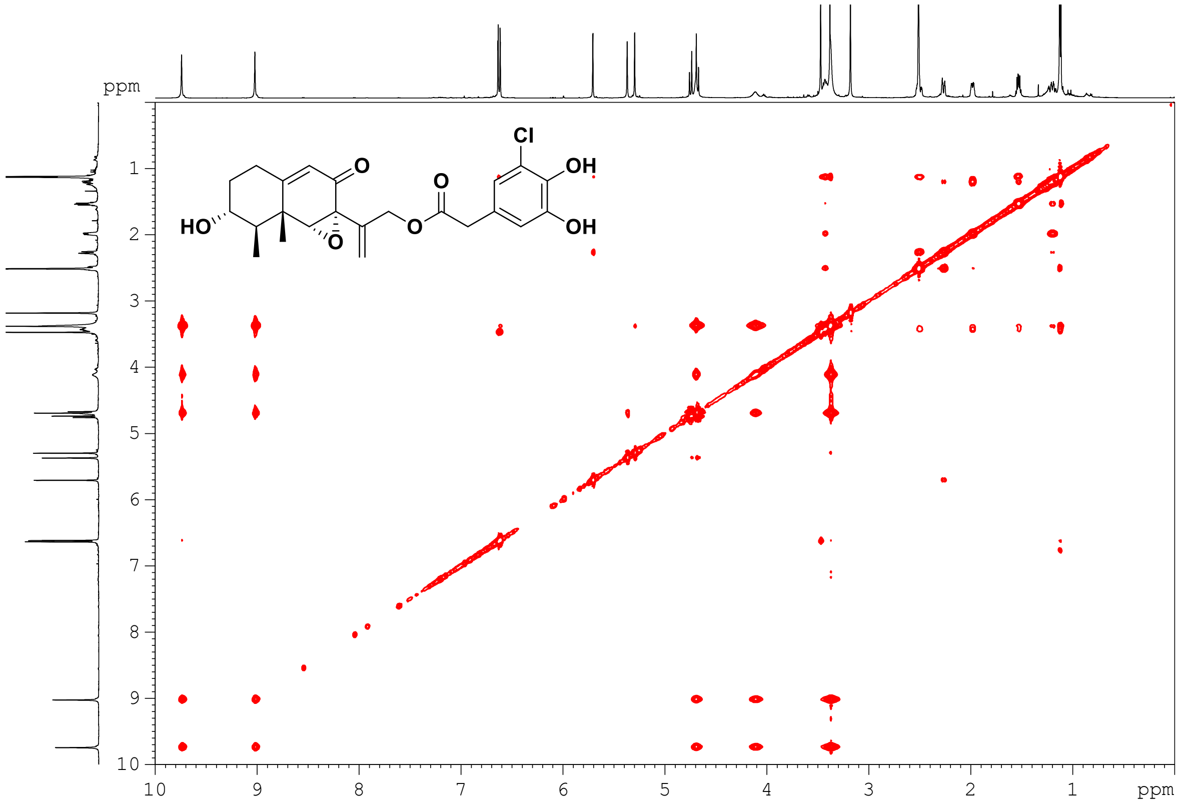

Figure S33. HRESIMS spectrum of **4**

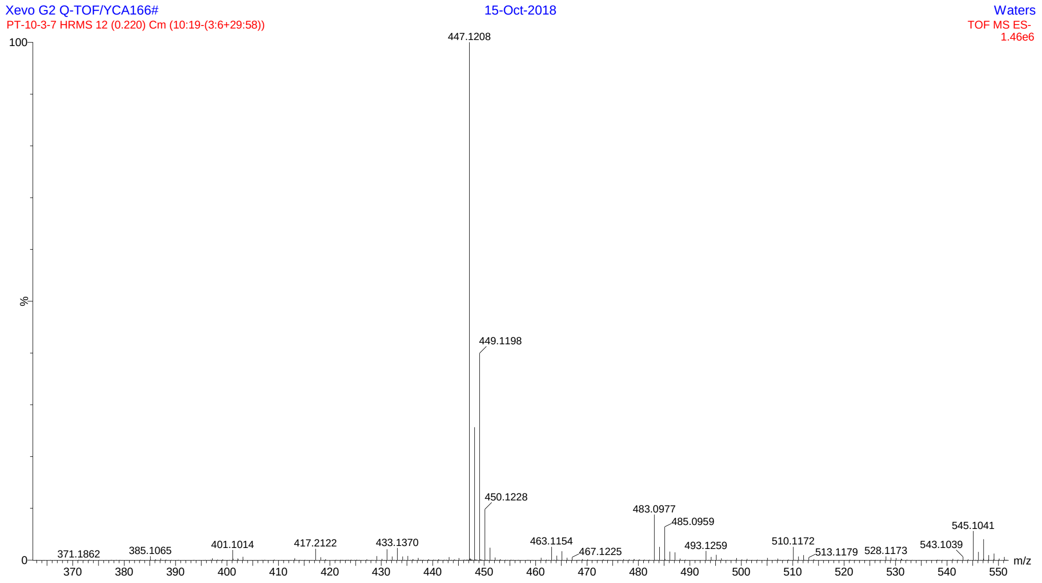

Mass	Calc. Mass	mDa	PPM	DBE	i-FIT	Norm	Conf(%)	Formula
447.1208	447.1211	-0.3	-0.7	11.5	419.8	0.000	100.00	$C_{23}H_{24}O_7Cl$

Figure S34. IR spectrum of **5**

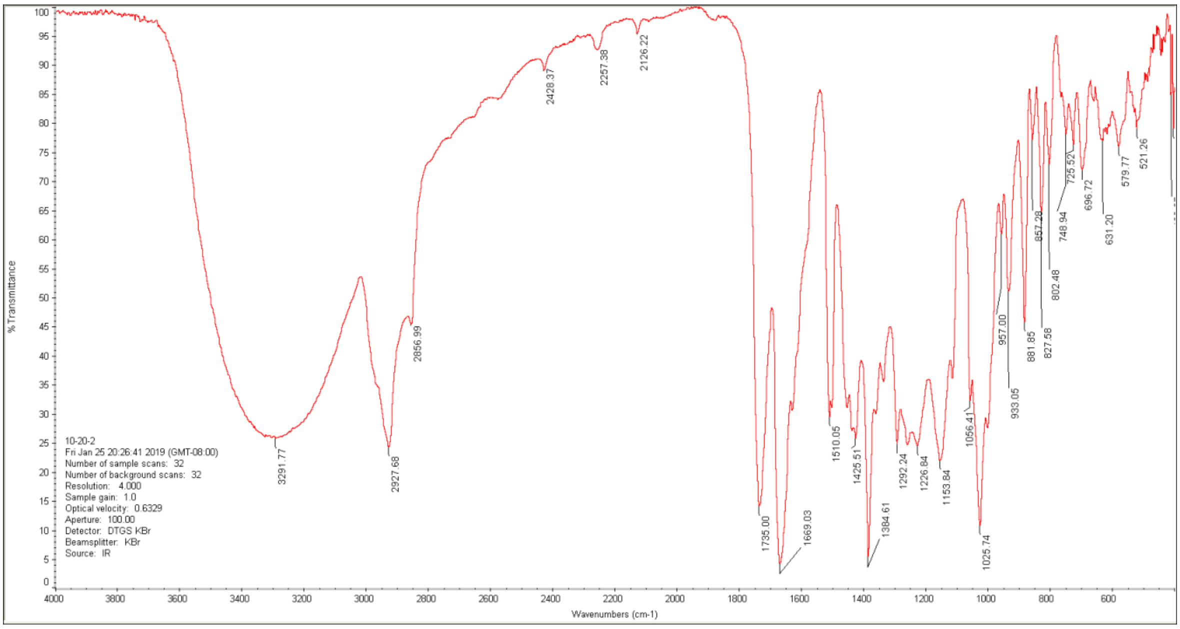

Figure S35. ^{1}H NMR (600 MHz) spectrum of **5** in DMSO-*d*6

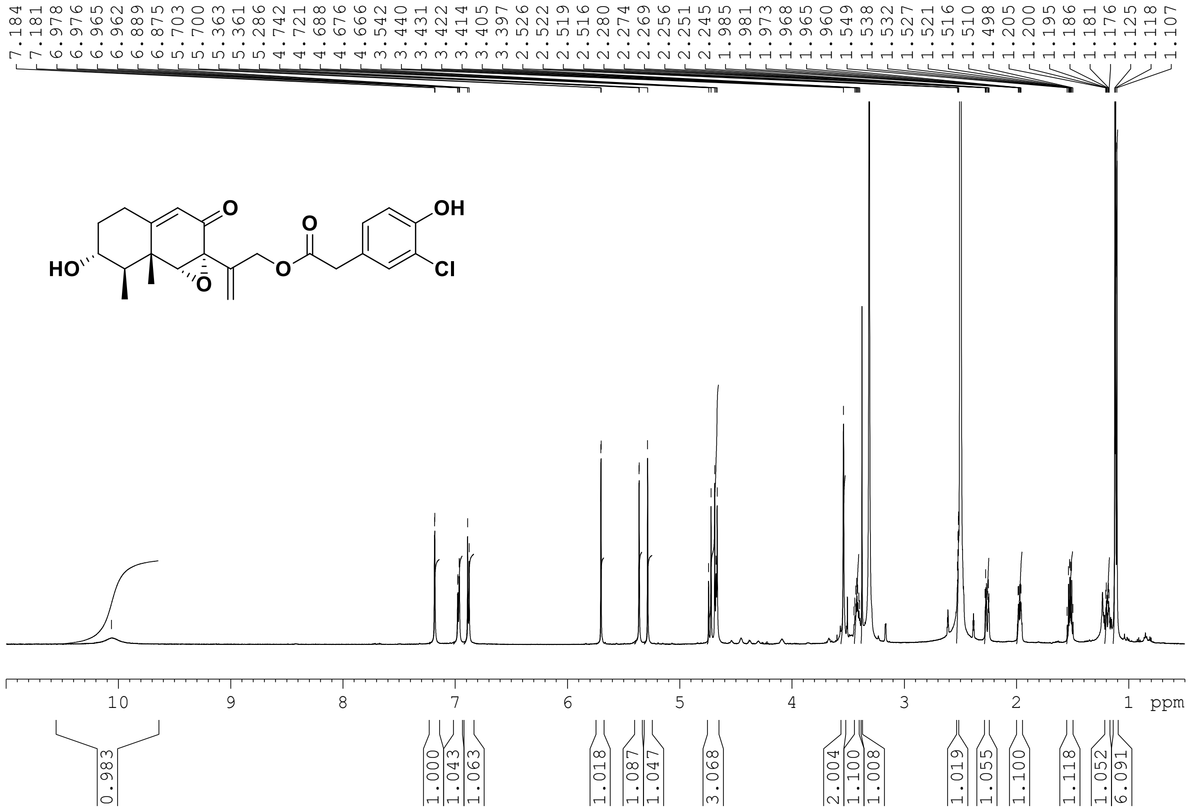

Figure S36. ^{13}C NMR (150 MHz) spectrum of **5** in DMSO-*d*6

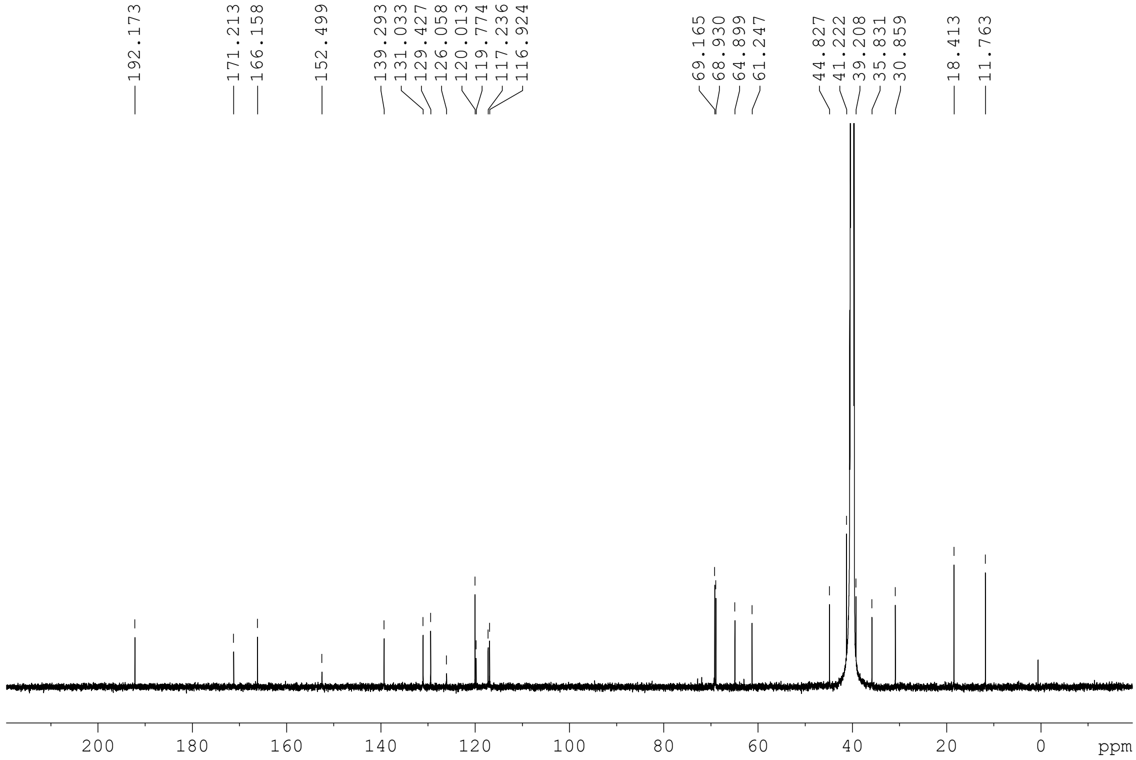

Figure S37. DEPT spectrum of **5** in DMSO-d_6

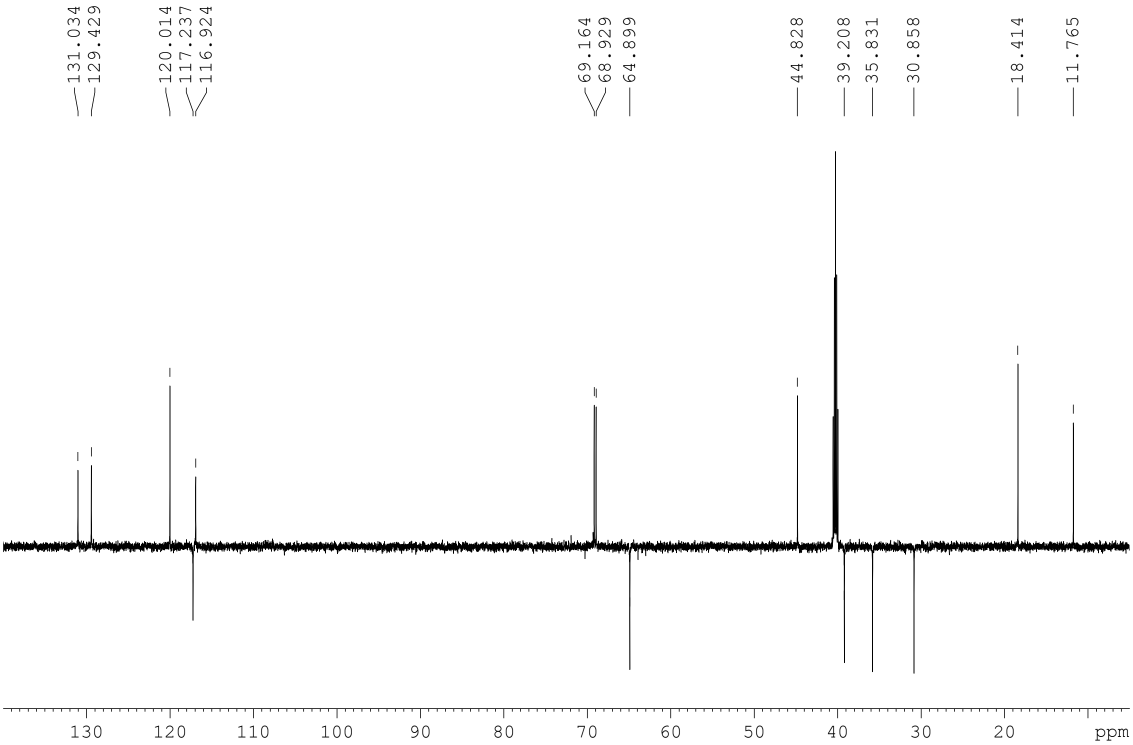

Figure S38. ^{1}H-^{1}H COSY spectrum of **5** in DMSO-$d6$

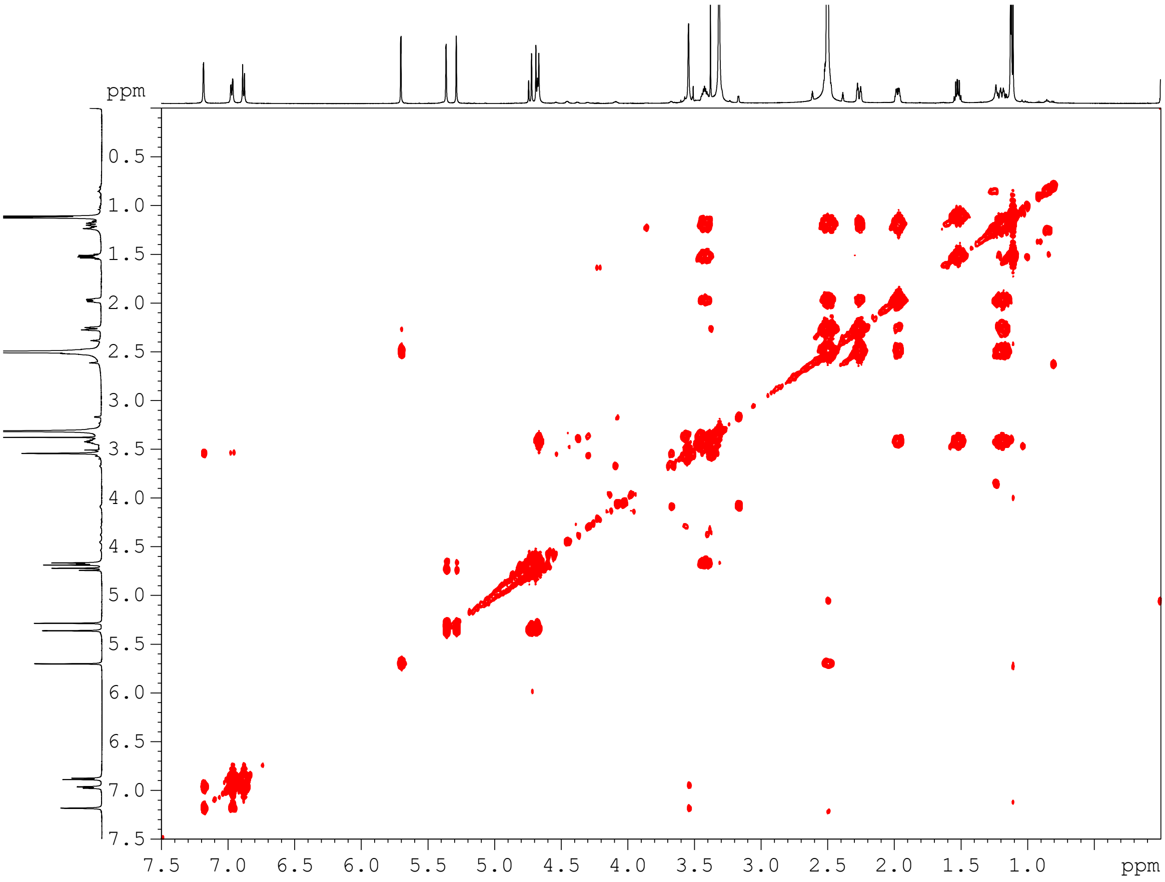

Figure S39. HSQC spectrum of **5** in DMSO-*d*6

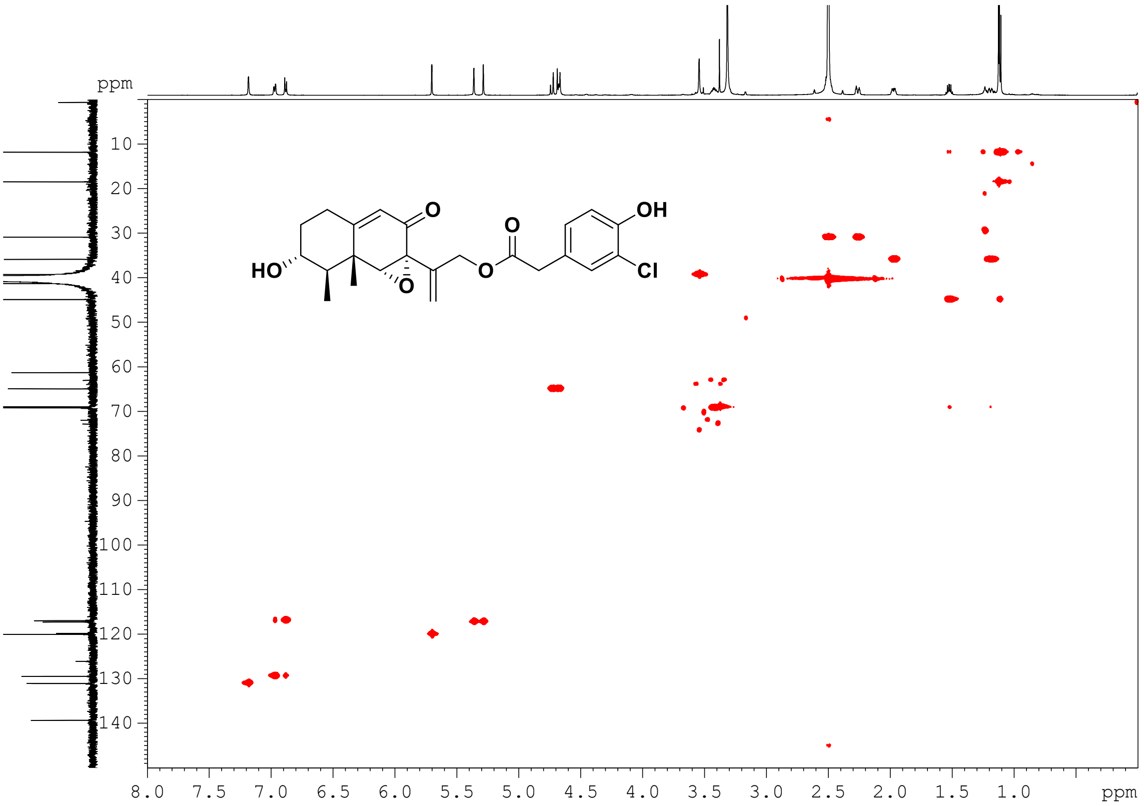

Figure S40. HMBC spectrum of **5** in DMSO-*d*6

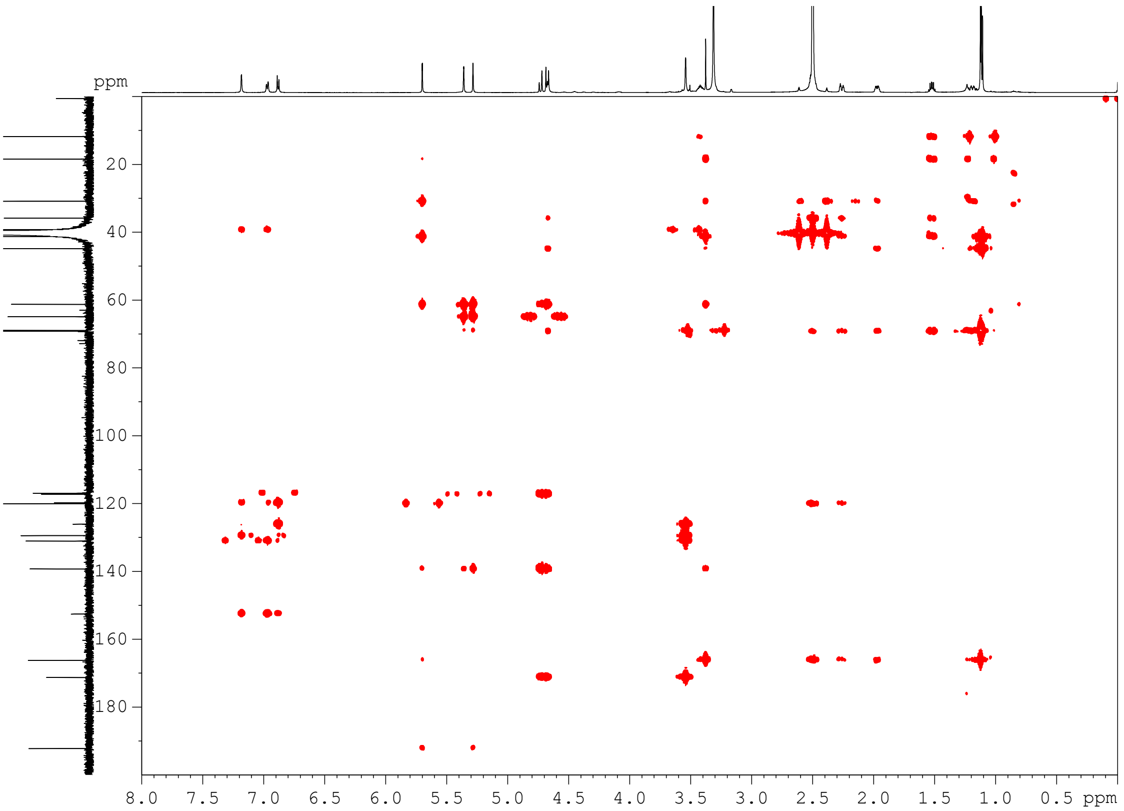

Figure S41. NOESY spectrum of **5** in DMSO-*d6*

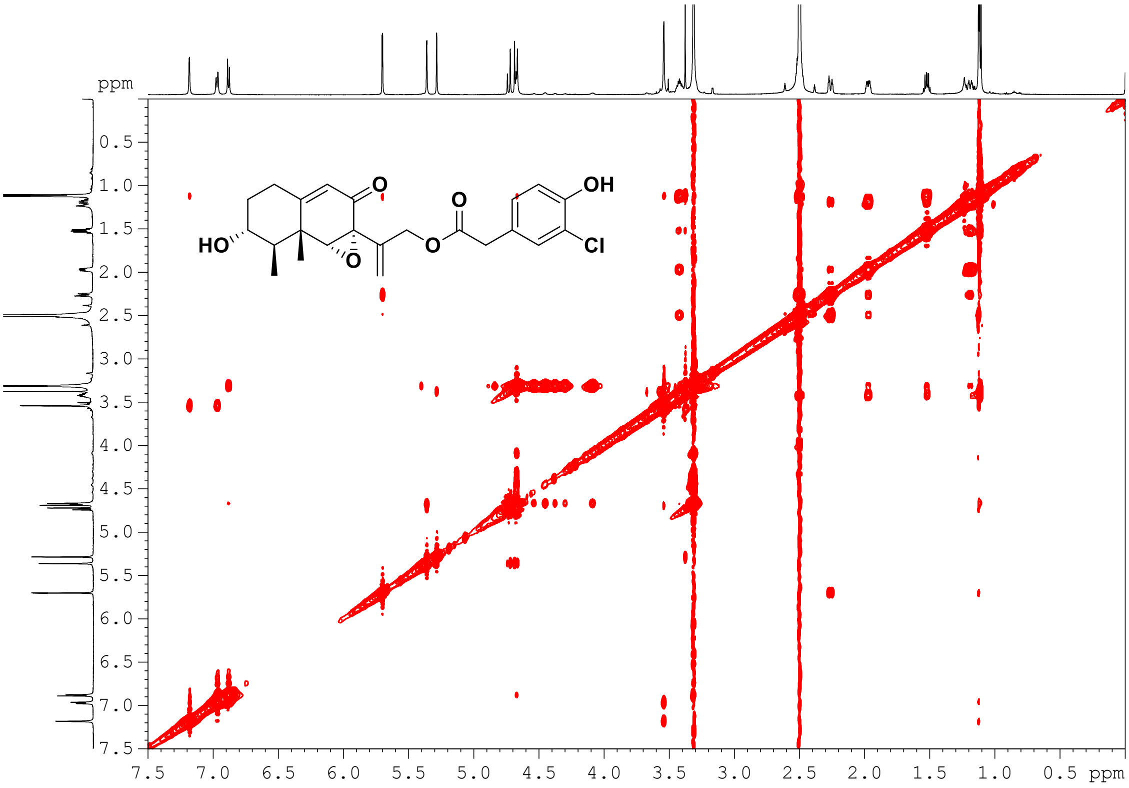

Figure S42. HRESIMS spectrum of **5**

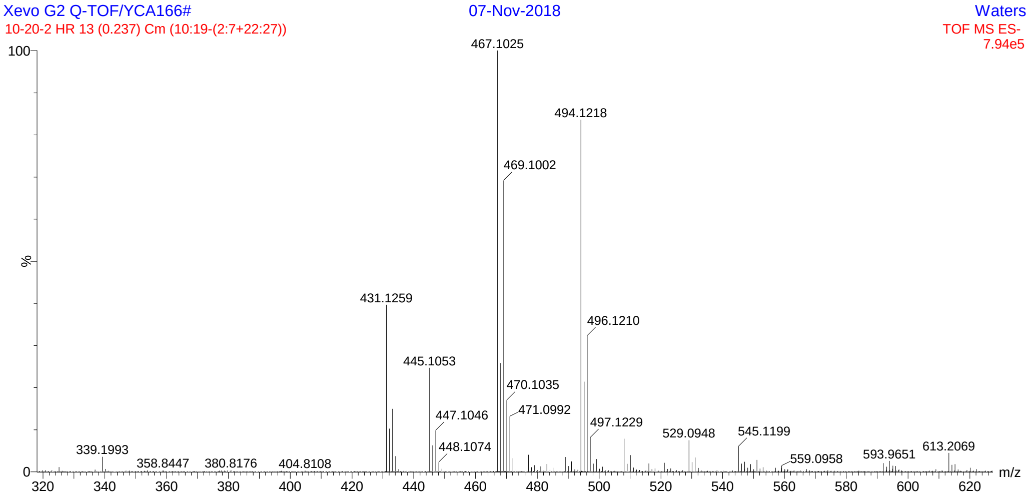

Mass	Calc. Mass	mDa	PPM	DBE	i-FIT	Norm	Conf(%)	Formula
431.1259	431.1261	-0.2	-0.5	11.5	369.5	0.000	100.00	$C_{23}H_{24}O_6Cl$

Figure S43. IR spectrum of **6**

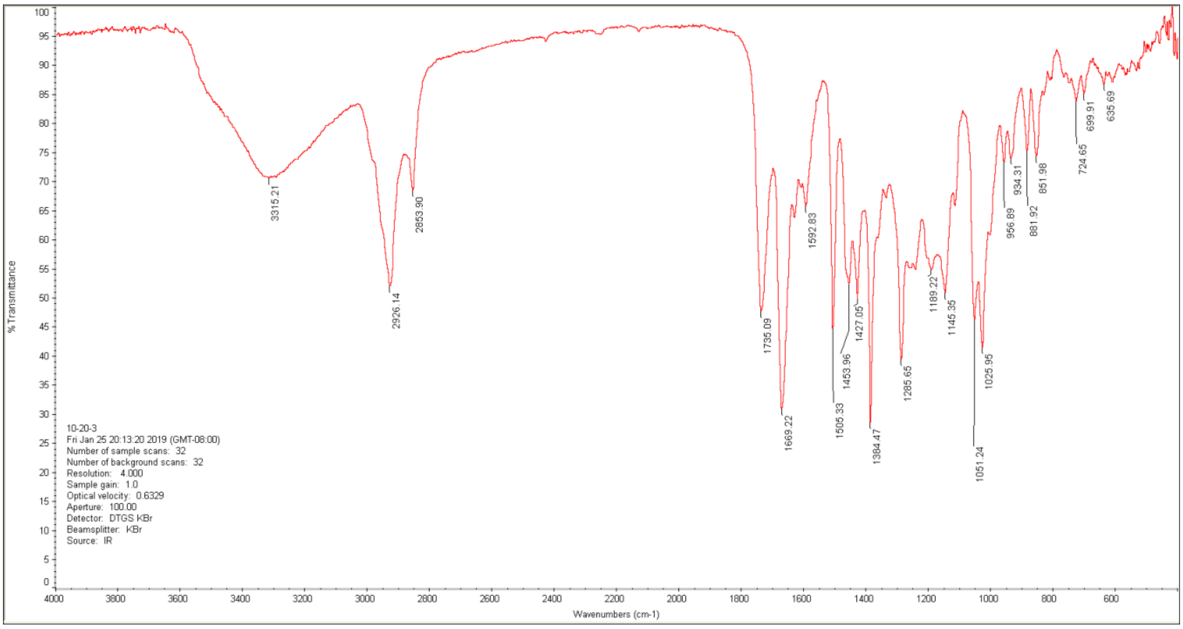

Figure S44. ^{1}H NMR (600 MHz) spectrum of **6** in DMSO-*d6*

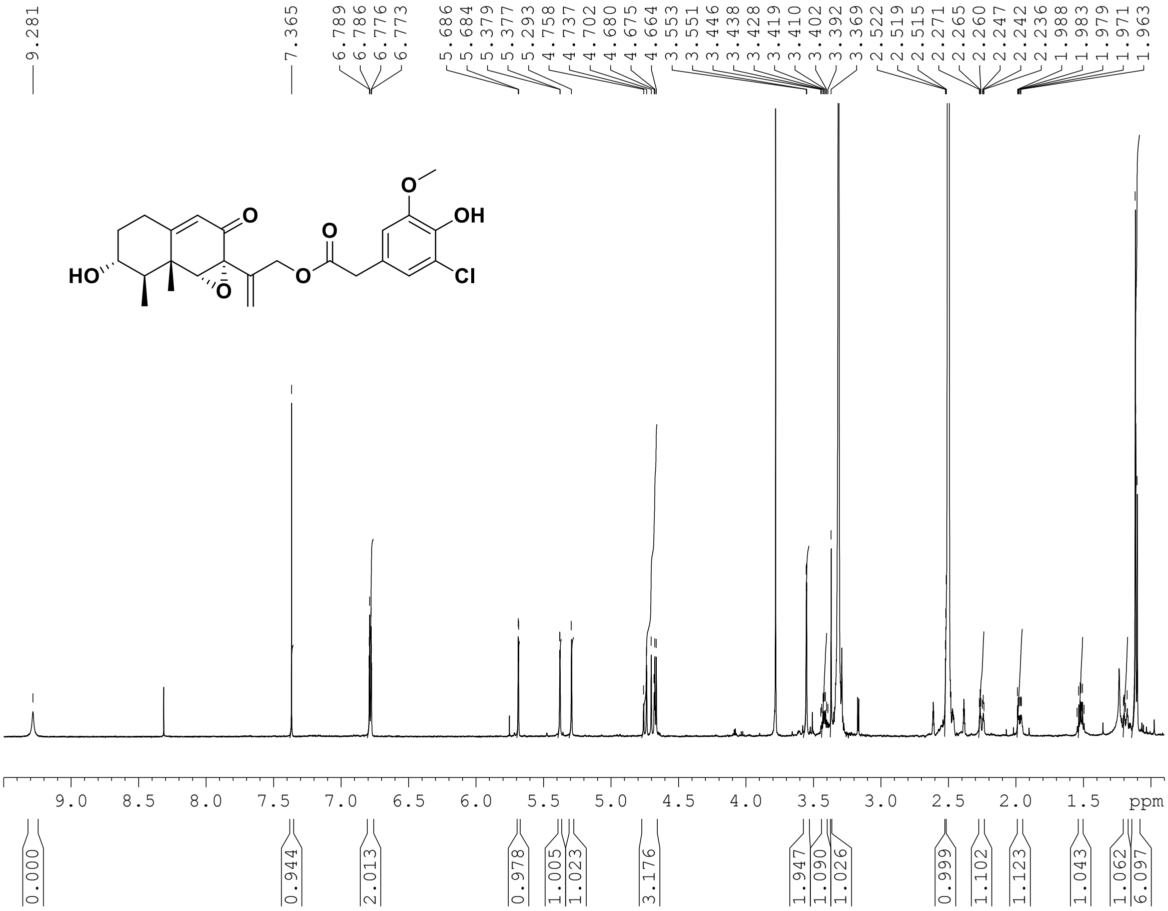

Figure S45. ^{13}C NMR (150 MHz) and DEPT spectra of **6** in DMSO-d_6

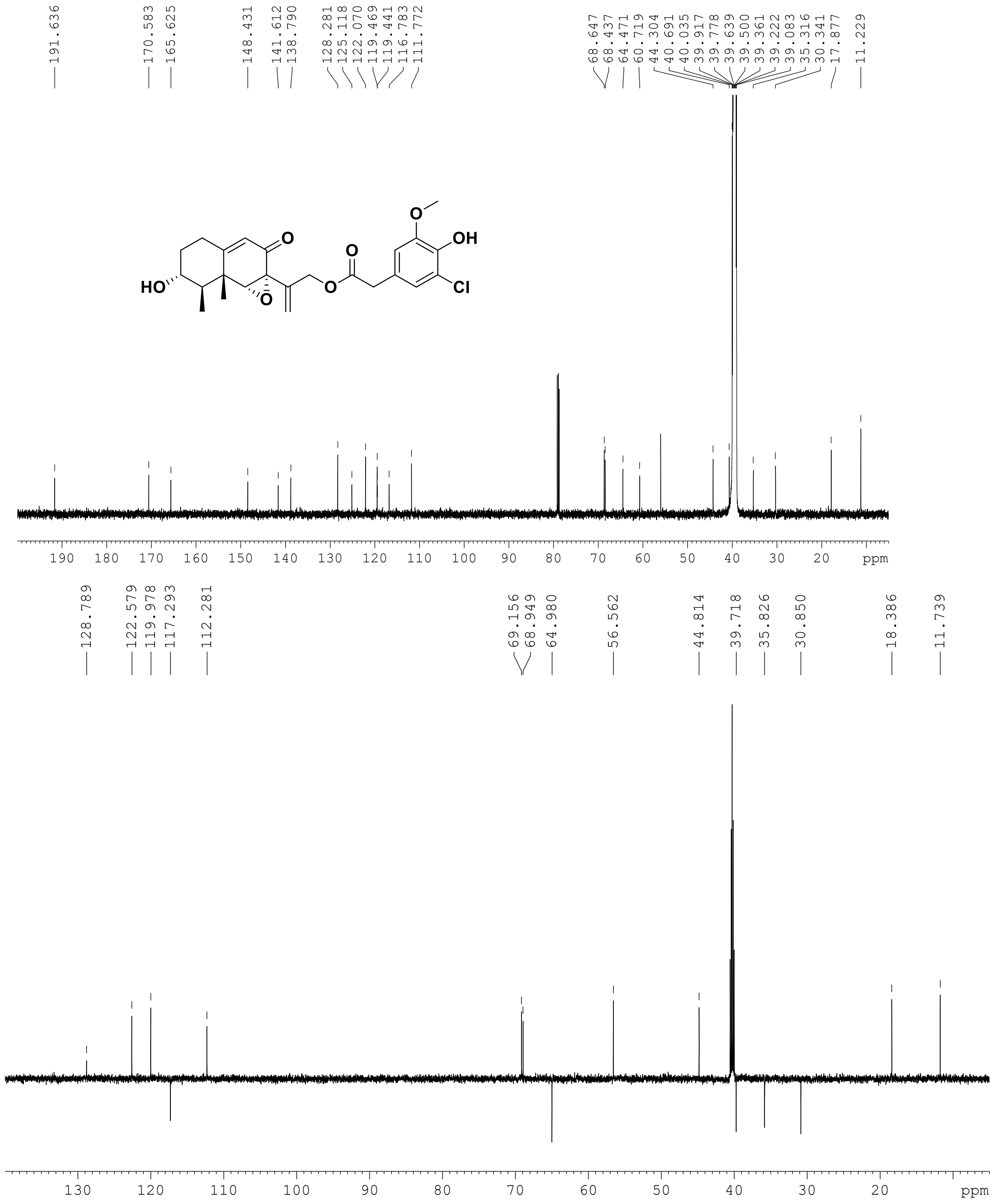

Figure S46. ^{1}H-^{1}H COSY spectrum of **6** in DMSO-*d6*

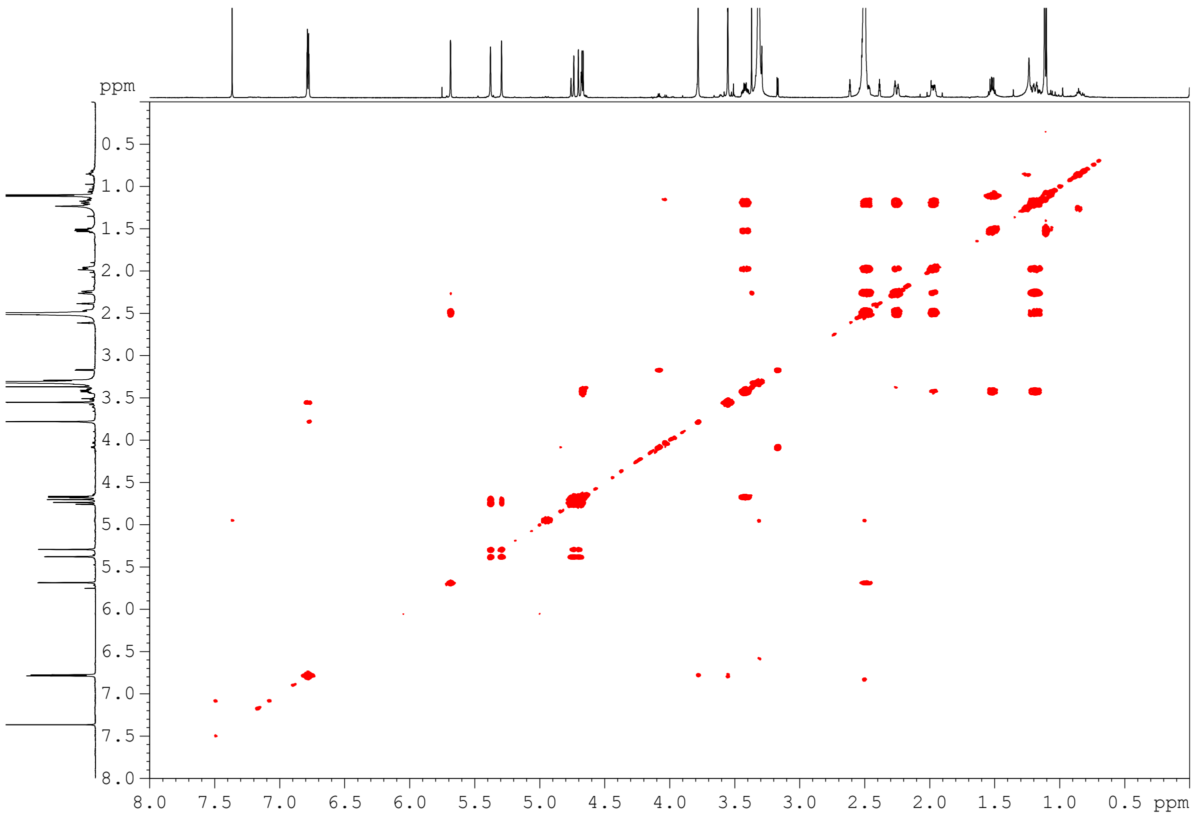

Figure S47. HSQC spectrum of **6** in DMSO-*d6*

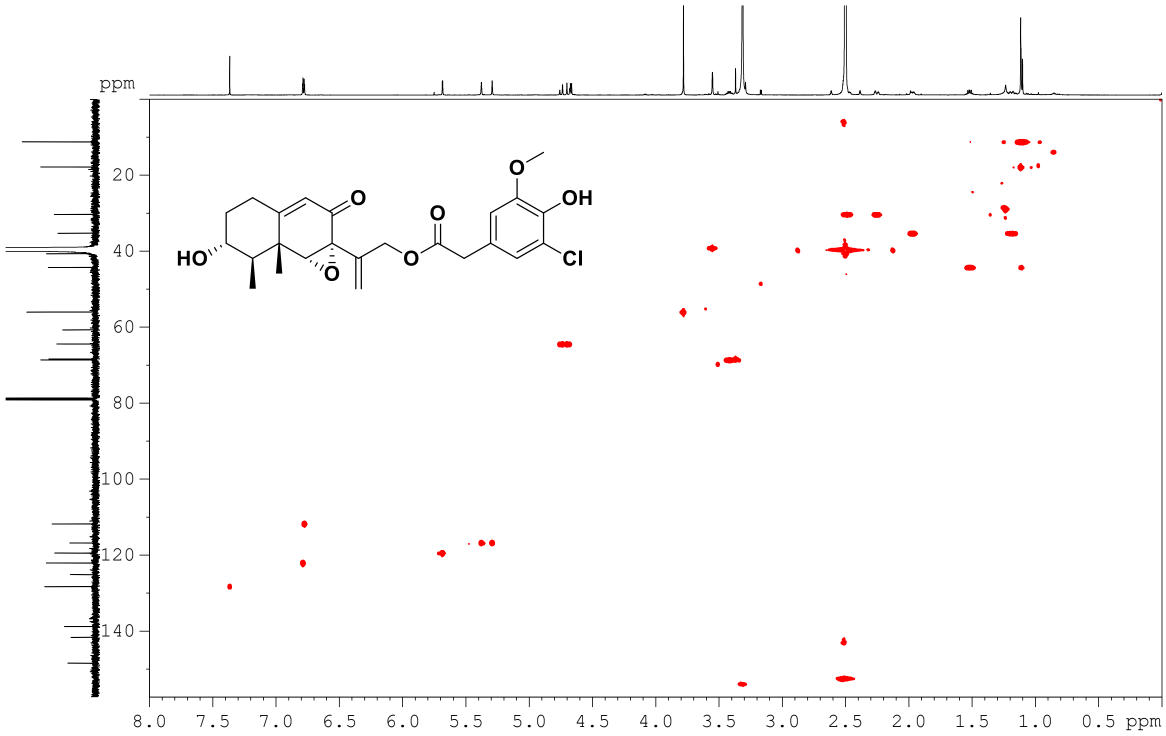

Figure S48. HMBC spectrum of **6** in DMSO-*d6*

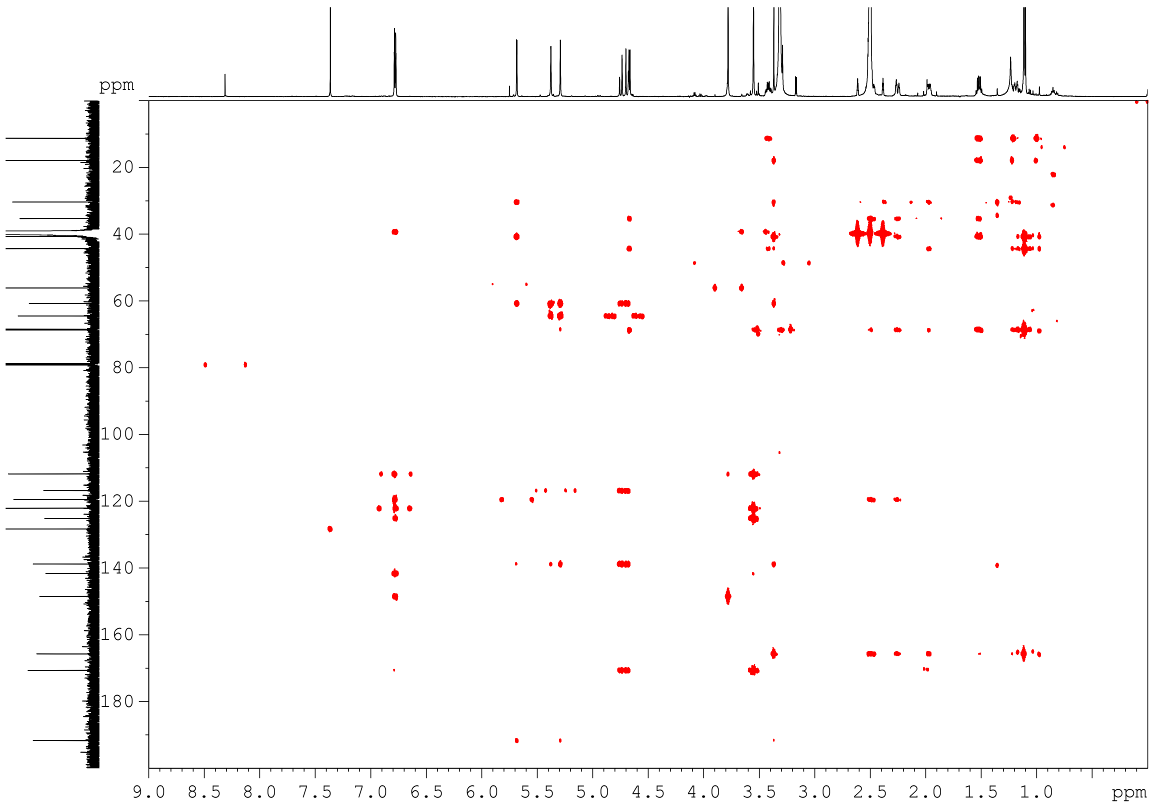

Figure S49. NOESY spectrum of **6** in DMSO-*d6*

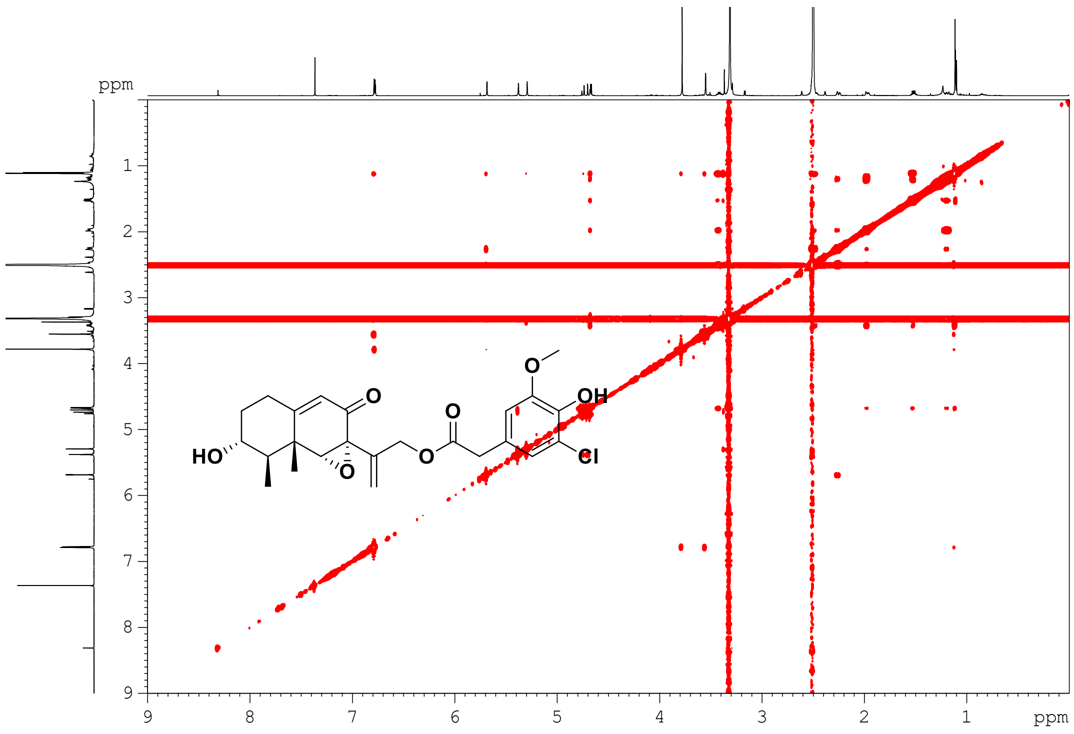

Figure S50. HRESIMS spectrum of **6**

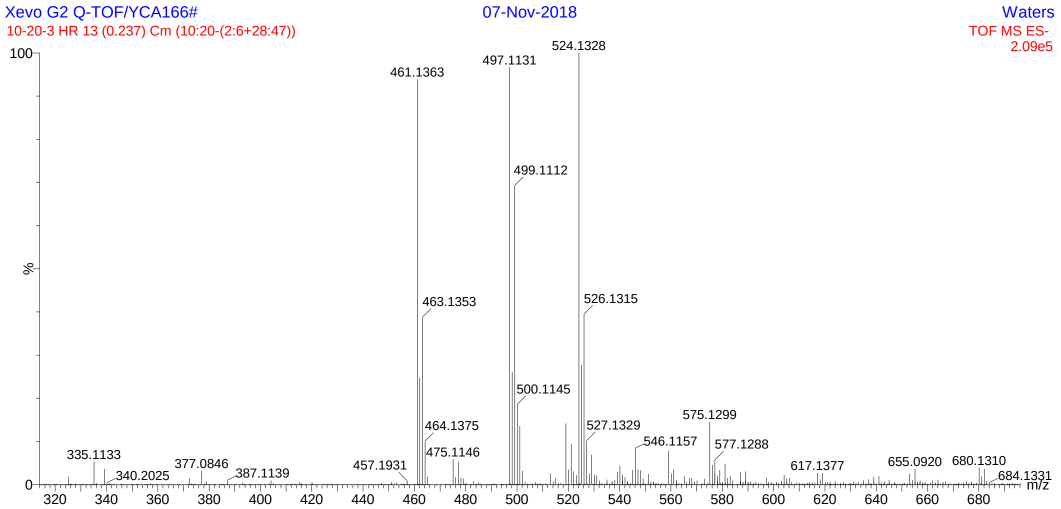

Mass	Calc. Mass	mDa	PPM	DBE	i-FIT	Norm	Conf(%)	Formula
461.1363	461.1367	-0.4	-0.9	11.5	185.3	0.000	100.00	$C_{24}H_{26}O_7Cl$

Figure S51. IR spectrum of **7**

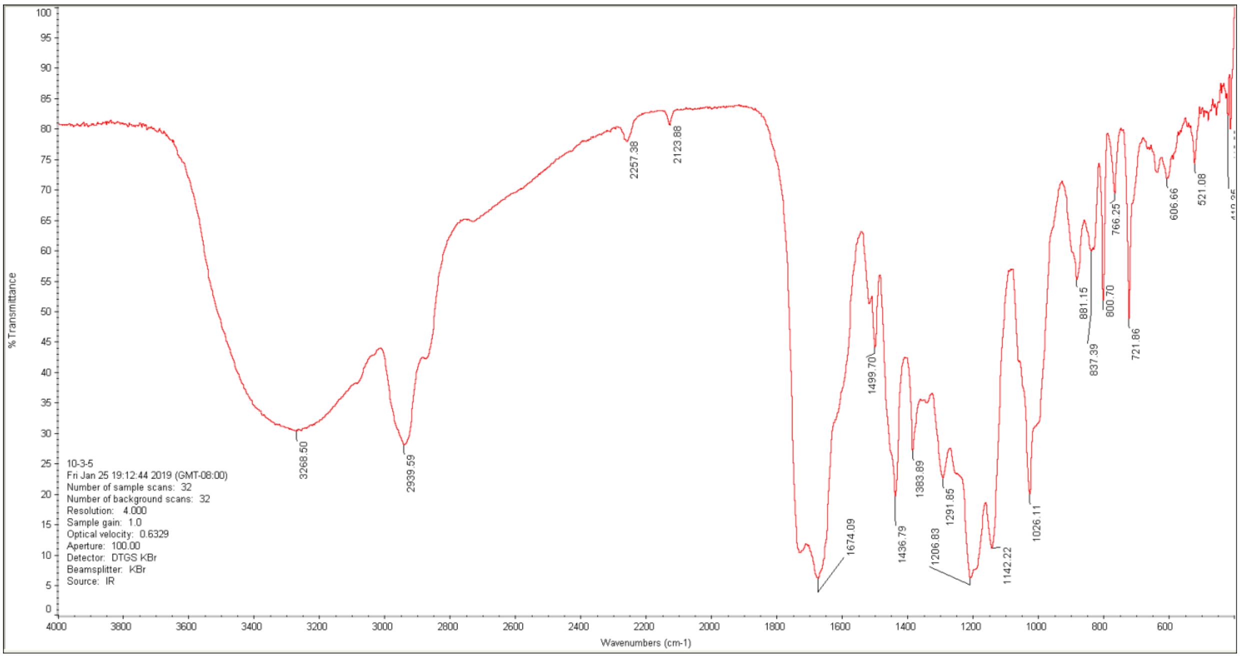

Figure S52. ^{1}H NMR (600 MHz) spectrum of **7** in DMSO-*d*6

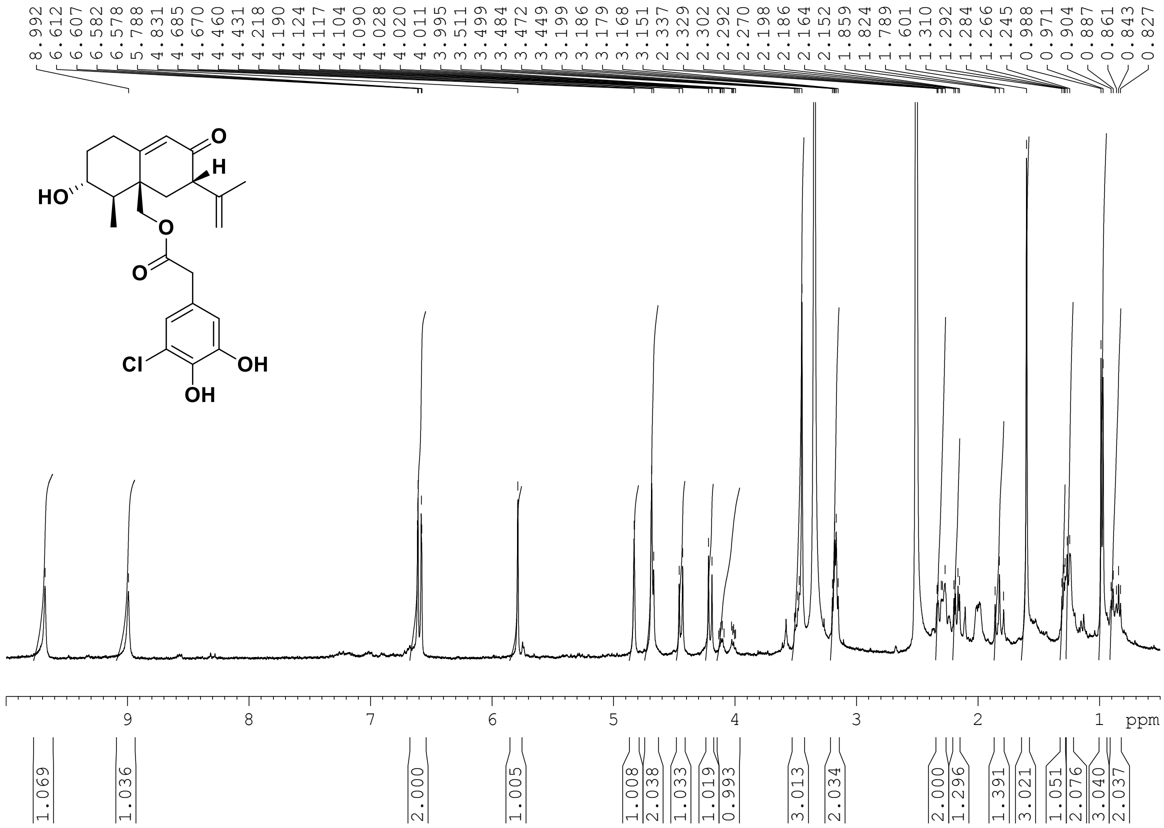

Figure S53. ^{13}C NMR (150 MHz) spectrum of **7** in DMSO-d_6

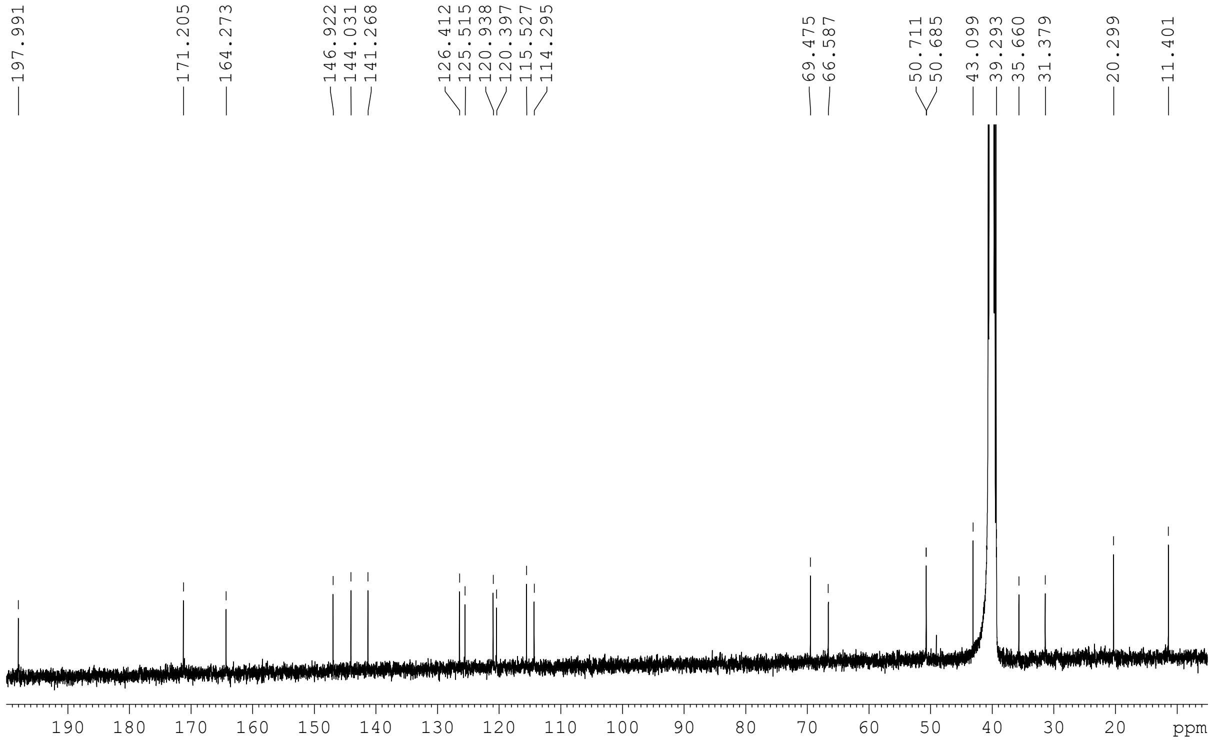

Figure S54. DEPT spectrum of **7** in DMSO-d_6

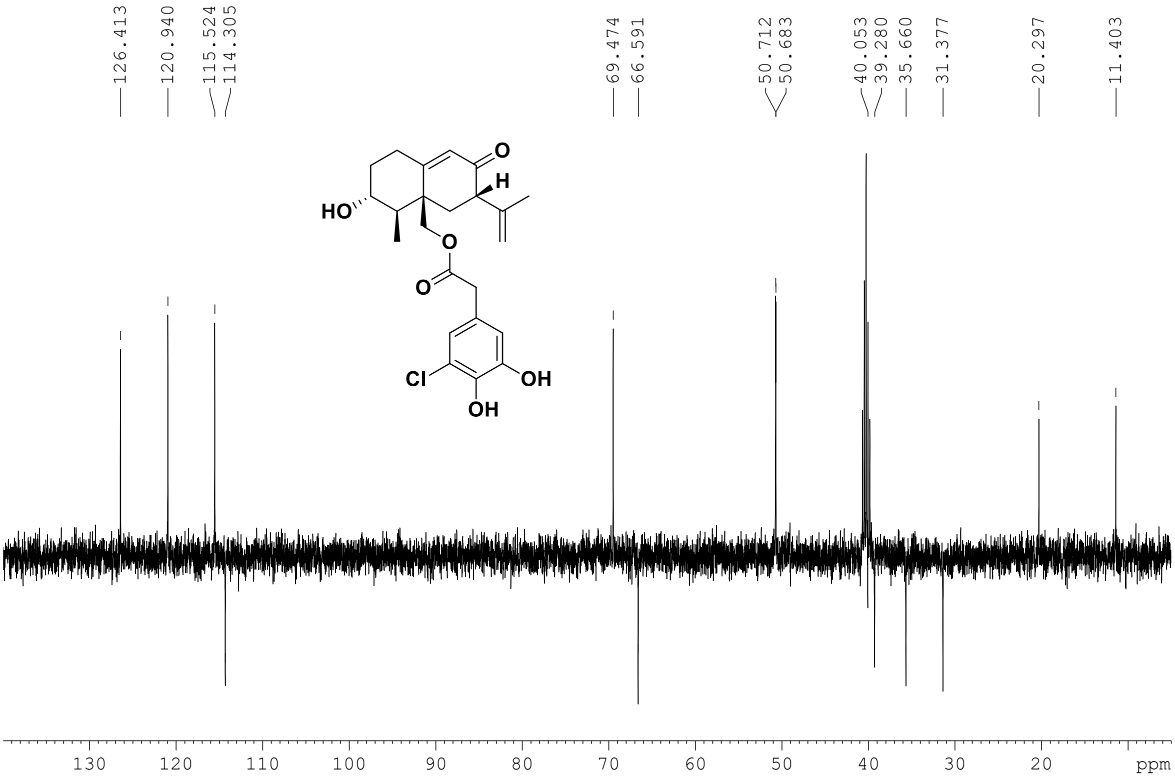

Figure S55. ^{1}H-^{1}H COSY spectrum of **7** in DMSO-*d*6

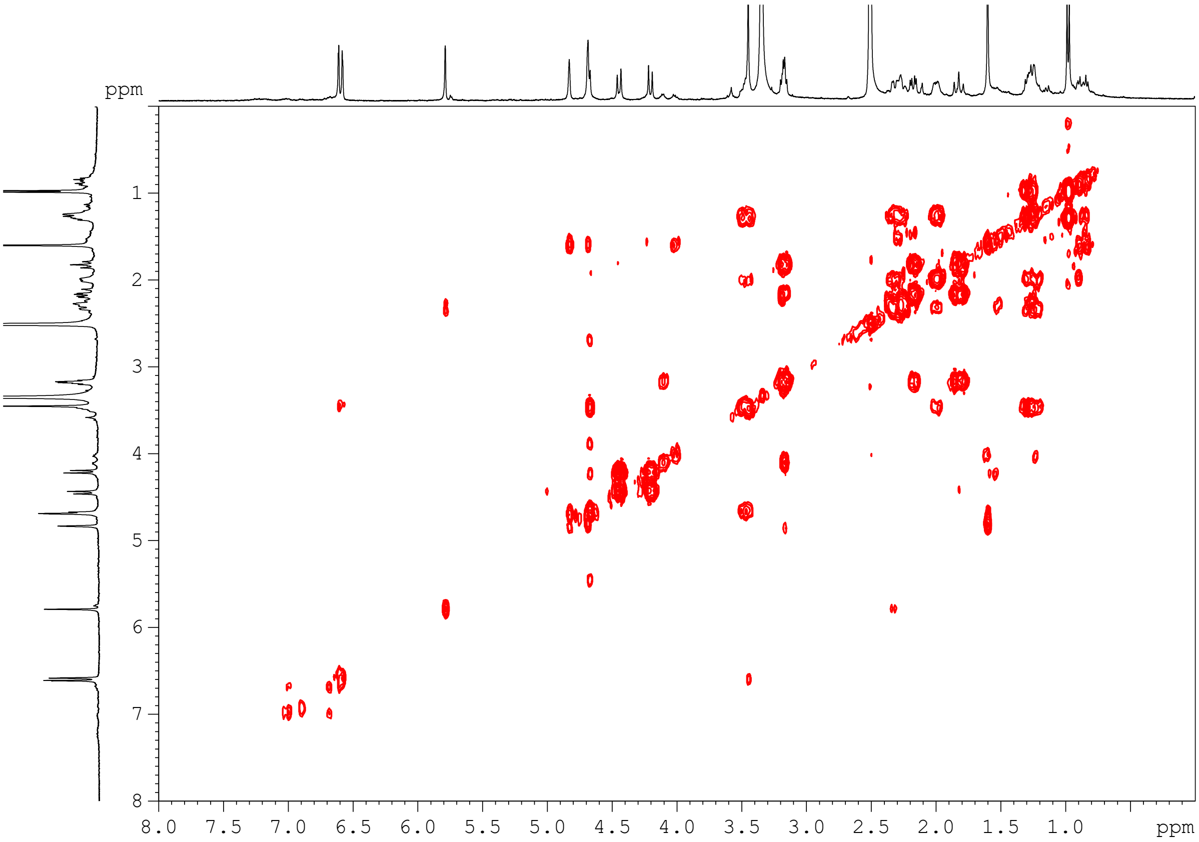

Figure S56. HSQC spectrum of **7** in DMSO-*d*6

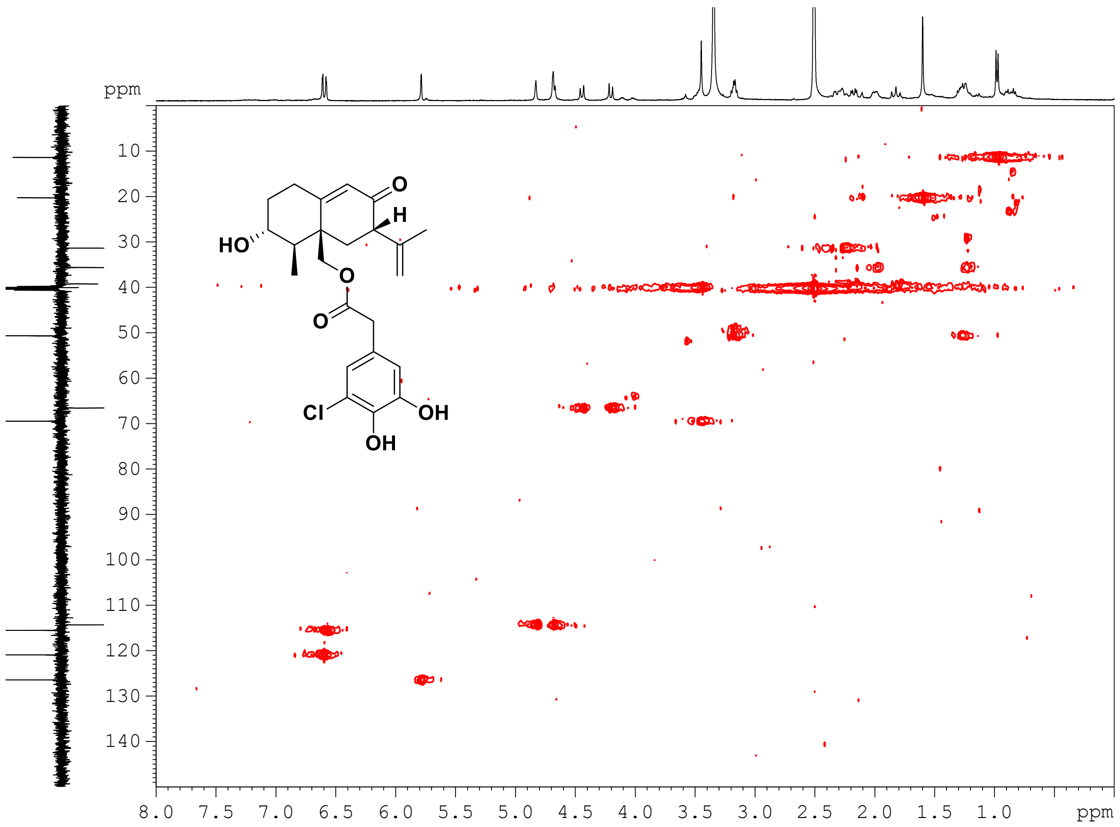

Figure S57. HMBC spectrum of **7** in DMSO-*d*6

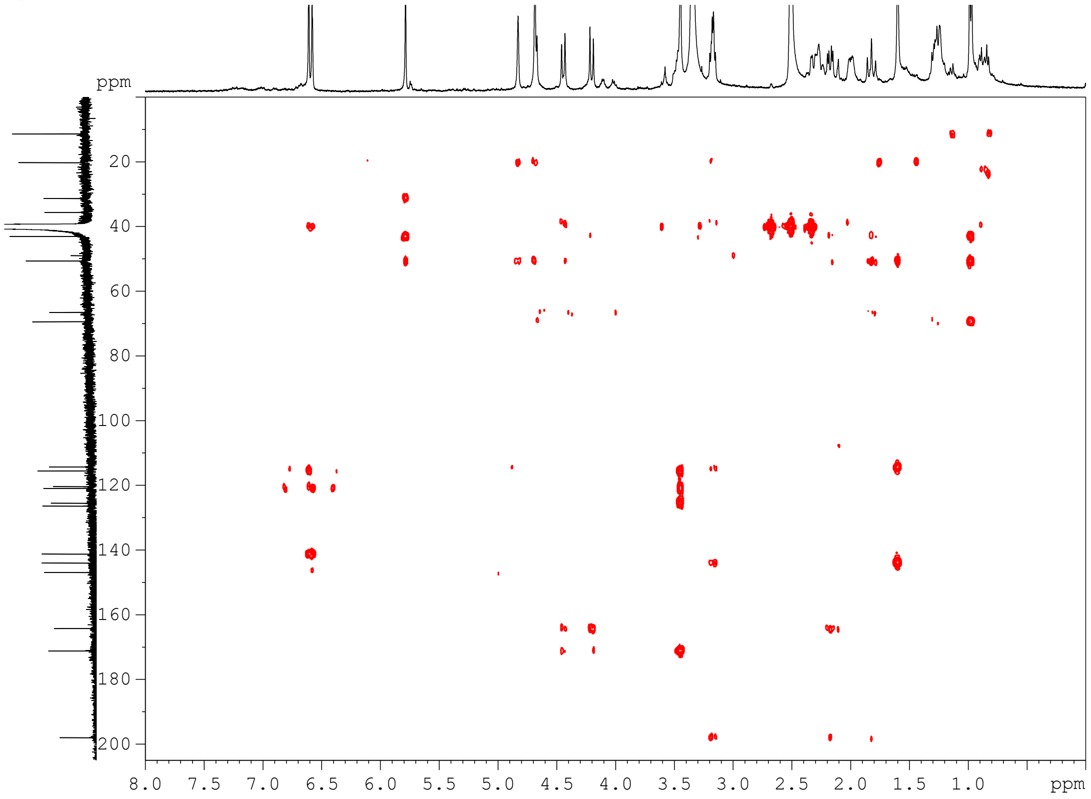

Figure S58. NOESY spectrum of **7** in DMSO-*d6*

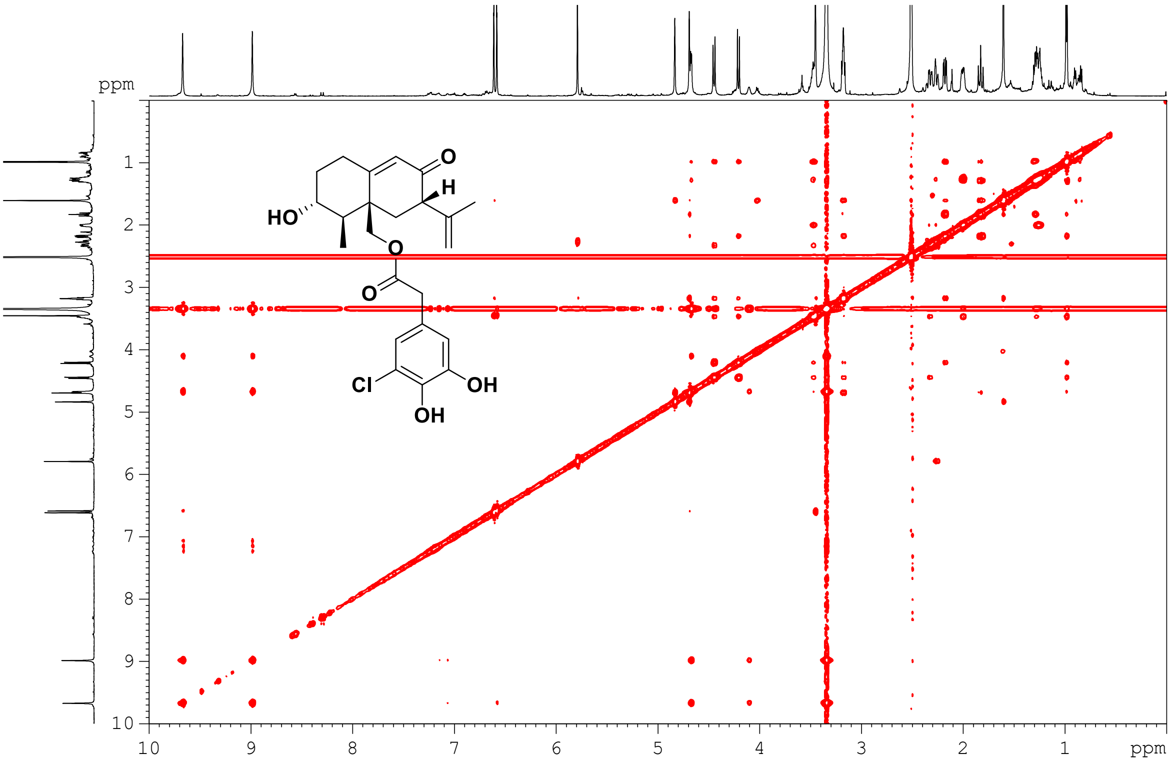

Figure S59. HRESIMS spectrum of **7**

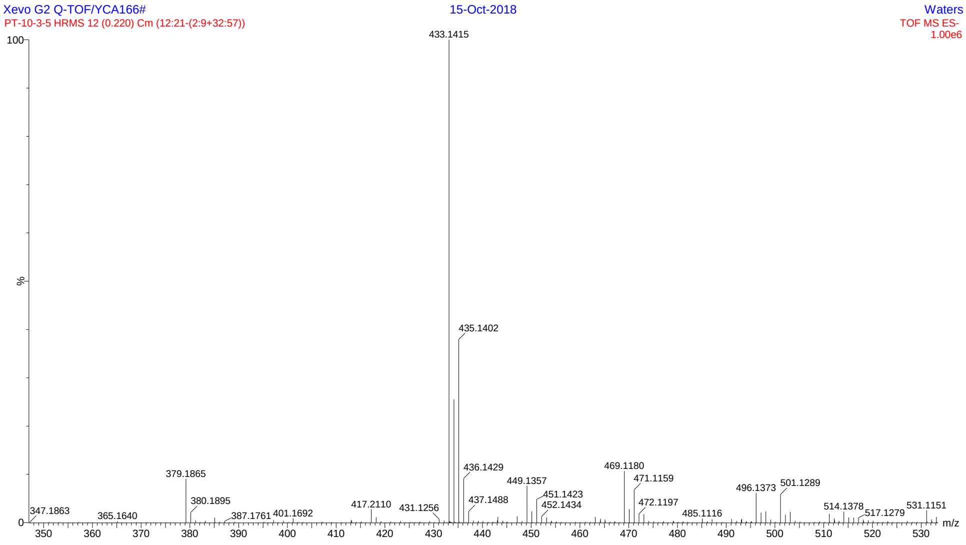

Mass	Calc. Mass	mDa	PPM	DBE	i-FIT	Norm	Conf(%)	Formula
443.1415	443.1418	-0.3	-0.7	10.5	646.7	n/a	n/a	$C_{23}H_{26}O_6Cl$

Figure S60. IR spectrum of **8**

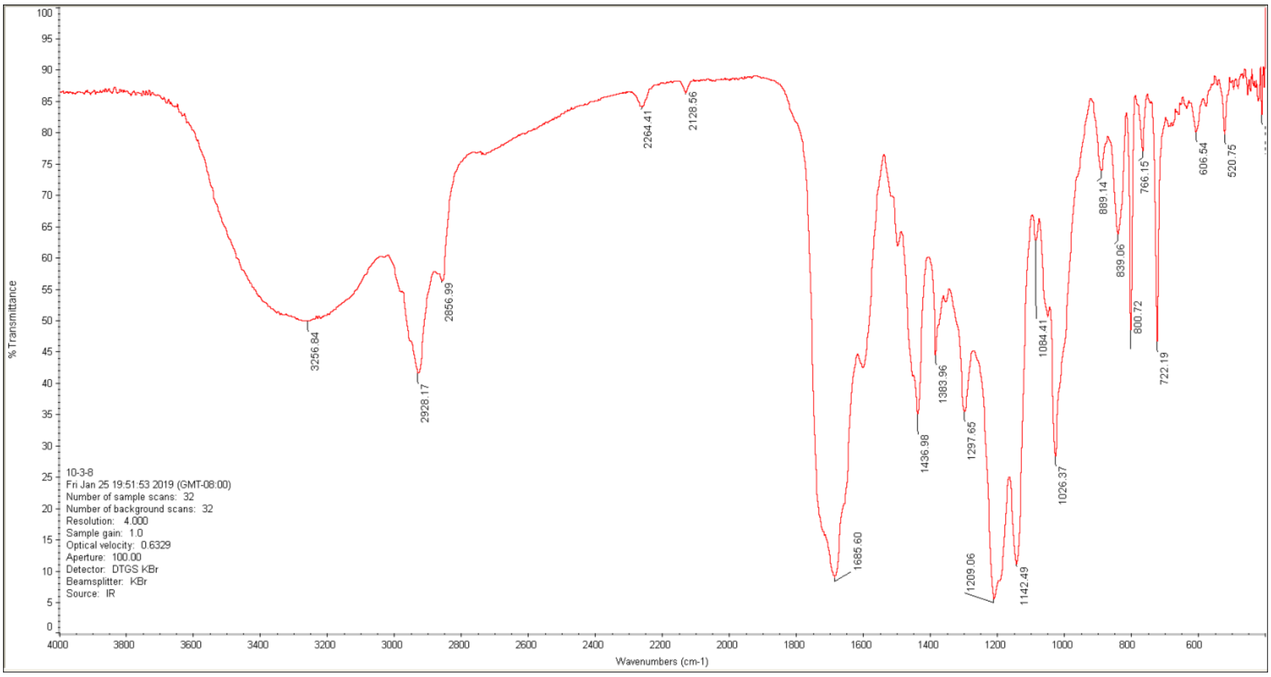

Figure S61. ^{1}H NMR (600 MHz) spectrum of **8** in DMSO-*d6*

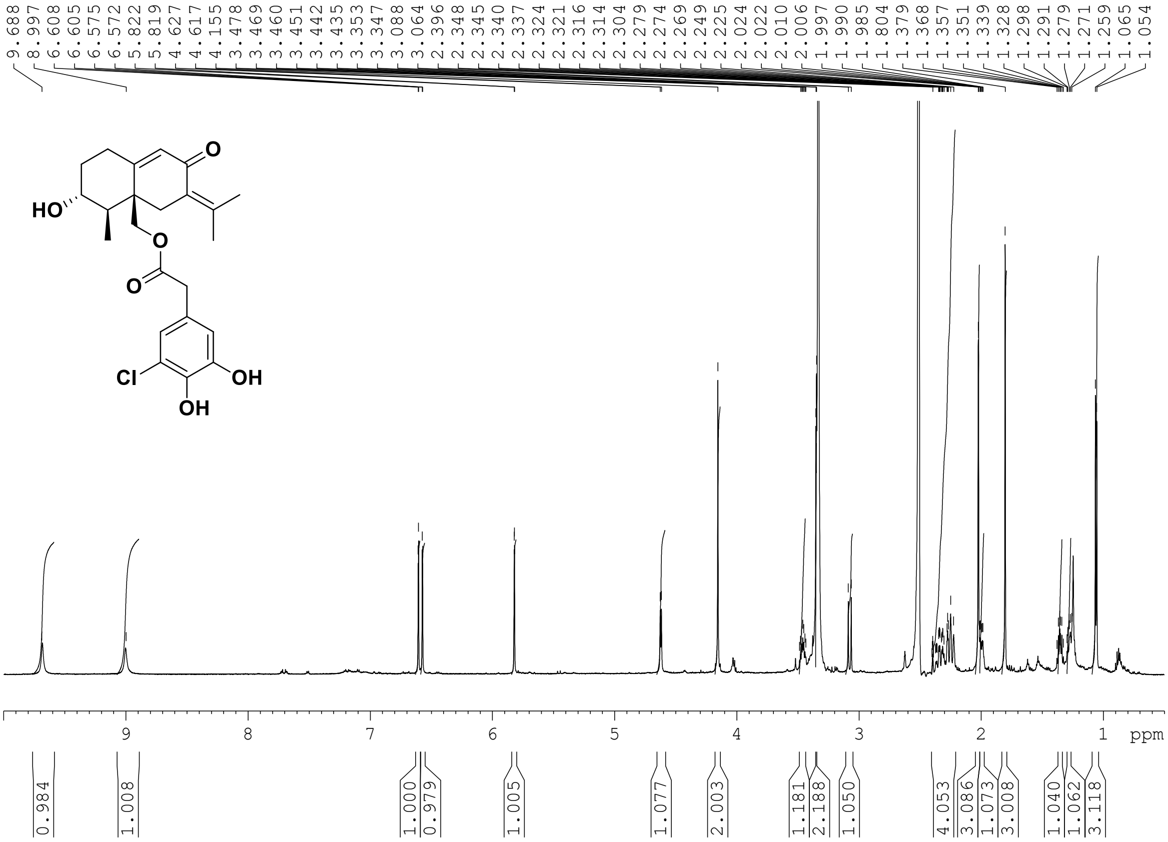

Figure S62. ^{13}C NMR (150 MHz) and DEPT spectra of **8** in DMSO-d_6

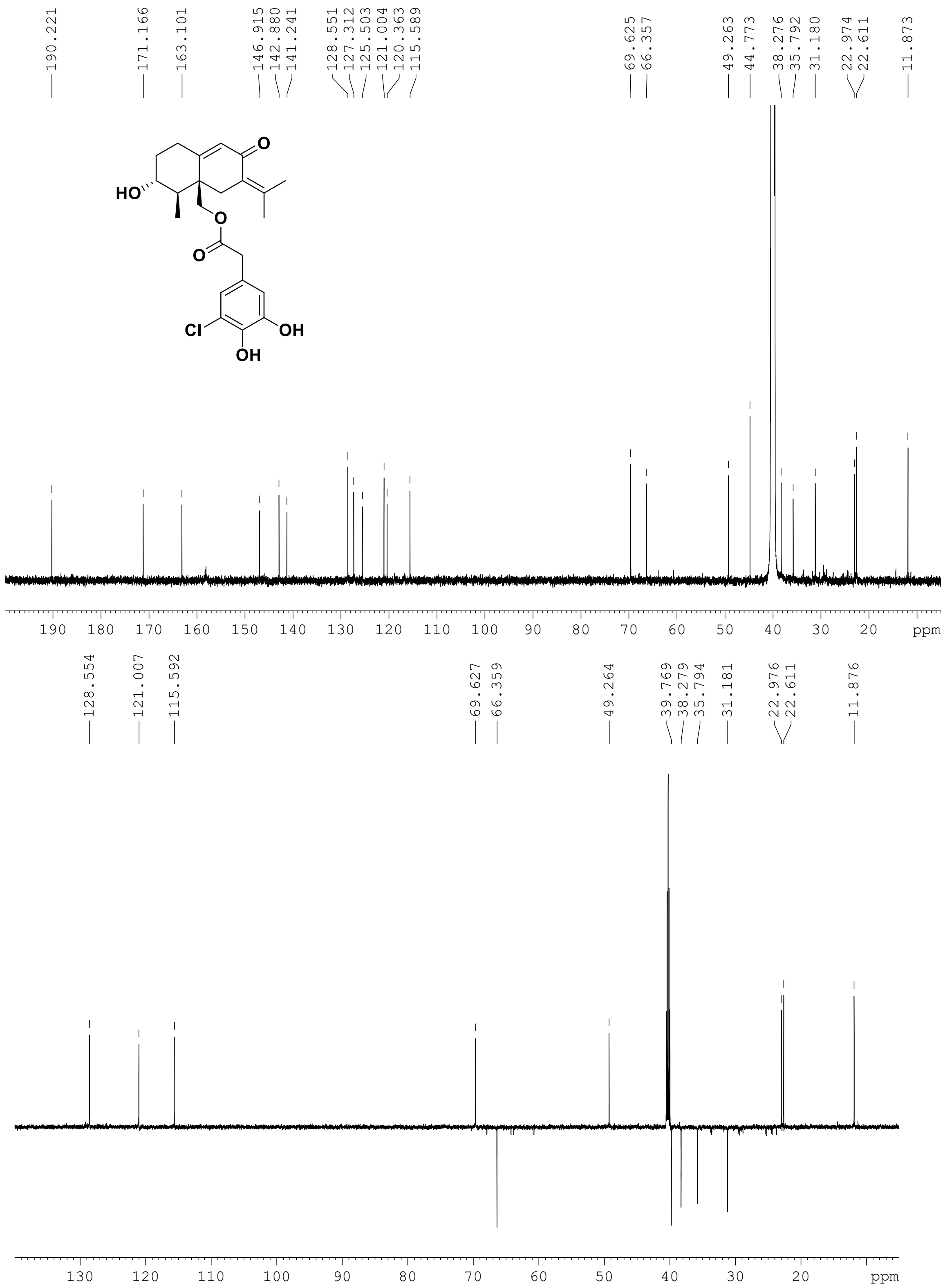

Figure S63. ^{1}H-^{1}H COSY spectrum of **8** in DMSO-*d6*

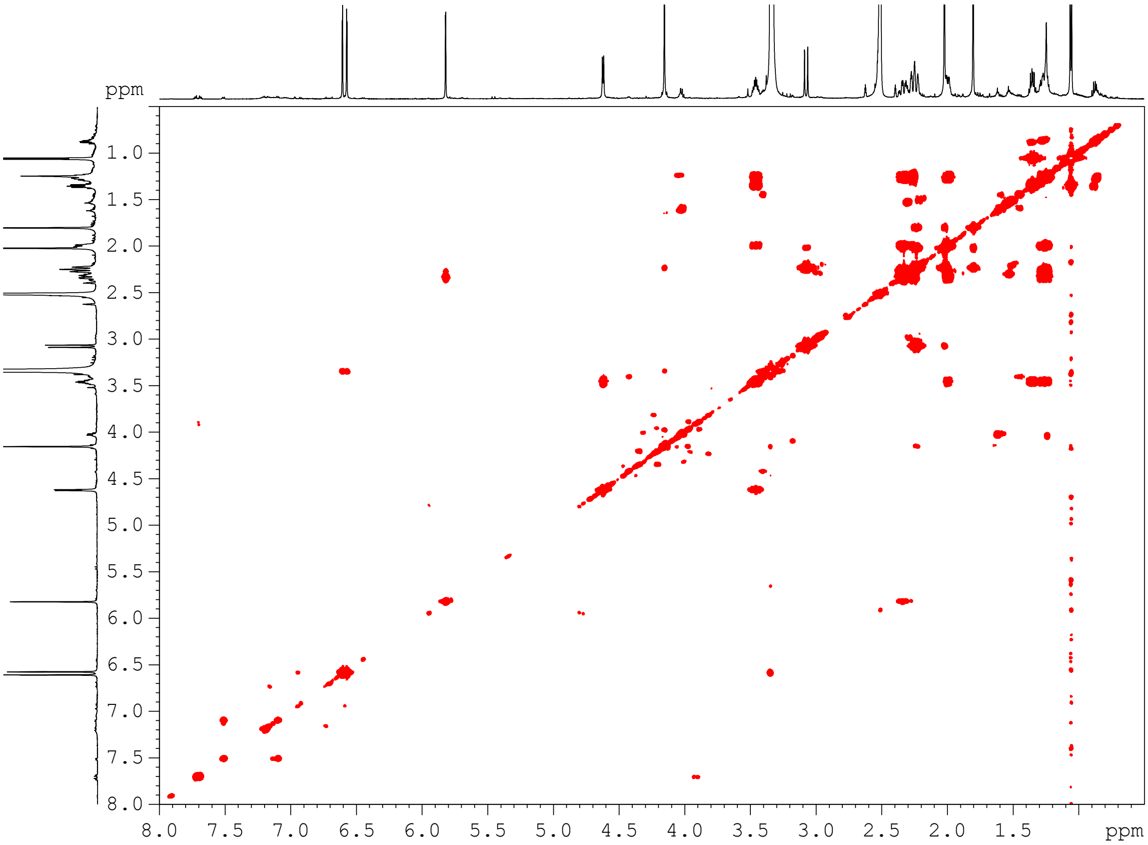

Figure S64. HSQC spectrum of **8** in DMSO-*d6*

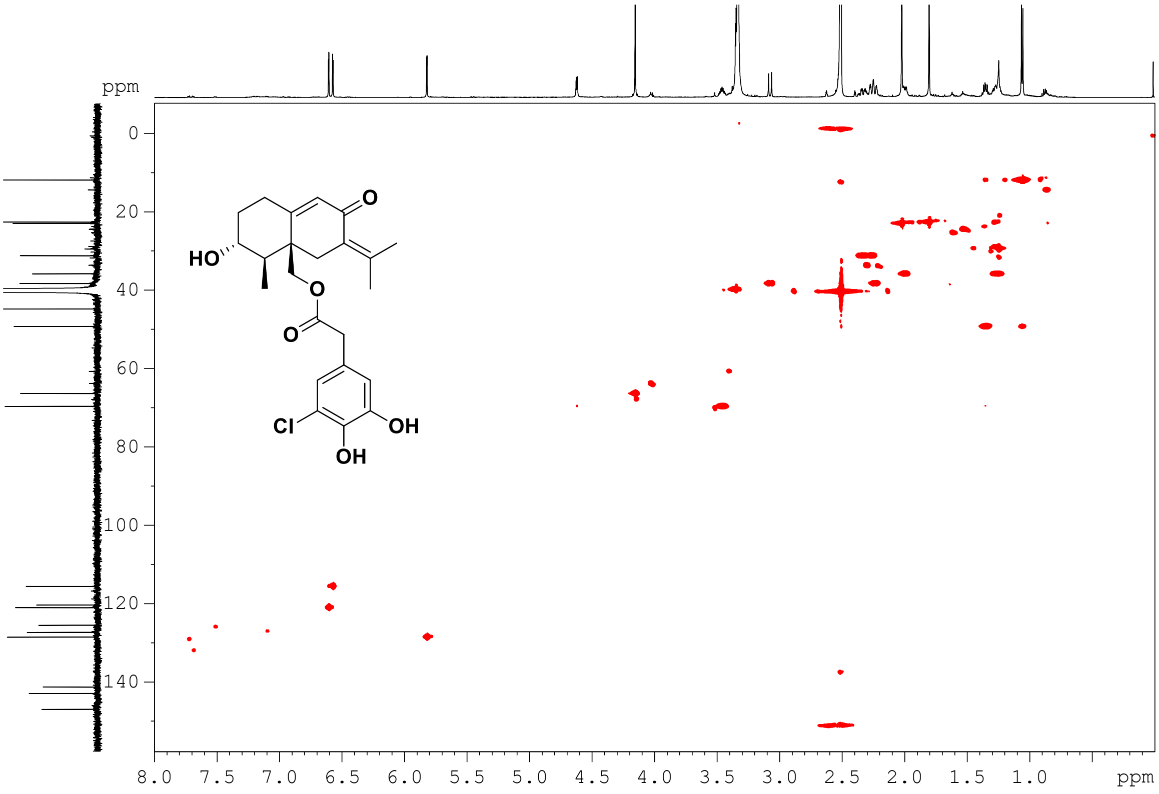

Figure S65. HMBC spectrum of **8** in DMSO-*d*6

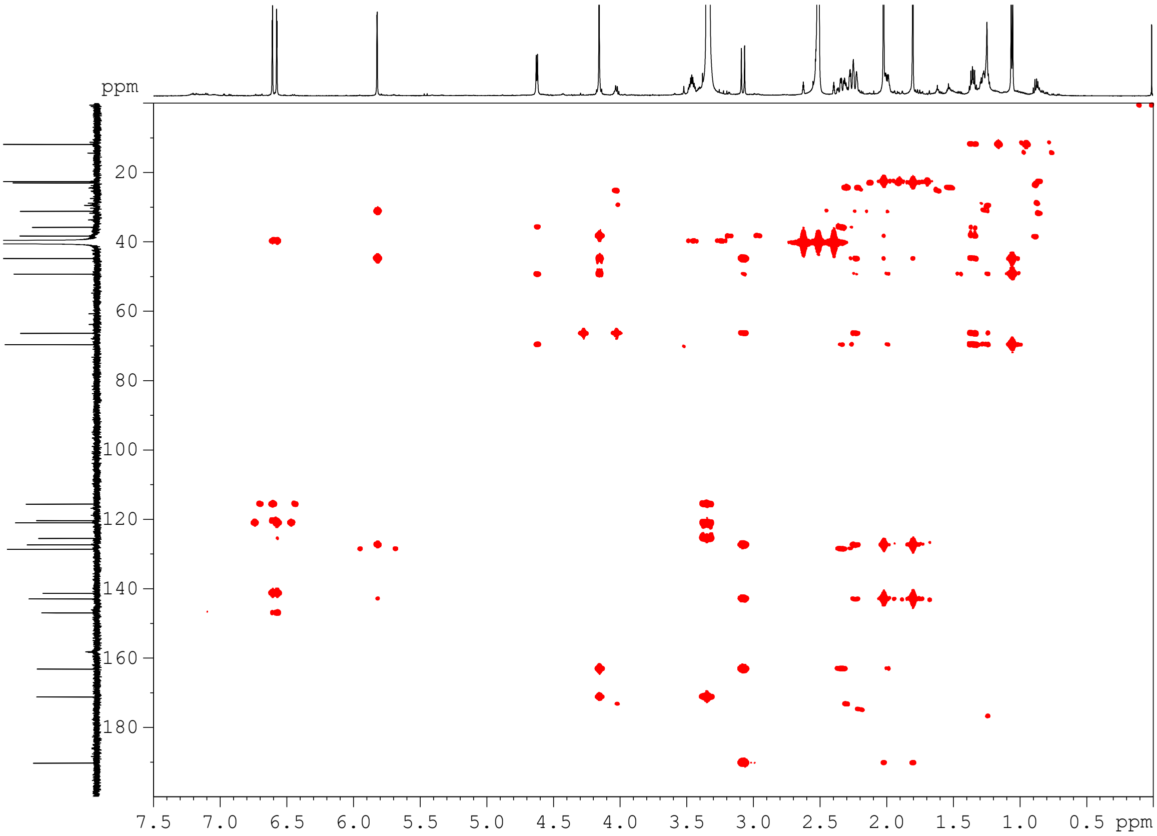

Figure S66. NOESY spectrum of **8** in DMSO-*d*6

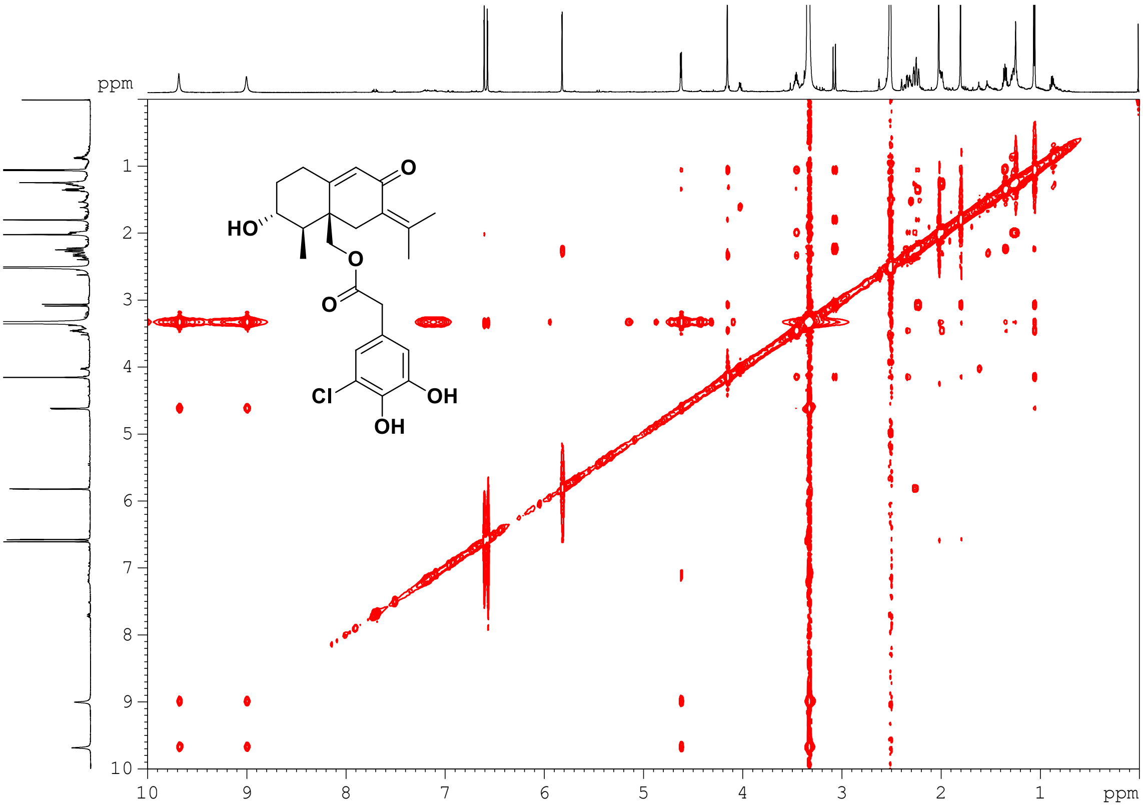

Figure S67. HRESIMS spectrum of **8**

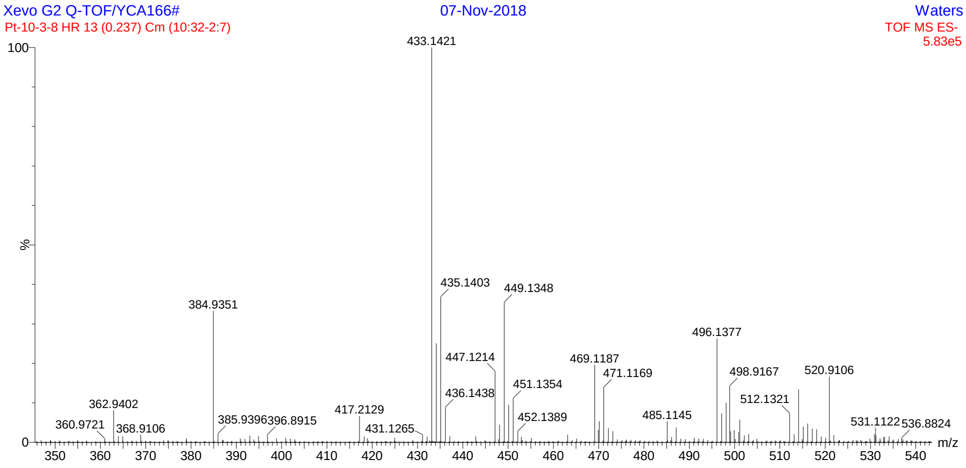

Mass	Calc. Mass	mDa	PPM	DBE	i-FIT	Norm	Conf(%)	Formula
443.1421	443.1418	0.3	0.7	10.5	610.1	0.000	100.00	$C_{23}H_{26}O_6Cl$

Figure S68. IR spectrum of **9**

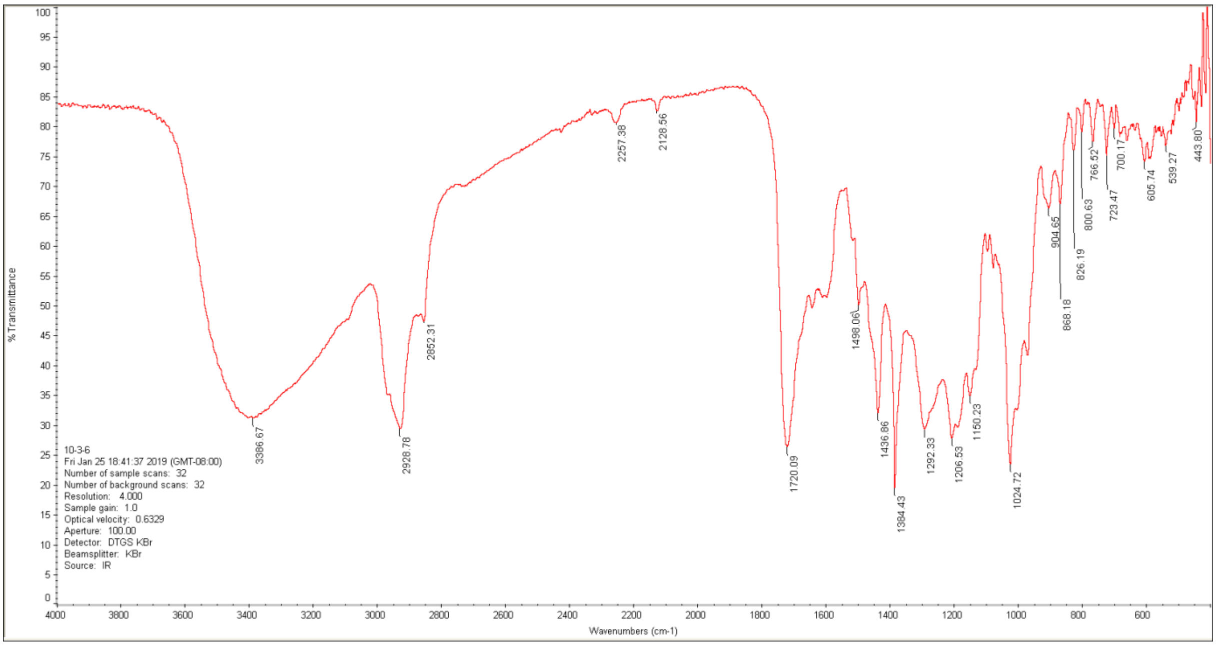

Figure S69. ^{1}H NMR (600 MHz) spectrum of **9** in DMSO-*d*6

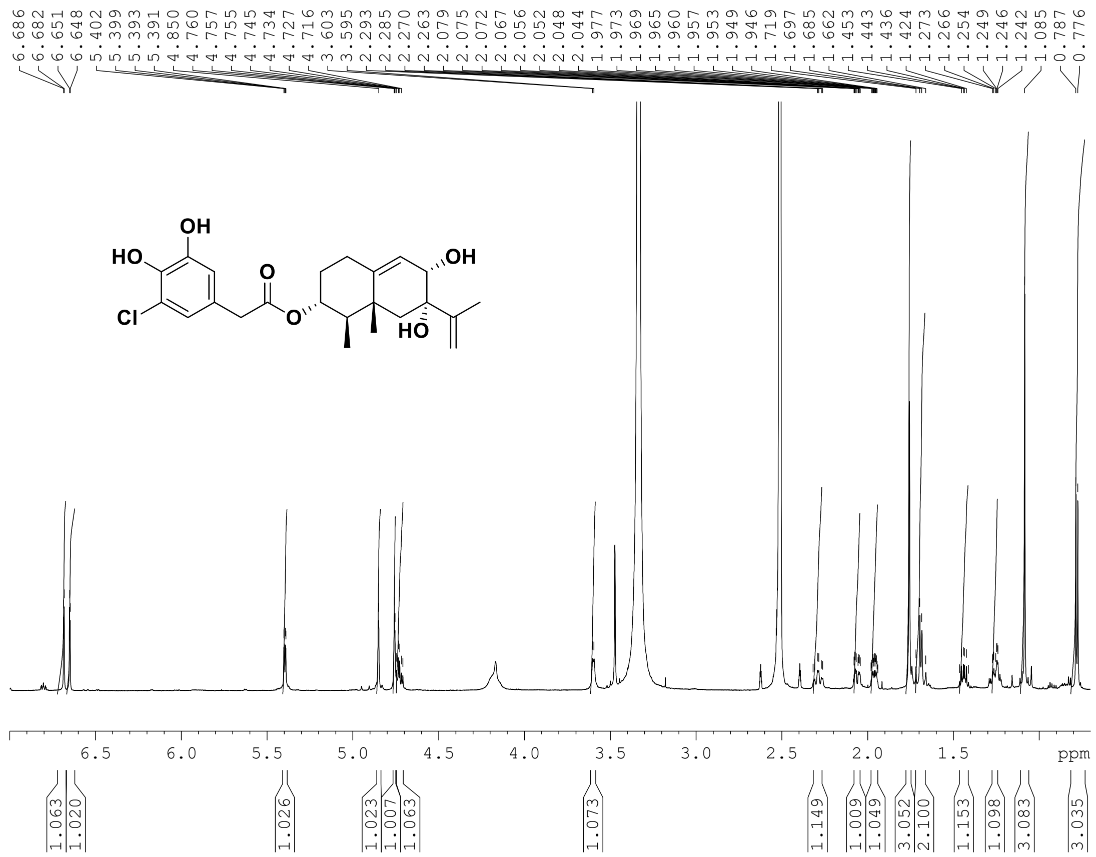

Figure S70. ^{13}C NMR (150 MHz) spectrum of **9** in DMSO-d_6

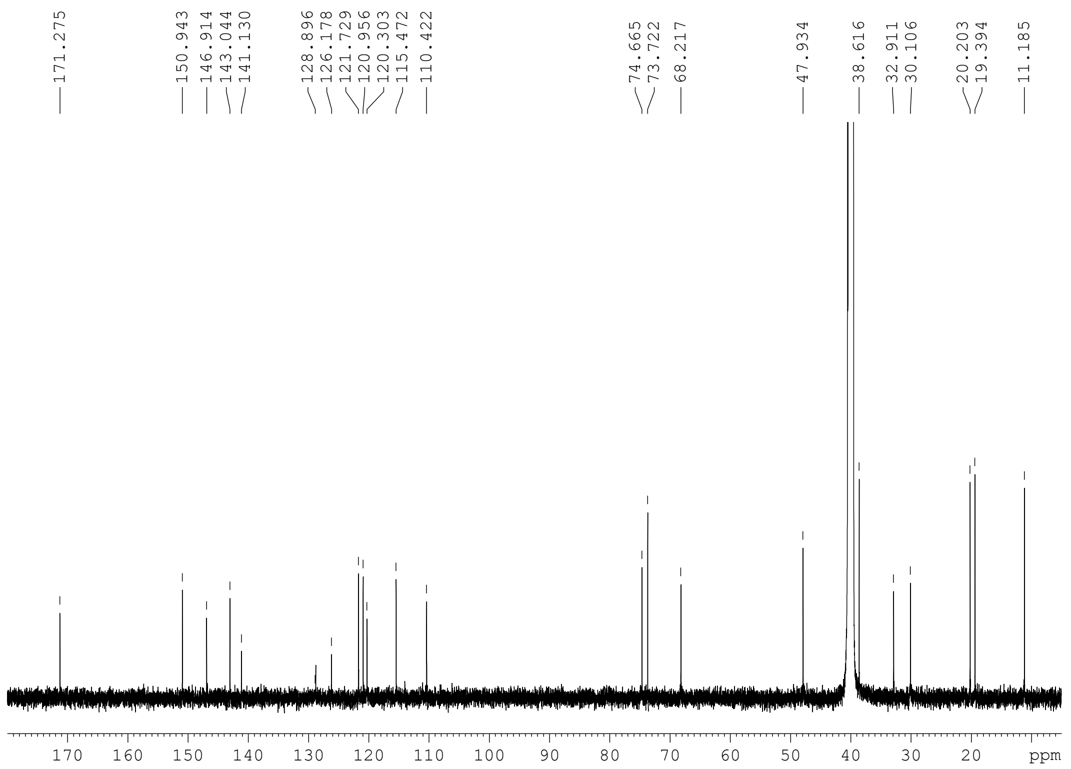

Figure S71. DEPT spectrum of **9** in DMSO-d_6

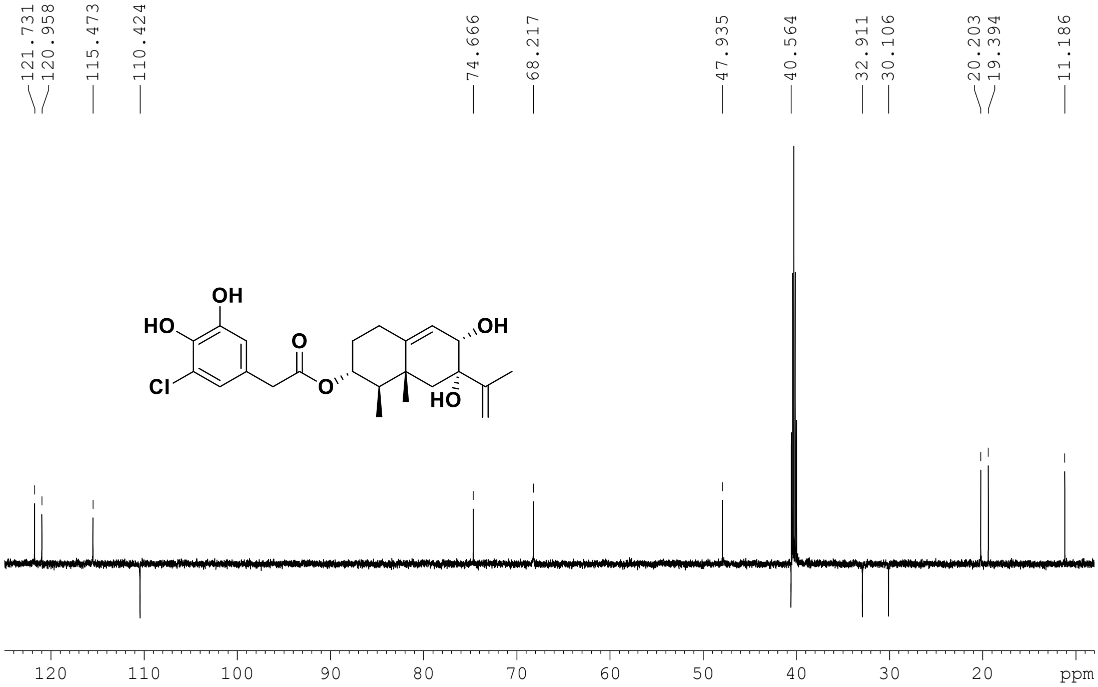

Figure S72. ^{1}H-^{1}H COSY spectrum of **9** in DMSO-$d6$

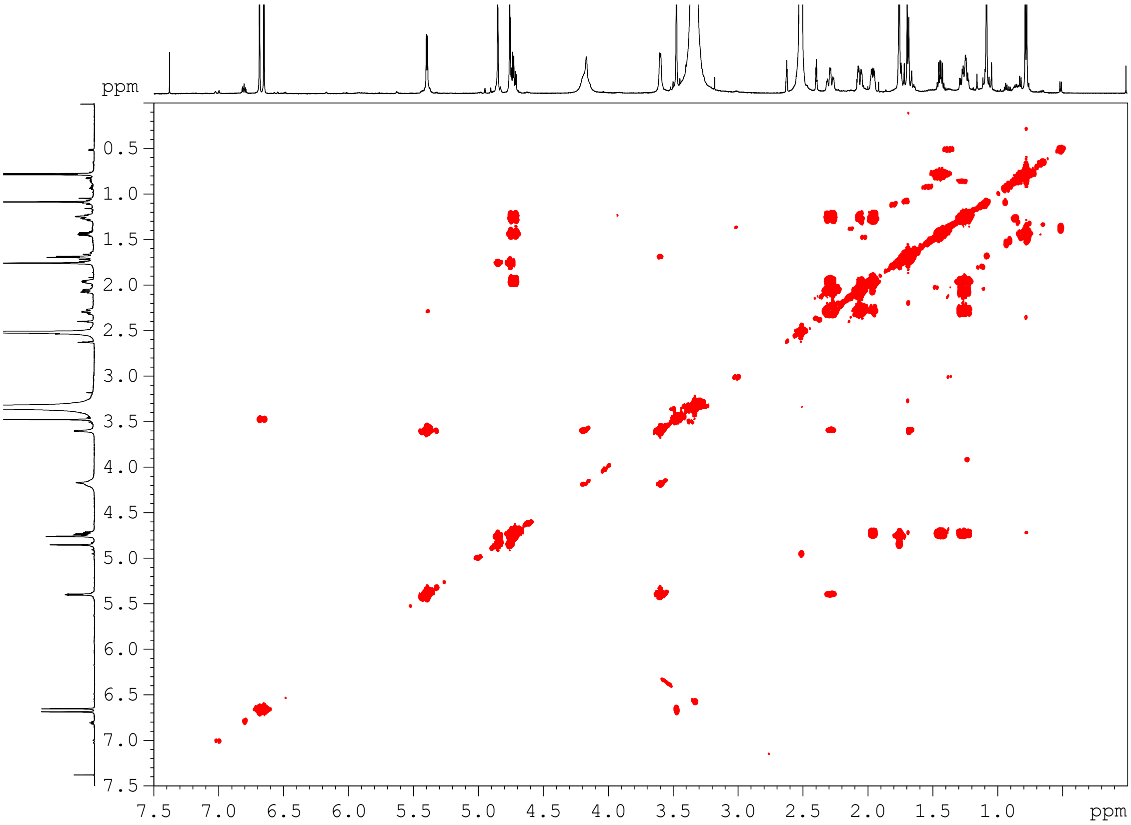

Figure S73. HSQC spectrum of **9** in DMSO-*d6*

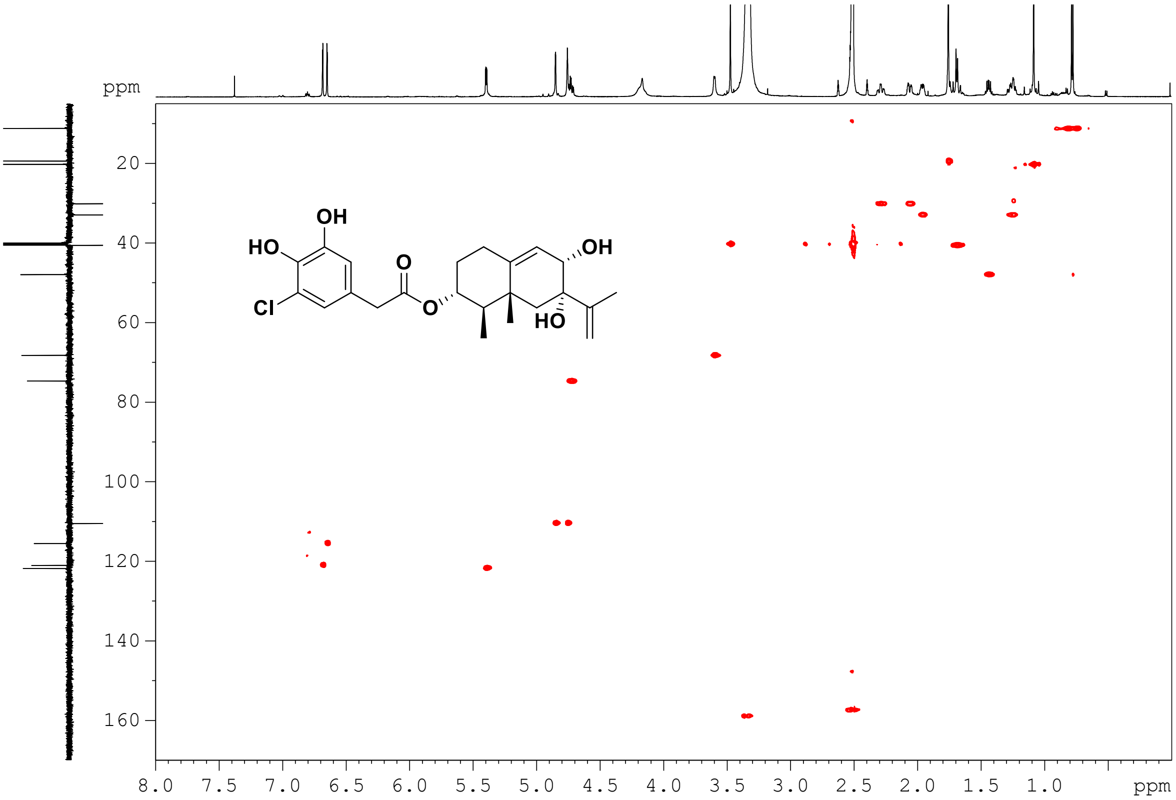

Figure S74. HMBC spectrum of **9** in DMSO-*d6*

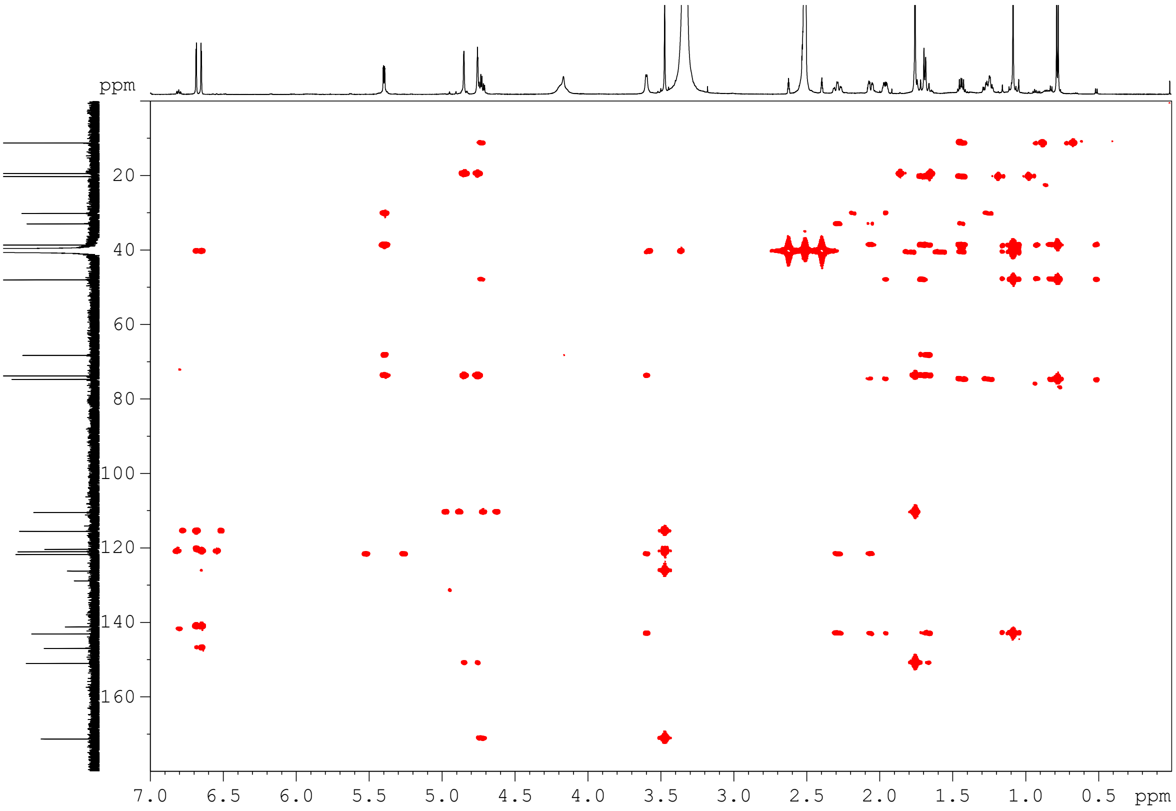

Figure S75. NOESY spectrum of **9** in DMSO-*d*6

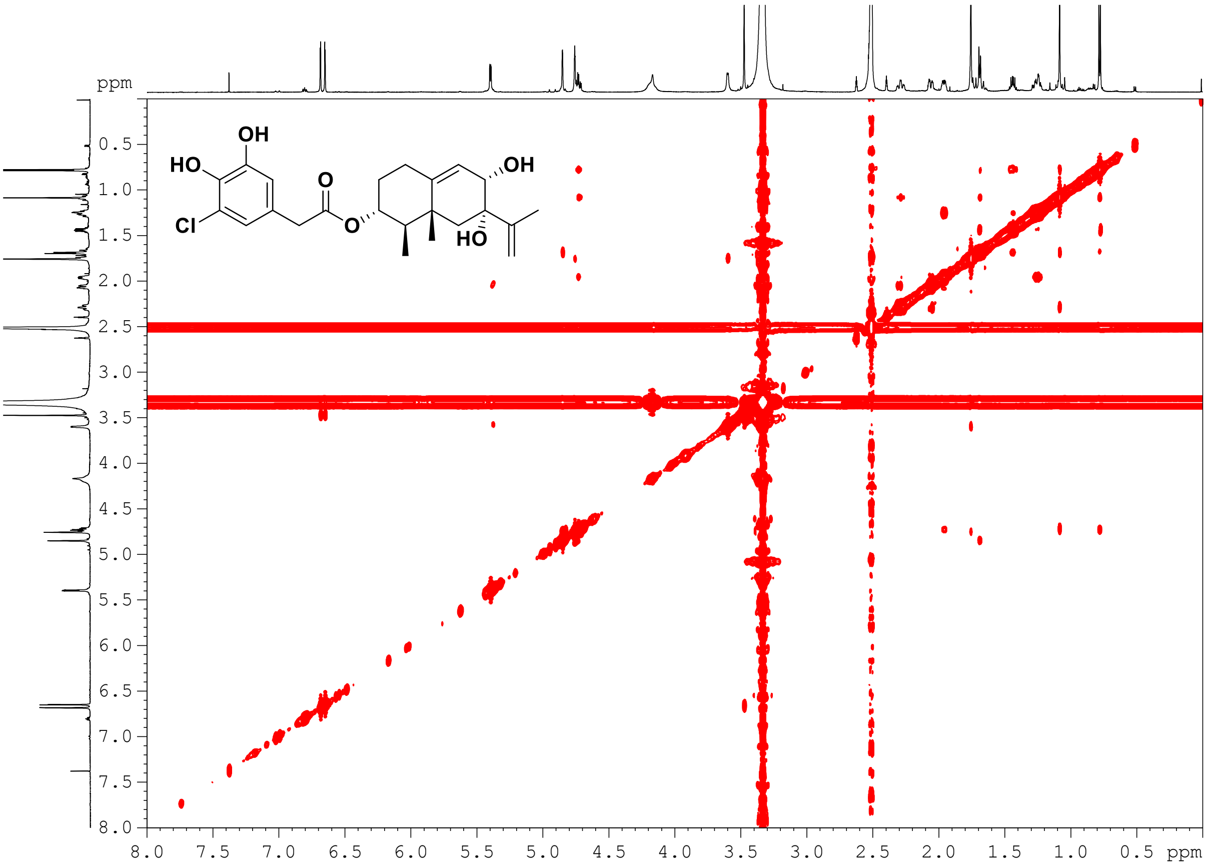

Figure S76. HRESIMS spectrum of **9**

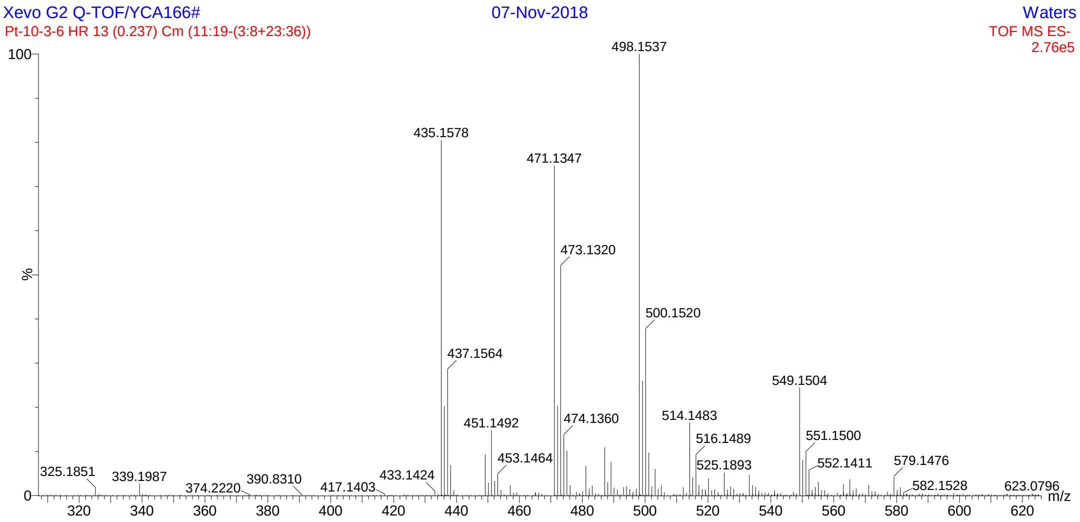

Mass	Calc. Mass	mDa	PPM	DBE	i-FIT	Norm	Conf(%)	Formula
435.1578	435.1574	0.4	0.9	9.5	257.3	0.000	100.00	$C_{23}H_{28}O_6Cl$

Figure S77. IR spectrum of **10**

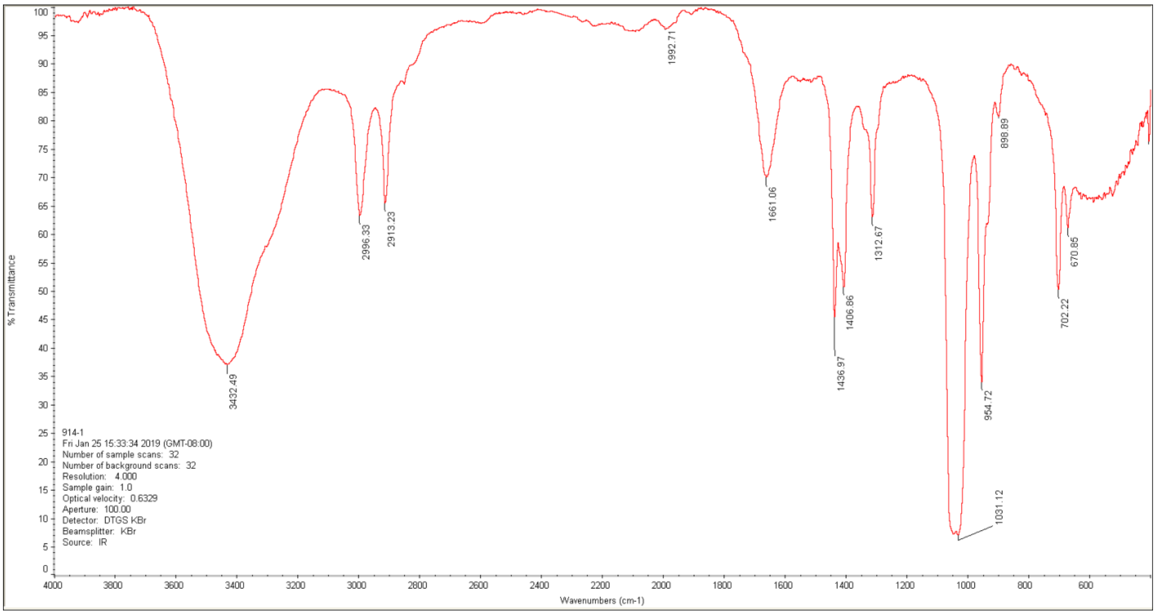

Figure S78. ^{1}H NMR (600 MHz) spectrum of **10** in DMSO-*d6*

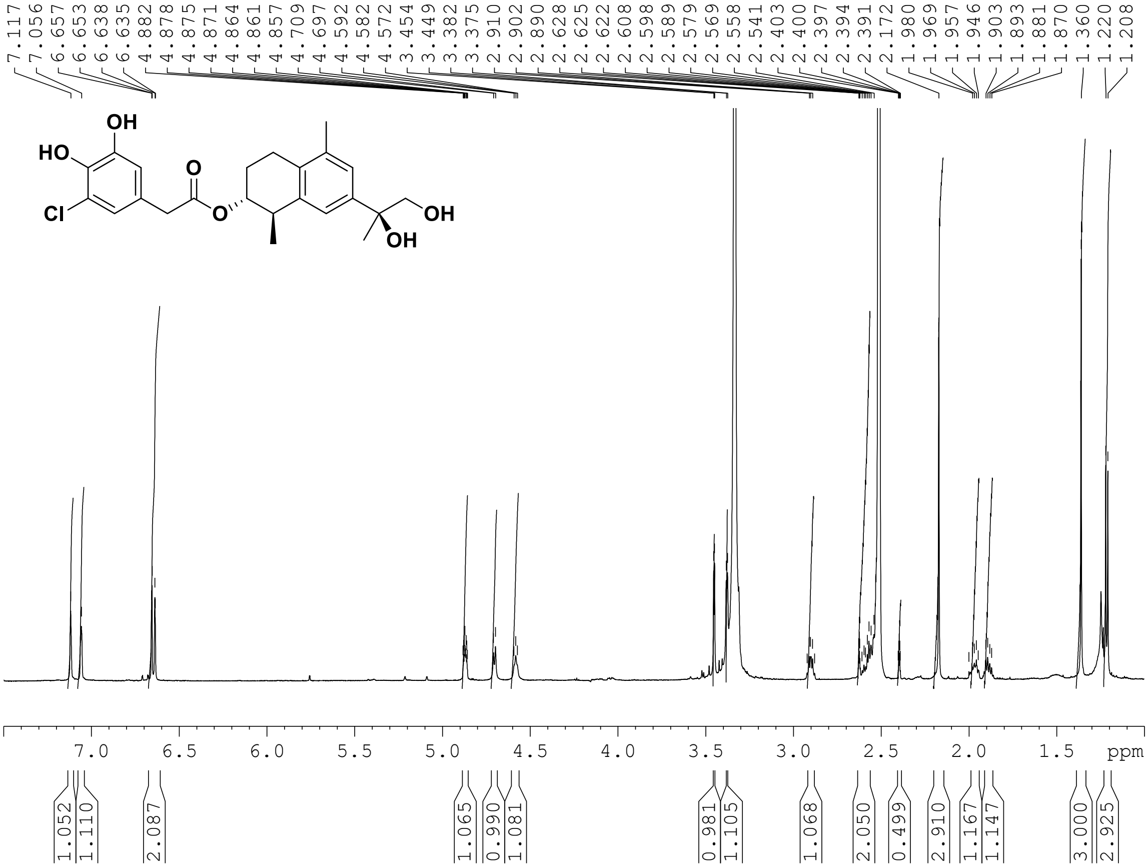

Figure S79. ^{13}C NMR (150 MHz) and DEPT spectra of **10** in DMSO-d_6

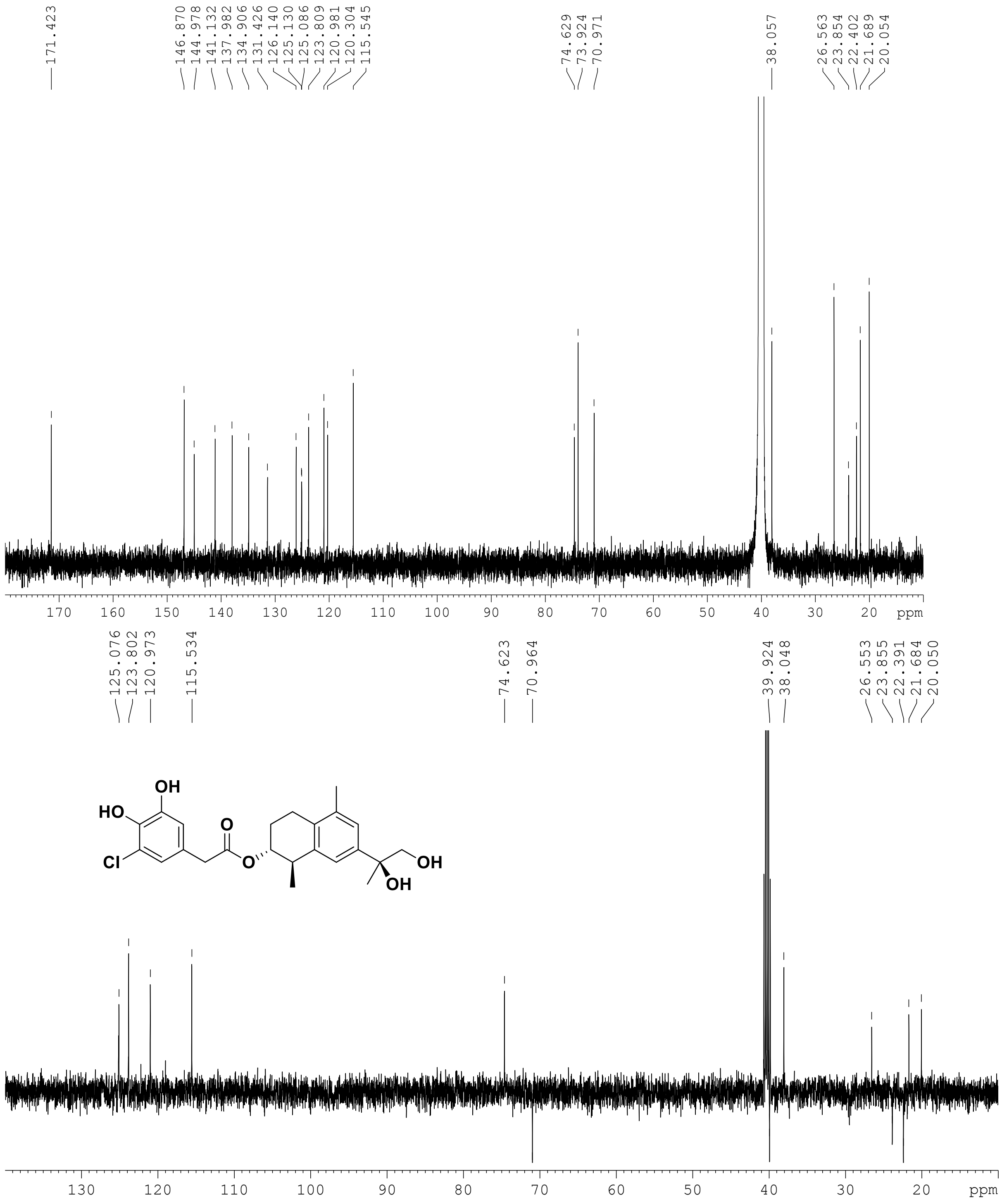

Figure S80. ^{1}H-^{1}H COSY spectrum of **10** in DMSO-*d*6

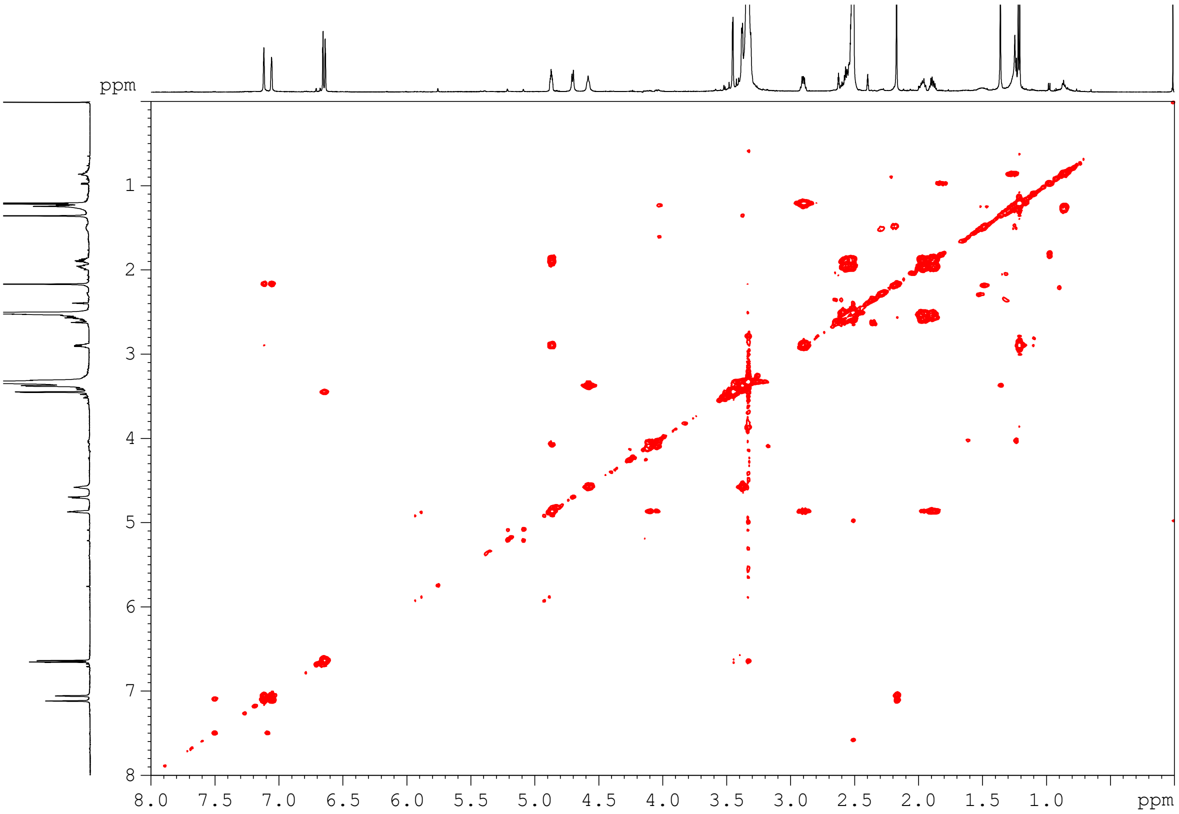

Figure S81. HSQC spectrum of **10** in DMSO-*d*6

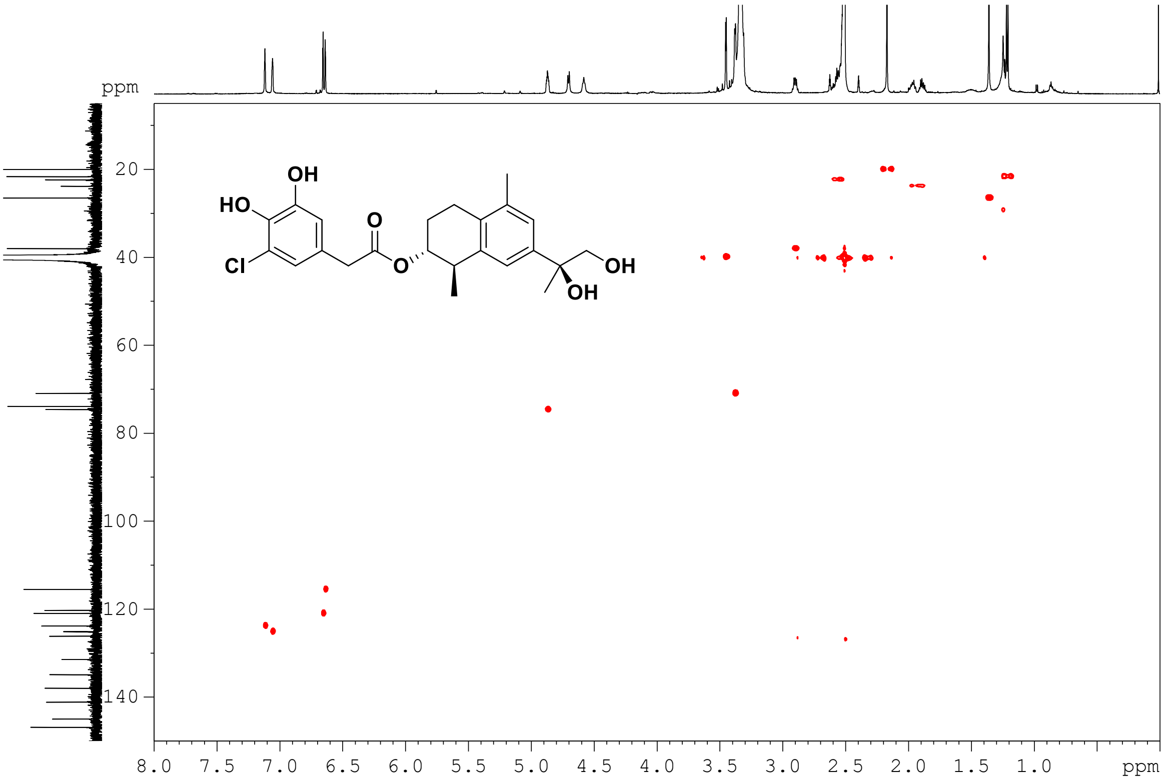

Figure S82. HMBC spectrum of **10** in DMSO-*d*6

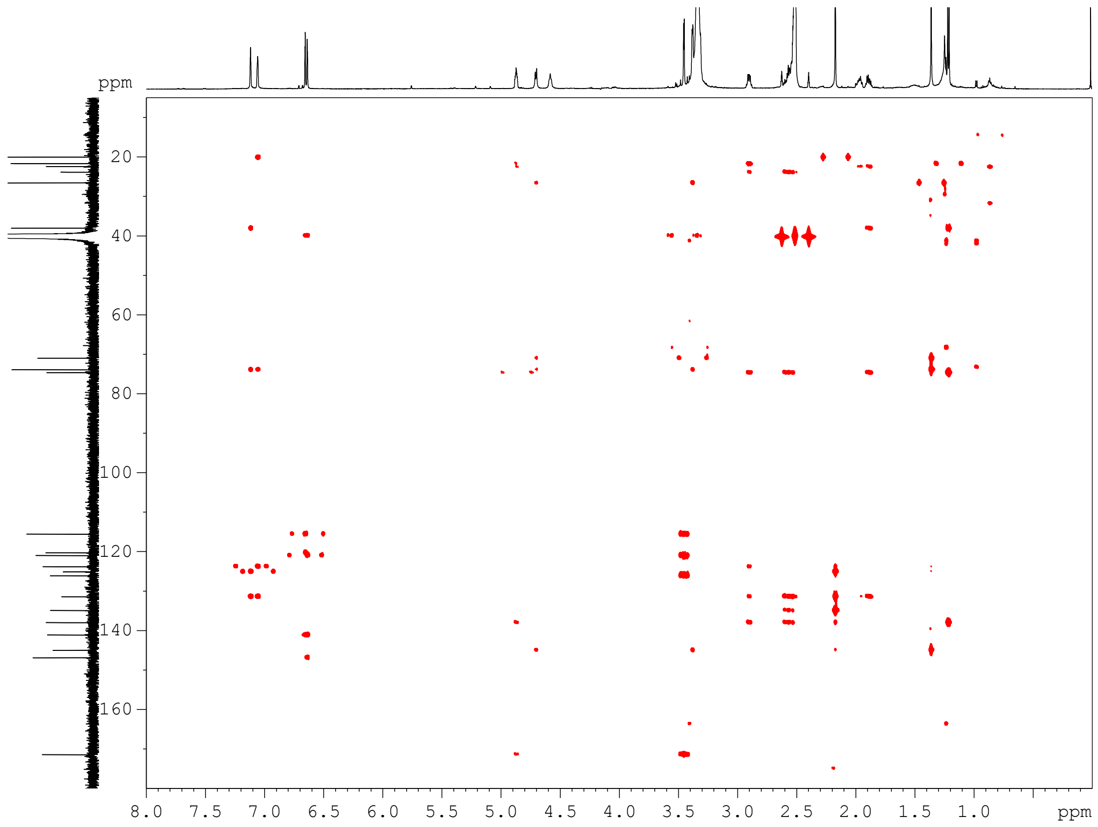

Figure S83. NOESY spectrum of **10** in DMSO-*d*6

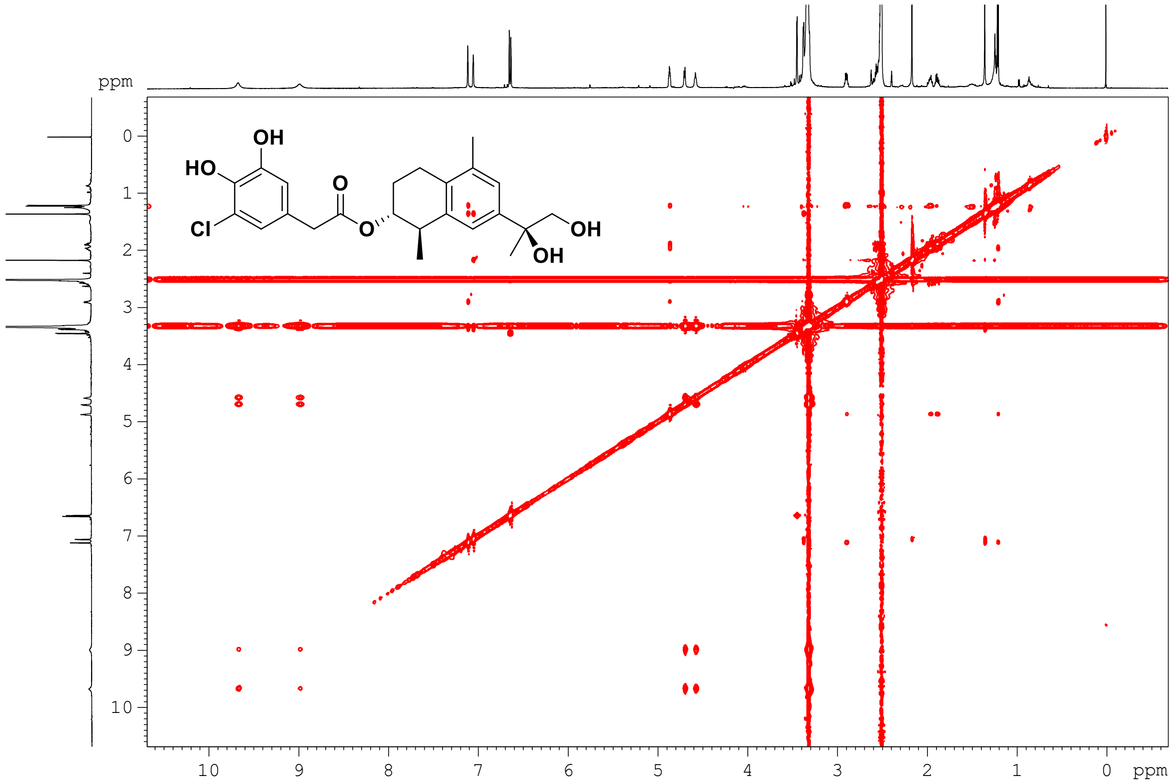

Figure S84. HRESIMS spectrum of **10** in DMSO-*d6*

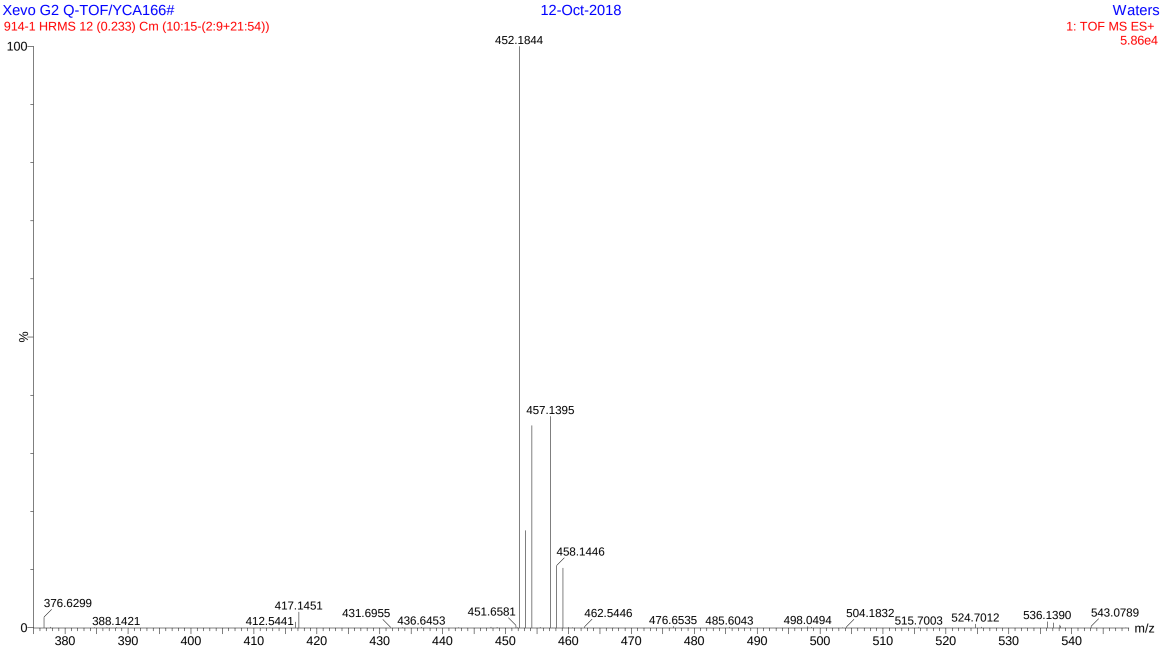

Mass	Calc. Mass	mDa	PPM	DBE	i-FIT	Norm	Conf(%)	Formula
457.1395	457.1390	0.4	0.9	0.5	34.0	n/a	n/a	$C_{23}H_{27}O_6ClNa$

Figure S85. IR spectrum of **11**

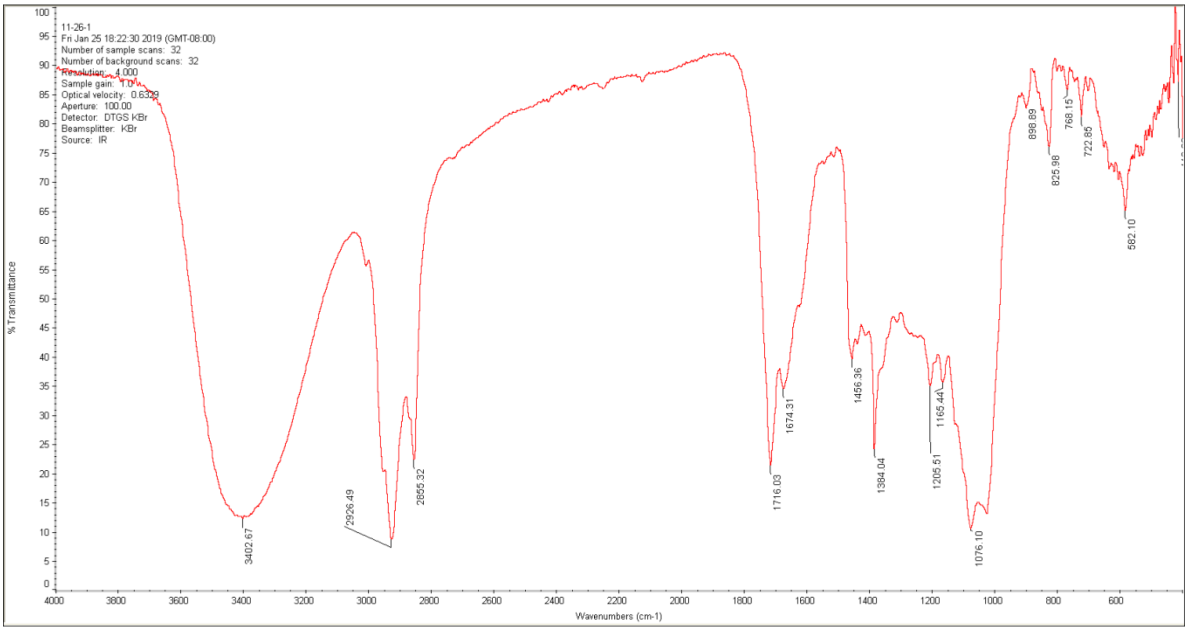

Figure S86. ^{1}H NMR (600 MHz) spectrum of **11** in DMSO-*d*6

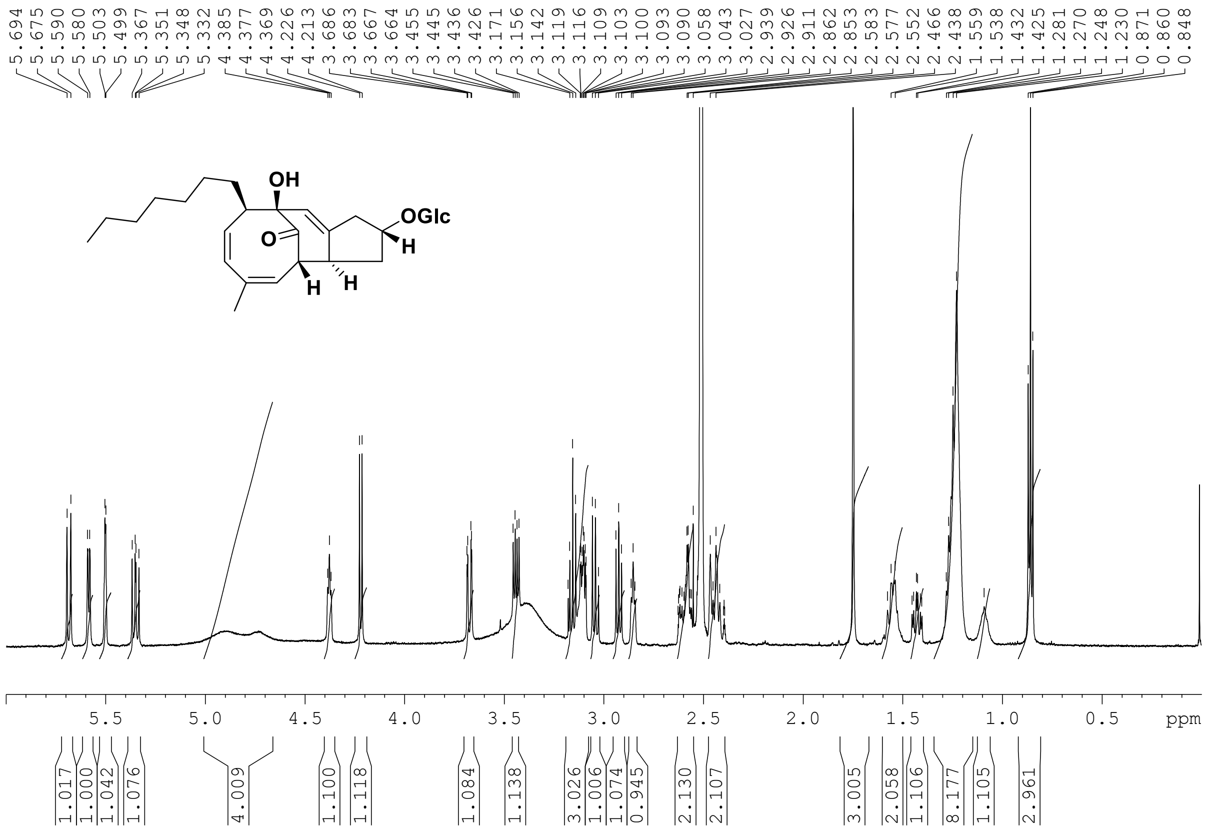

Figure S87. ^{13}C NMR and DEPT (150 MHz) spectra of **11** in DMSO-d_6

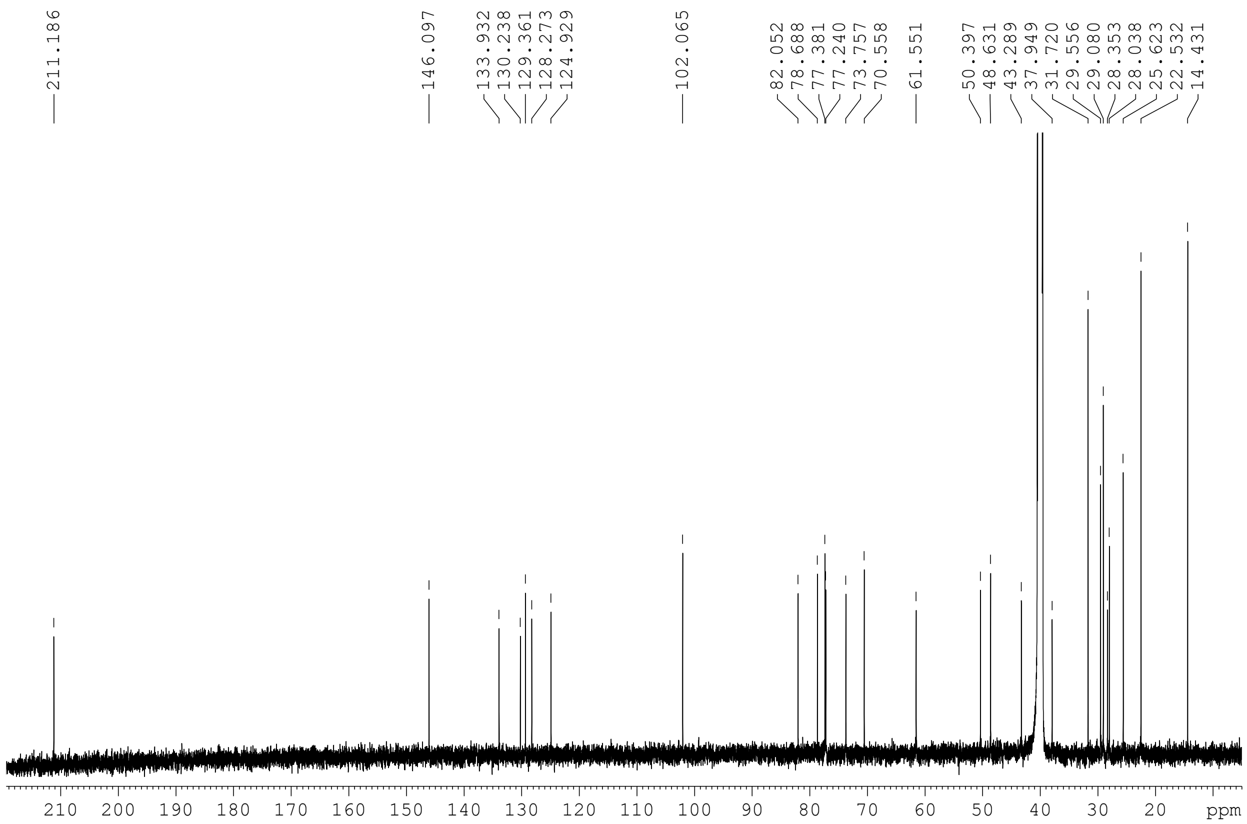

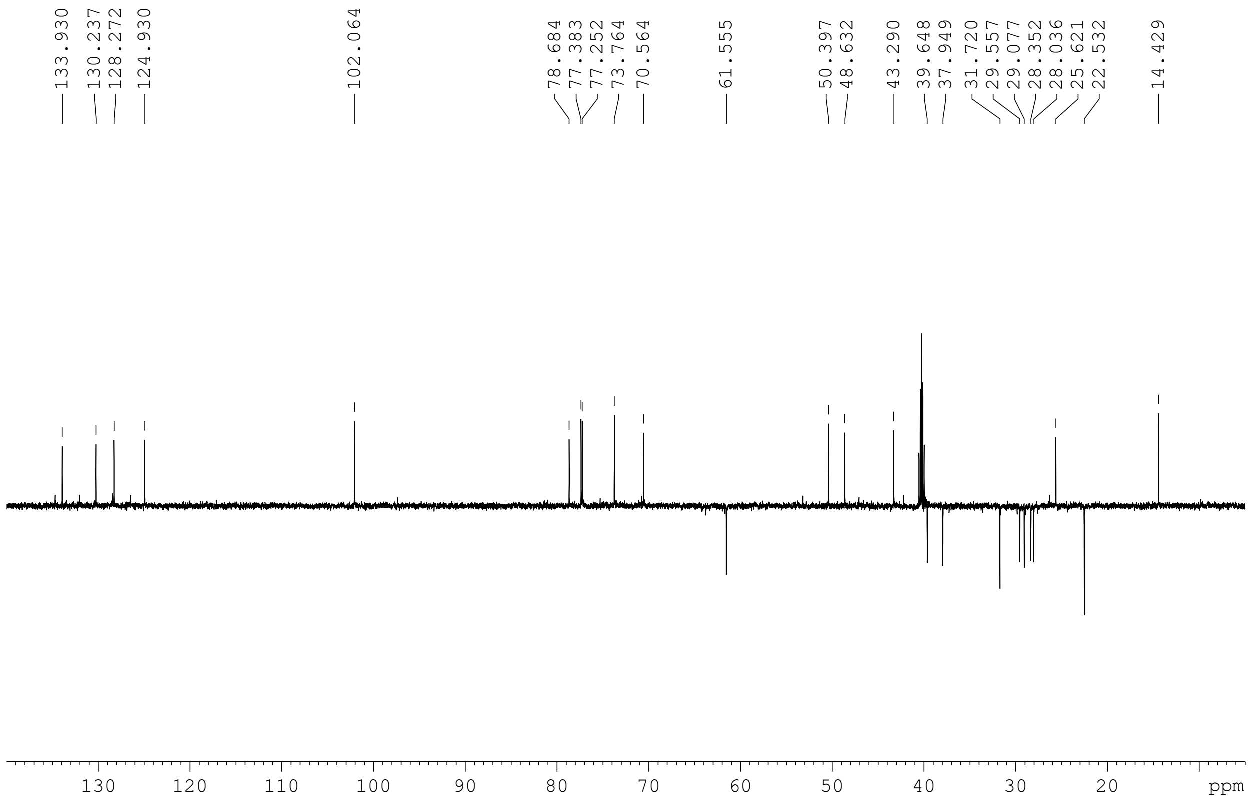

Figure S88. ^{1}H-^{1}H COSY spectrum of **11** in DMSO-*d*6

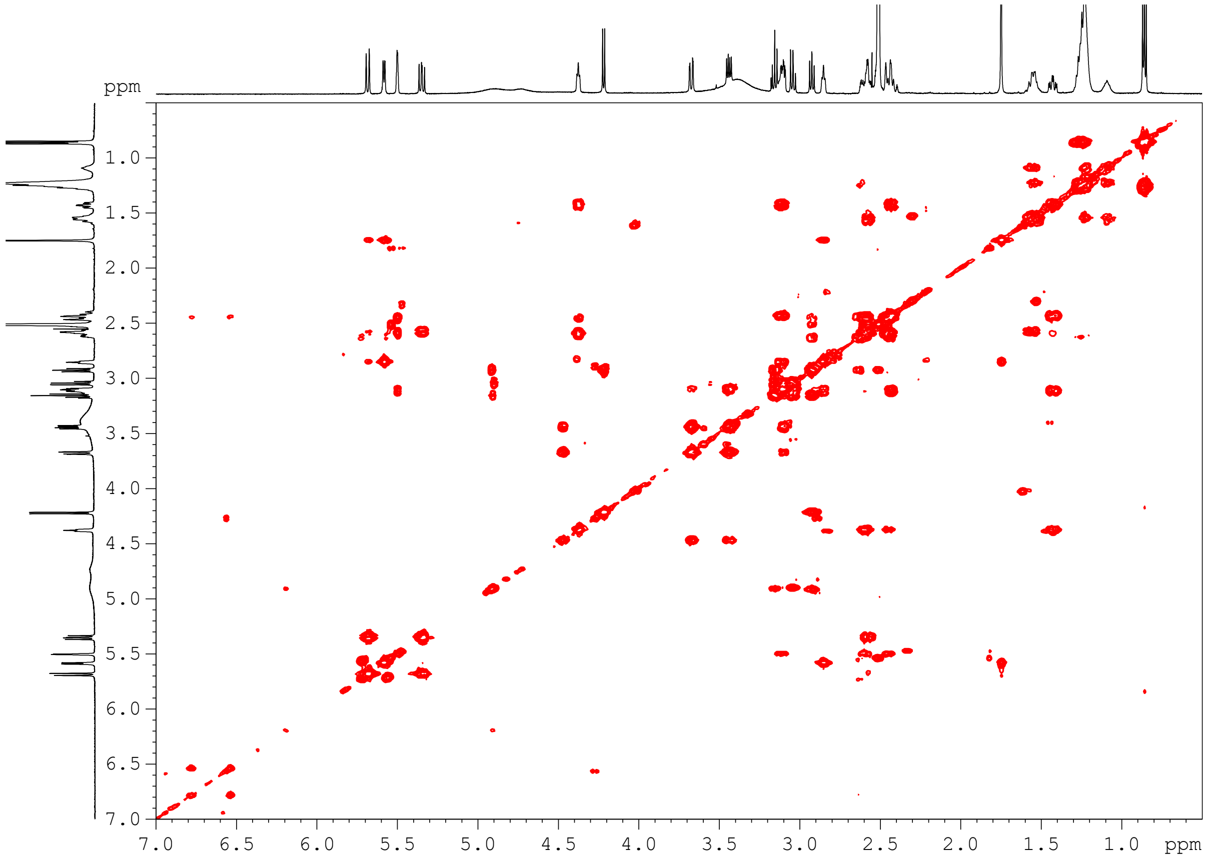

Figure S89. HSQC spectrum of **11** in DMSO-*d6*

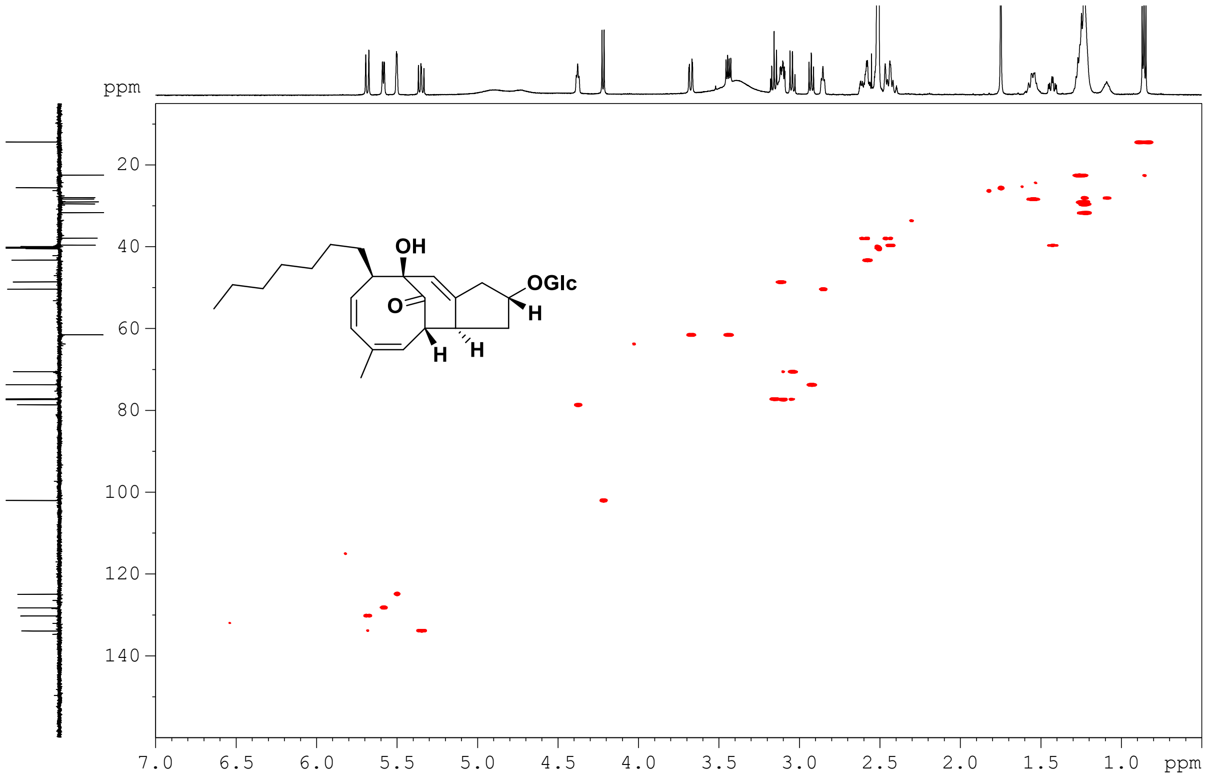

Figure S90. HMBC spectrum of **11** in DMSO-*d6*

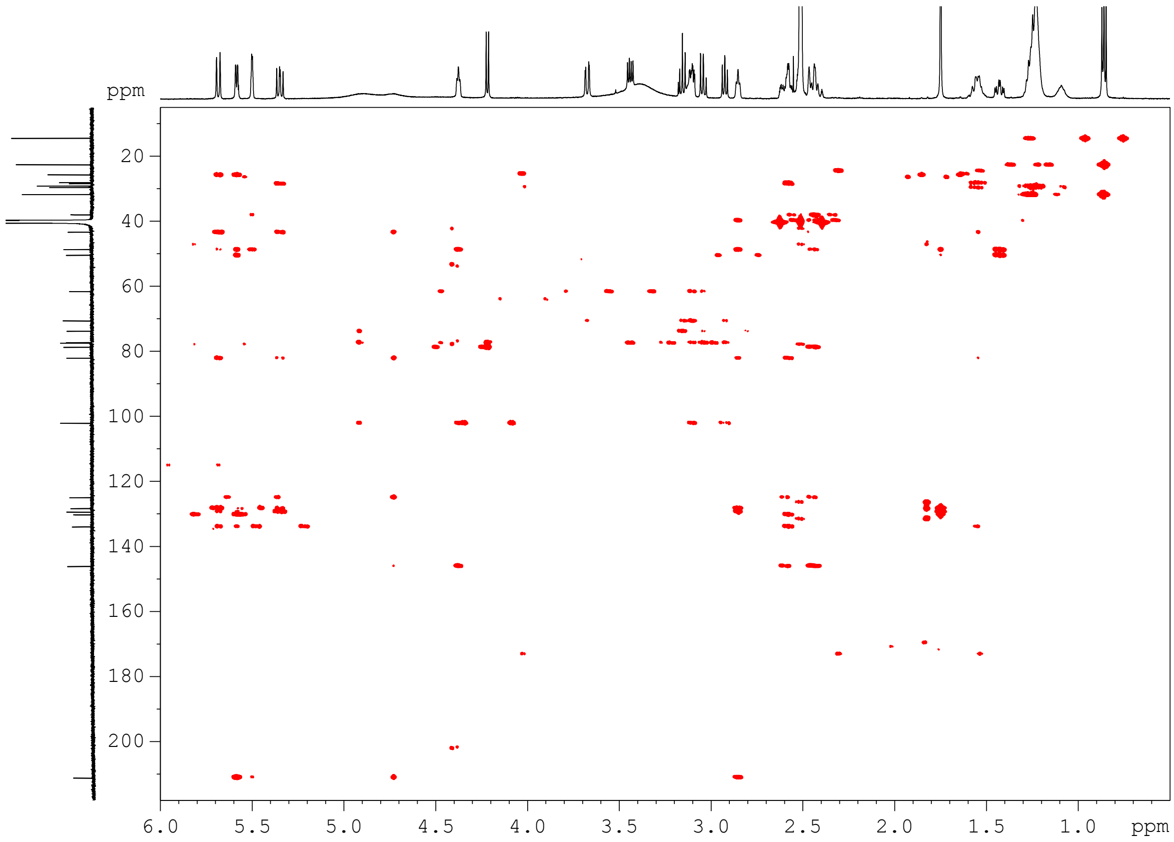

Figure S91. NOESY spectrum of **11** in DMSO-*d*6

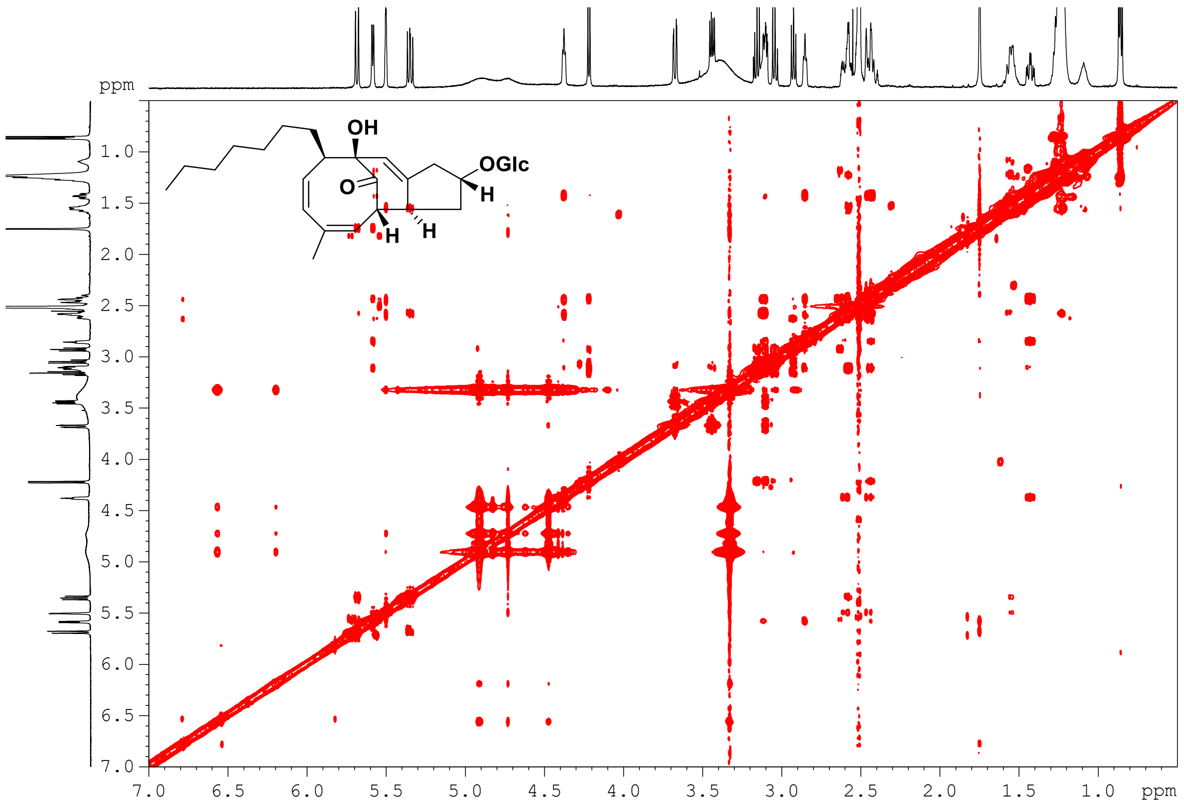

Figure S92. ROESY spectrum of **11** in DMSO-*d*6

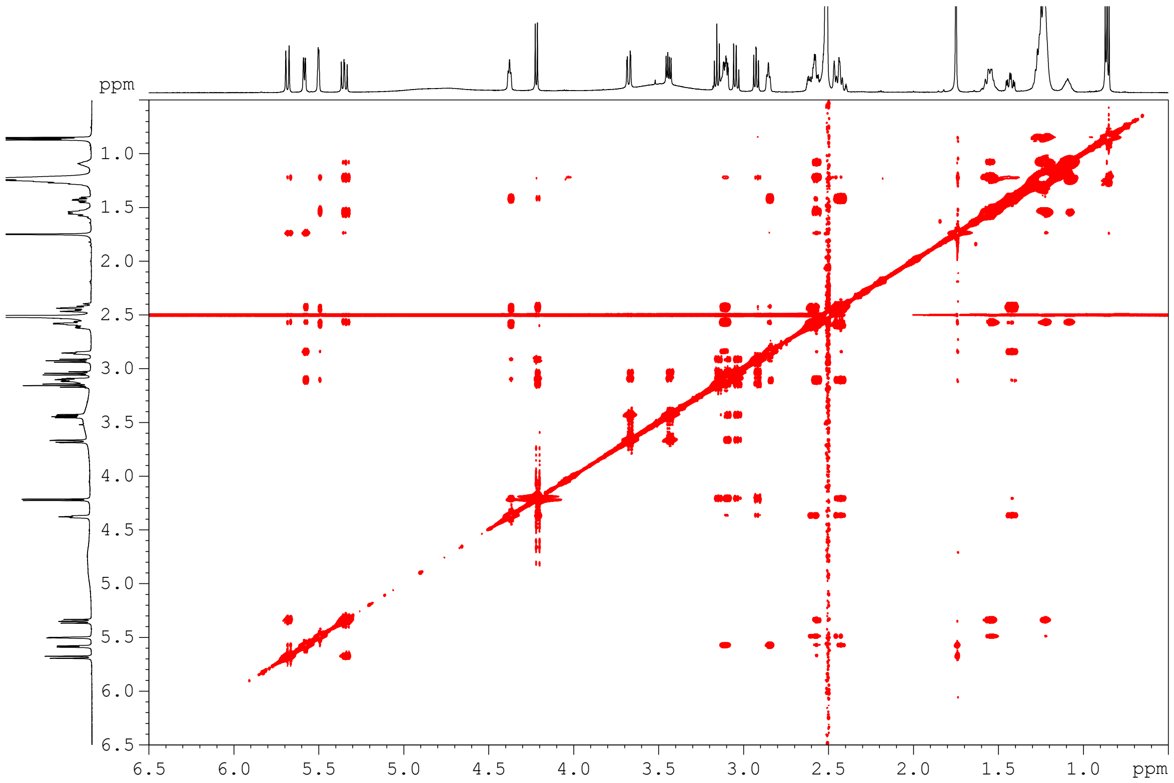

Figure S93. HRESIMS spectrum of **11**

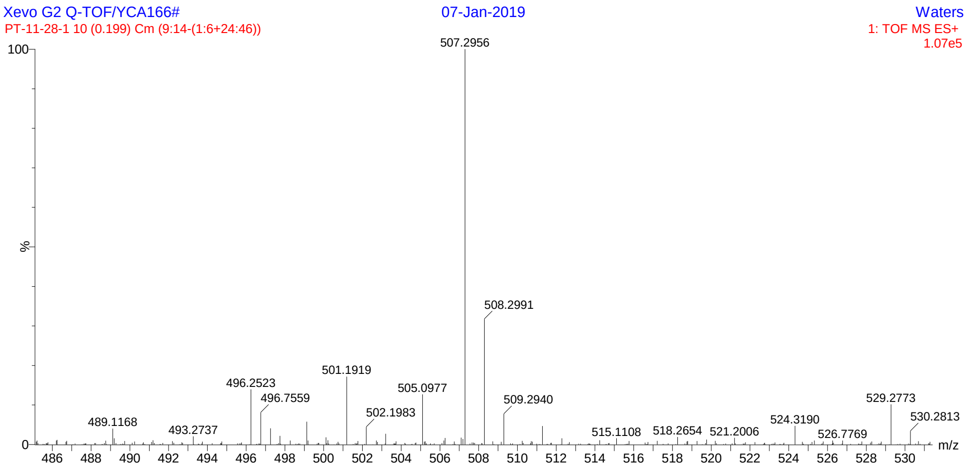

Mass	Calc. Mass	mDa	PPM	DBE	i-FIT	Norm	Conf(%)	Formula
507.2956	507.2958	-0.2	-0.4	7.5	166.9	1.373	25.33	$C_{28}H_{43}O_8$

Figure S94. IR spectrum of **12**

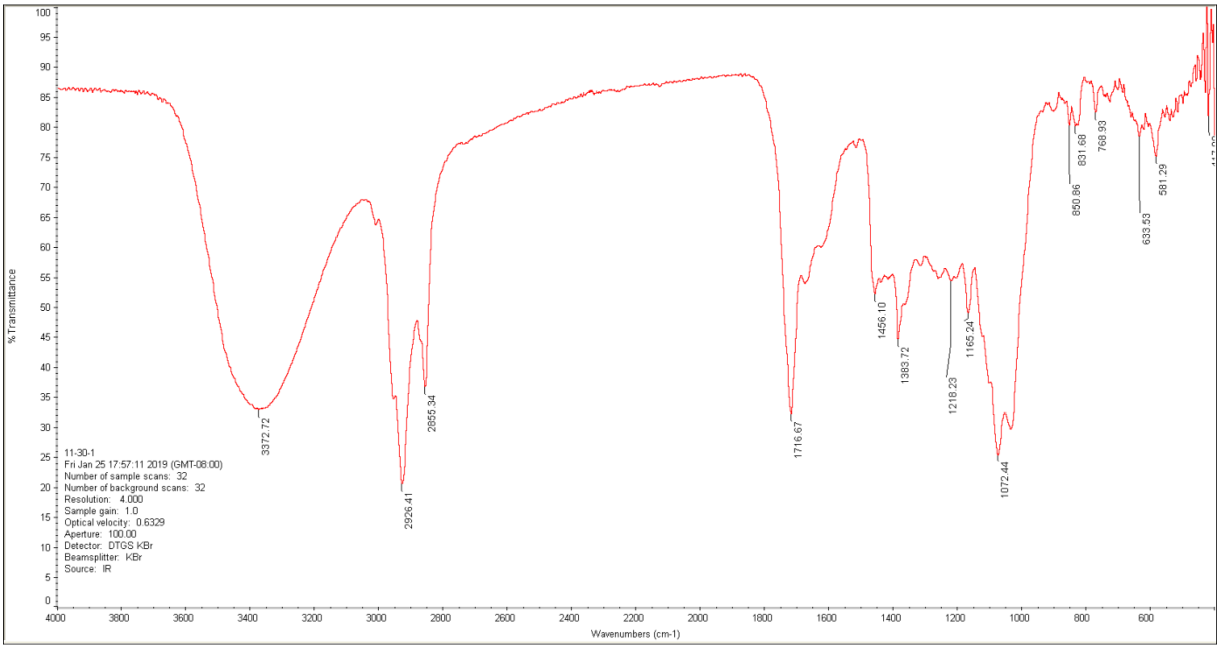

Figure S95. ^{1}H NMR (600 MHz) spectrum of **12** in DMSO-*d6*

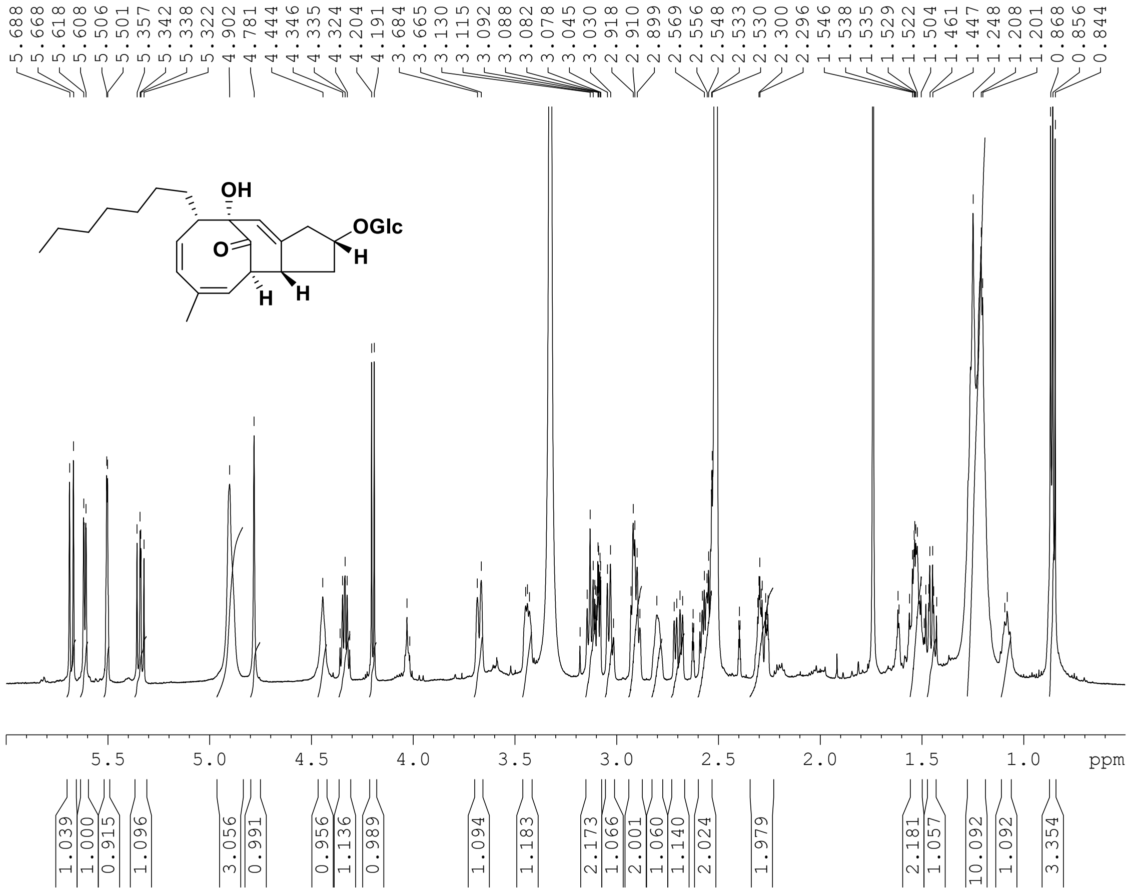

Figure S96. ^{13}C NMR (150 MHz) spectrum of **12** in DMSO-d_6

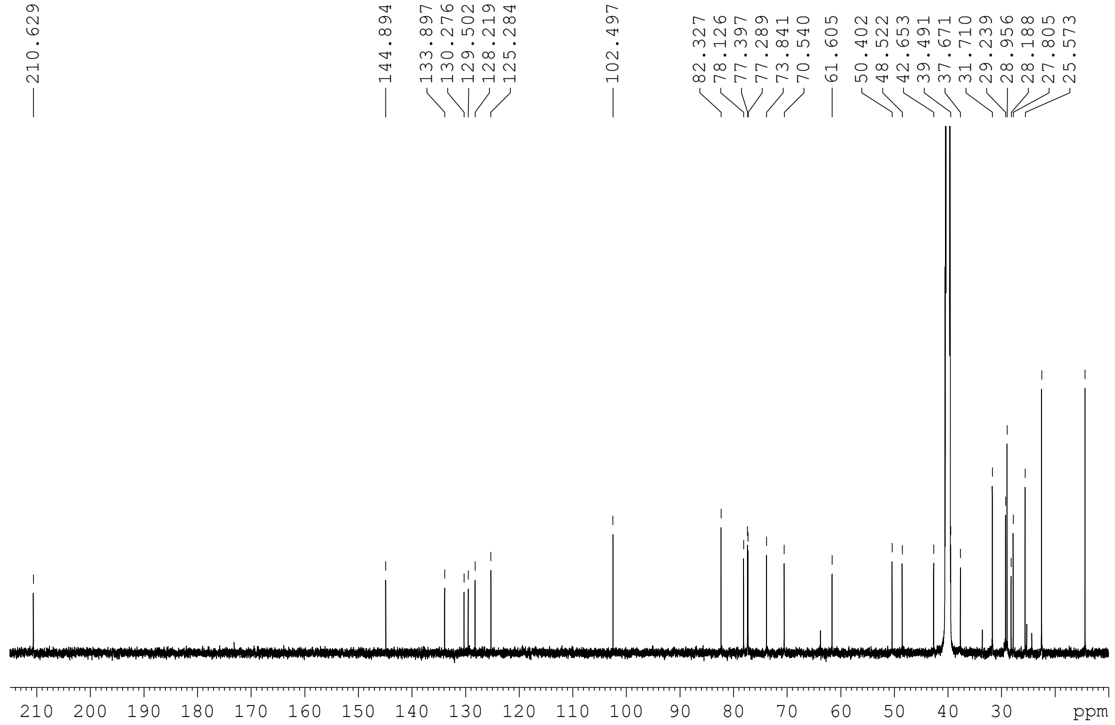

Figure S97. DEPT spectrum of **12** in DMSO-d_6

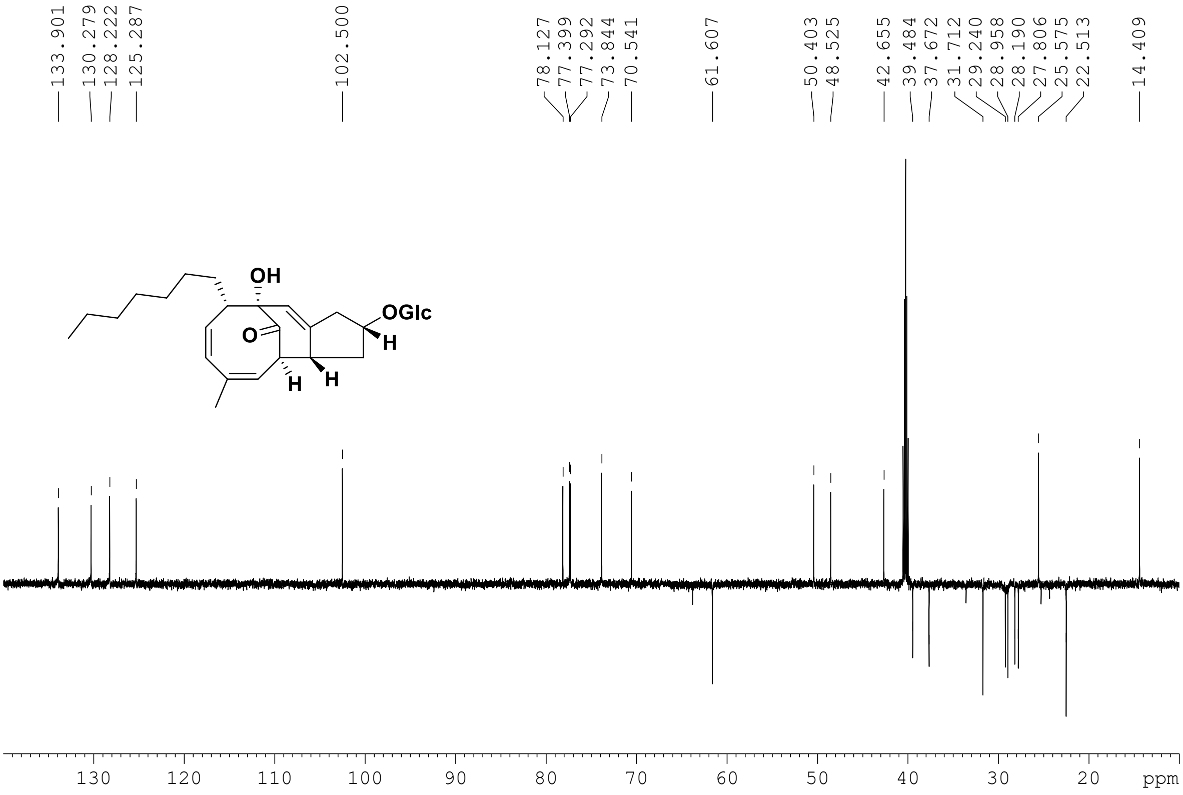

Figure S98. ^{1}H-^{1}H COSY spectrum of **12** in DMSO-d6

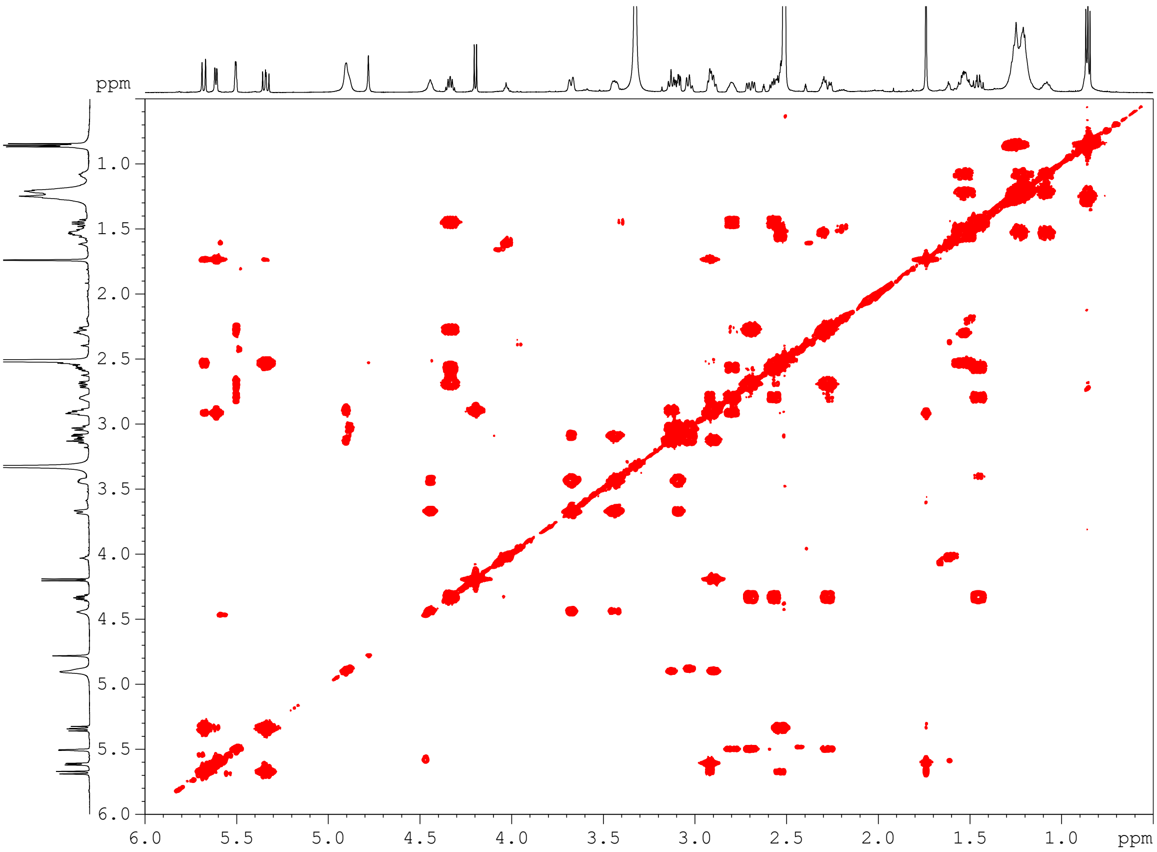

Figure S99. HSQC spectrum of **12** in DMSO-*d*6

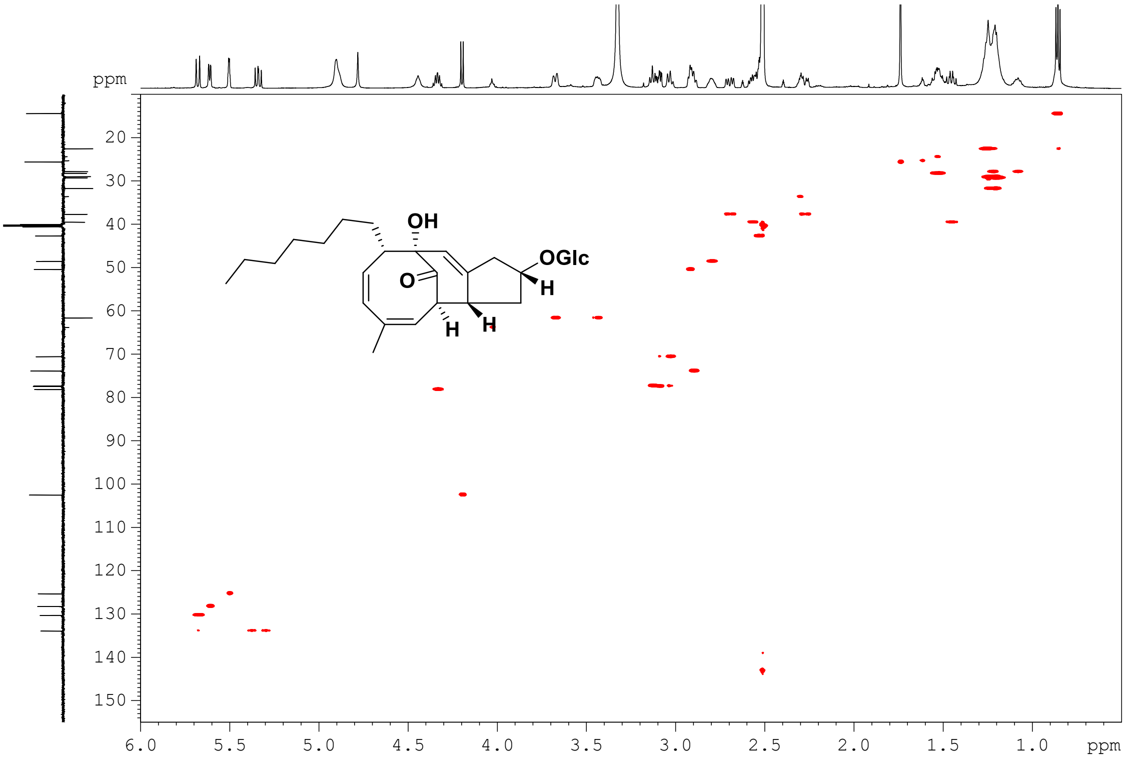

Figure S100. HMBC spectrum of **12** in DMSO-*d*6

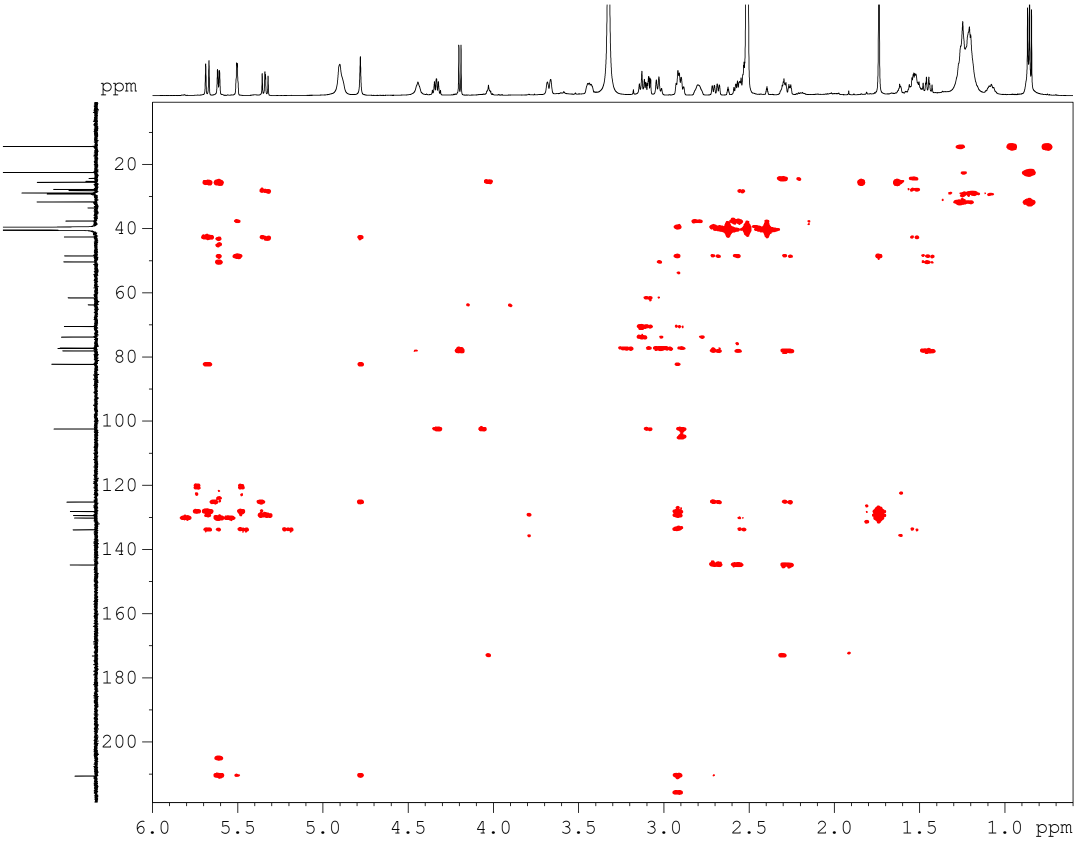

Figure S101. NOESY spectrum of **12** in DMSO-*d*6

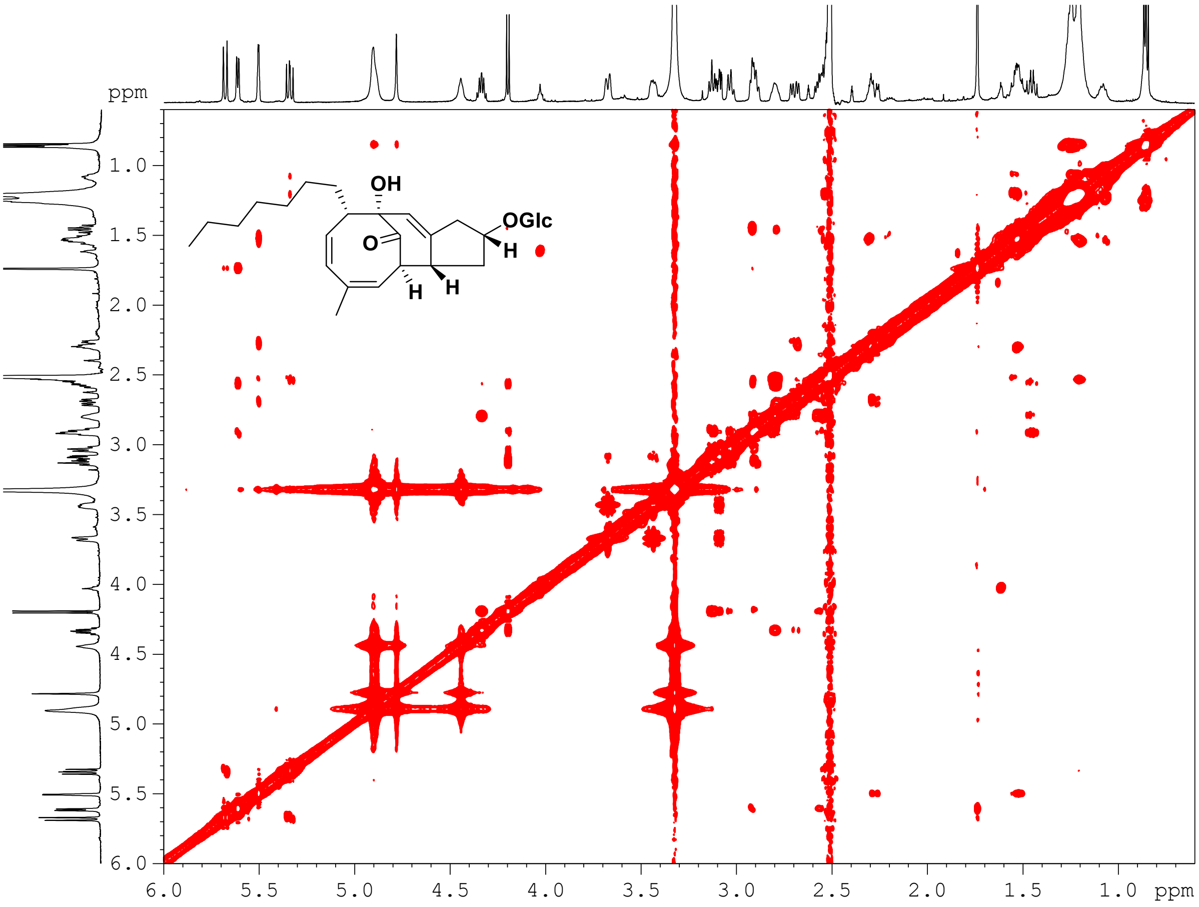

Figure S102. HRESIMS spectrum of **12**

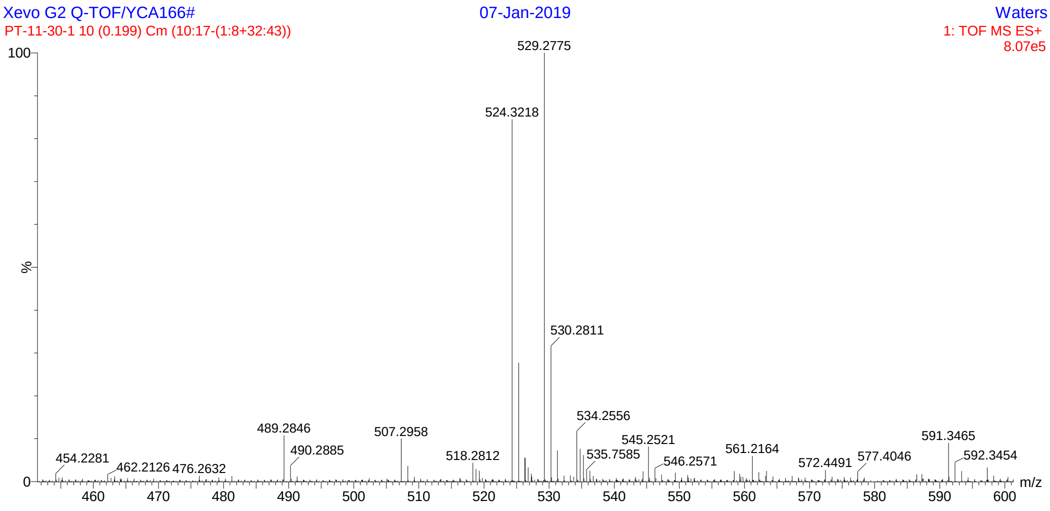

Mass	Calc. Mass	mDa	PPM	DBE	i-FIT	Norm	Conf(%)	Formula
507.2958	507.2958	0.0	0.0	7.5	382.4	6.116	0.22	$C_{28}H_{43}O_8$

Figure S103. Part node clusters of EtOAc extract by molecular networking

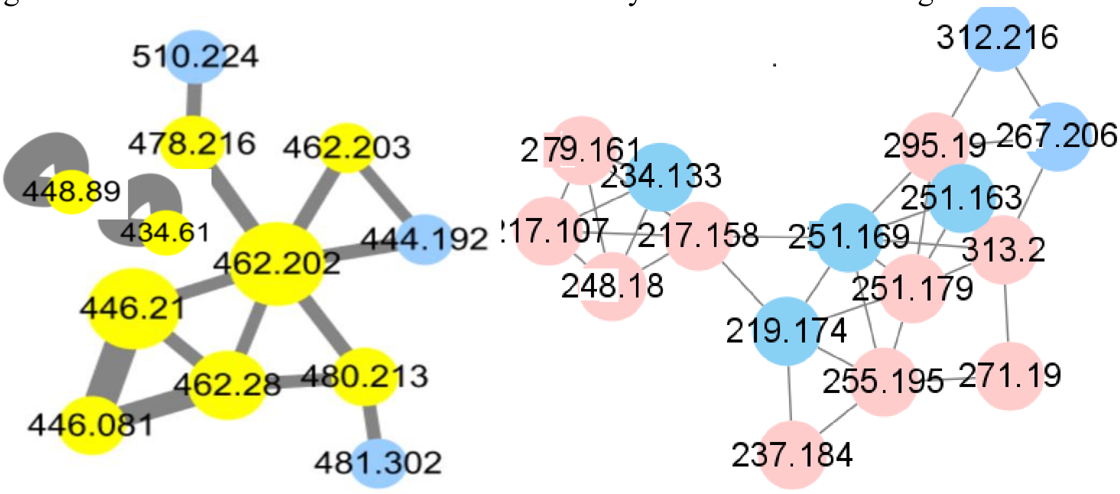

Figure S104. Key HMBC and COSY correlations of **1**–**10**.

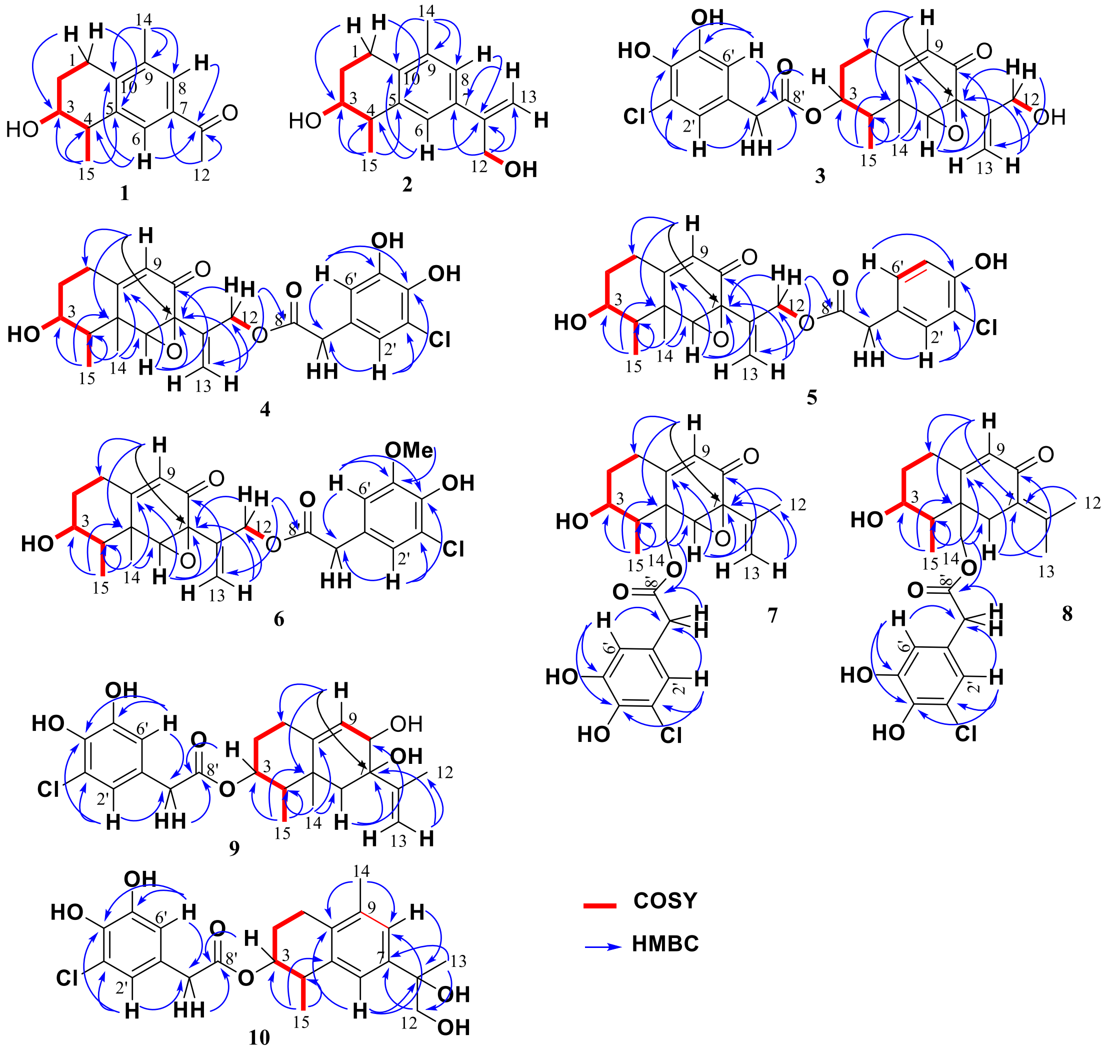

Supplement: Supplementary file 1 [file marinedrugs-20-00712-s001.zip › marinedrugs-2038802-supplementary.pdf]
